# Development of a Lysine‐Reactive Targeted Covalent Inhibitor for the P300/CBP‐Associated Factor Bromodomain Through Structure‐Based Design

**DOI:** 10.1002/cmdc.70301

**Published:** 2026-05-22

**Authors:** Richard R. Ede, Kerstin E. Peterson, Richard K. Begyinah, Irin P. Tom, Jason M. Ochoada, Molly S. Sneddon, Ana Katrina Y. Tiu, Marcus Fischer, Anang A. Shelat, William C. K. Pomerantz

**Affiliations:** ^1^ Department of Chemistry University of Minnesota Minneapolis Minnesota USA; ^2^ Department of Chemical Biology and Therapeutics St. Jude Children's Research Hospital Memphis Tennessee USA

**Keywords:** covalent inhibitors, drug discovery, KAT2B, lysine‐reactive, p300/CBP‐associated factor

## Abstract

Epigenetics is defined by changes in heritable phenotypes that do not involve a change in DNA sequence. P300/CBP‐associated factor (PCAF) is an important epigenetic regulatory protein that can alter chromatin through a histone acetyltransferase domain, while also serving as an epigenetic reader through a C‐terminal bromodomain. PCAF promotes the transcription of the HIV‐1 genome and is implicated in the development of glioblastoma. The currently reported PCAF inhibitors are non‐covalent and require high concentration to maintain target occupancy. Here, we explore a new approach using covalent inhibition. Starting with a lead scaffold (BZ1), test‐molecules were rationally designed for selectively targeting PCAF by installing lysine‐reactive groups onto the lead scaffold to enable covalent bond formation with the nonconserved lysine residue in the PCAF bromodomain. The inhibition, selectivity, and kinetic properties (*k*_inact_/*K*_I_) of these molecules were evaluated using intact protein mass spectrometry, while biophysical and cellular data were employed to verify the covalent mechanism and in‐cell target engagement. After optimization, we developed the first PCAF covalent inhibitor, **10**, which labeled PCAF covalently in vitro and engages PCAF in cells. The covalent inhibitor, **10**, represents a useful starting point for future inhibitor optimization and heterobifunctional molecule development.

## Graphical abstract

We developed a targeted covalent inhibitor for the p300/CBP‐associated factor (PCAF) bromodomain. Our lead molecule, **10**, labeled a unique lysine residue in the PCAF bromodomain, demonstrated potent inhibition in biophysical/biochemical assays, and engaged the PCAF bromodomain in cells. Compound **10** thus serves as a rational starting point in developing covalent inhibitors for PCAF bromodomain that target this unique K753, and covalent inhibitors for other class I bromodomains.

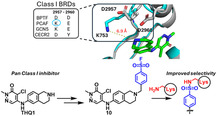

## 1 Introduction

Bromodomains are conserved modules that bind acetylated lysine residues on histone tails which recruit transcriptional complexes to modulate gene expression, or direct enzymatic activity such as histone acetylation [1]. The human proteome has 61 bromodomains across 46 bromodomain‐containing proteins [2]. Of these, the bromodomain and extra‐terminal domain (BET) family proteins (class II bromodomains) are the most studied [3]. Bromodomain‐containingproteins outside this family have received less attention, leading to knowledge gaps in their biological function and lack of probes/inhibitors for these proteins [4].

P300/CBP‐associated factor (PCAF) or lysine acetyltransferase 2B (KAT2B) is a class I family bromodomain‐containing protein. PCAF has three functional domains: an N‐terminal E3‐ligase ubiquitination domain known to degrade HDM2, a histone acetyltransferase domain involved in the activation of the Hedgehog signaling pathway, and a much less understood C‐terminal bromodomain which binds acetylated lysine residues on histone tails [5, 6]. While the PCAF bromodomain was the first bromodomain to be structurally characterized and demonstrated to bind acetylated histones [7], the functional role of this domain is understudied. Several studies have implicated PCAF in diverse disease states like HIV and glioblastoma [8, 9], and previous research suggests that the PCAF bromodomain is highly druggable (Dscore = 1.08) [10]. Consequently, there is a motivation to develop potent and selective inhibitors of the PCAF bromodomain, as either chemical probes to evaluate the functional significance of the bromodomain inhibition, or as new ligands for developing heterobifunctional molecules like acetylation targeting chimeras (AceTACs) [11].

**FIGURE 1:**
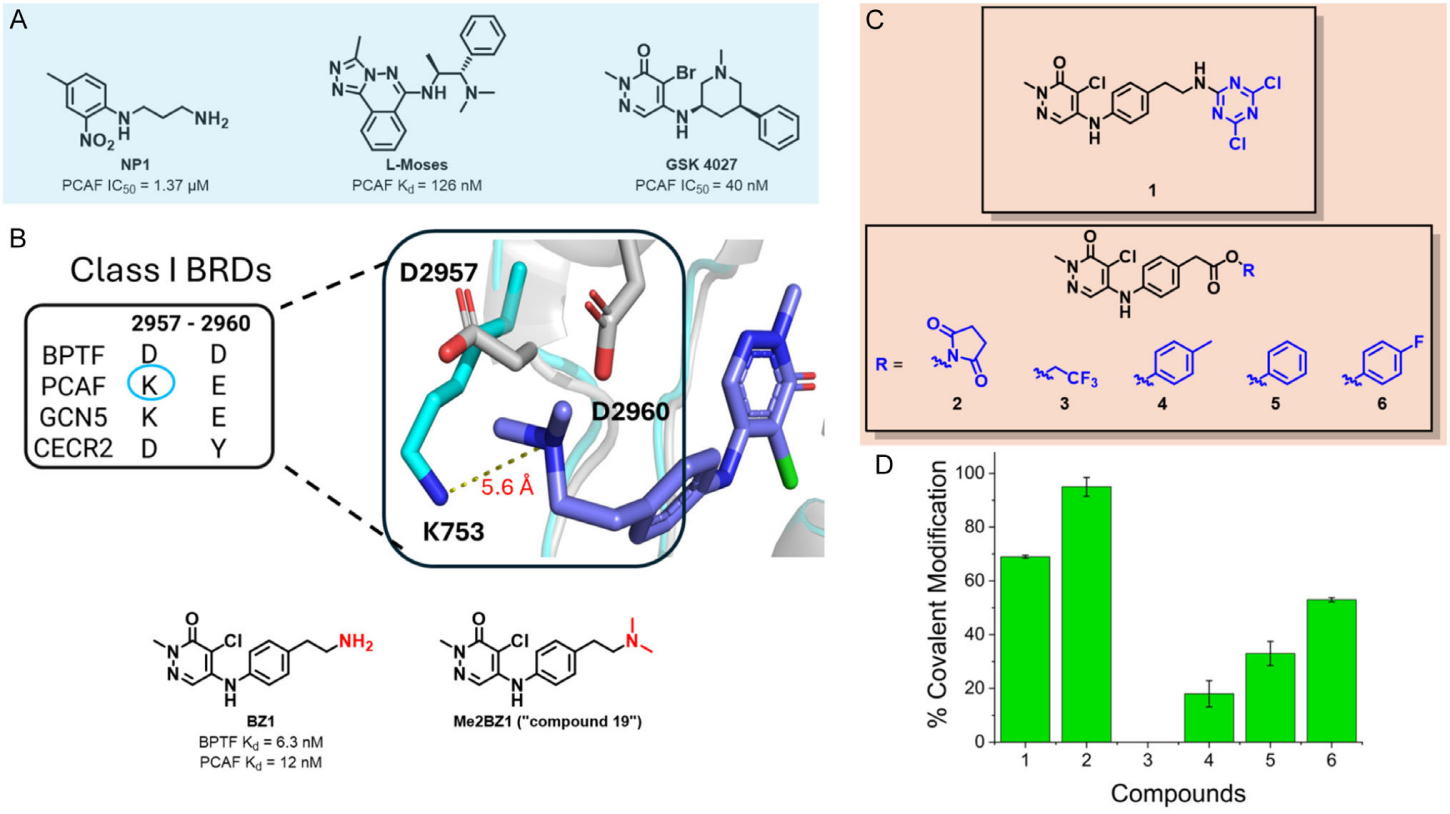
(A) Selected non‐covalent inhibitors of PCAF. (B) Overlay of the crystal structure of the BPTF‐Me2BZ1 complex (grey, PDB: 7M2E) and that of PCAF bromodomain (cyan, PDB: 6J3O) with key residues highlighted. Numbering of amino acid residues is based on the BPTF sequence. BZ1 and Me2BZ1 structures are below. (C) BZ1‐based electrophiles. Electrophilic warheads are shown in blue. (D) Percent labelling of PCAF bromodomain by compounds **1**–**6** determined by intact protein mass spectrometry after 24 h incubation ([PCAF]:[TCI] = 1:1.5, *n* ≥ 2).

Several PCAF inhibitors have been reported, including NP1, GSK4027, and L‐Moses (Figure 1A) [12, 13, 14]. A PCAF Proteolysis Targeting Chimera (PROTAC) has also been disclosed based on GSK4027 [15]. However, to the best of our knowledge, all PCAF inhibitors are noncovalent. Recently, the rational design of irreversible covalent inhibitors has gained attention, leading to several Food and Drug Administration approvals (e.g., vigabatrin, afatinib, ibrutinib, and sotorasib) [16, 17, 18, 19]. It has been reported that the use of irreversible covalent inhibitors increases favorability of the protein‐bound form, which could lead to higher potencies and ligand efficiencies when compared to non‐covalent inhibitors [20]. A targeted covalent inhibitor (TCI) is made up of two parts: a reversibly binding motif that binds to the protein noncovalently, and a warhead/electrophile which, after the reversible binding event, reacts with a nucleophilic amino acid residue on the protein [21]. A covalent inhibitor can confer benefits, including better selectivity, higher potency, and convenient dosing as the protein function is not restored until resynthesis [22]. Most covalent drugs/inhibitors react with a cysteine residue on their target protein [23]. While targeting cysteine is advantageous due to its rarity and ideal reactivity at physiological pH, there is a growing interest in developing covalent inhibition beyond cysteine targeting, including nucleophilic amino acids, lysine, tyrosine, and histidine [24]. Supporting this approach, a lysine‐reactive TCI of class II bromodomains was recently reported [25].

Recently, we reported a series of noncovalent inhibitors of a related class I bromodomain, BPTF [26]. Data from a BROMOscan assay showed that one such inhibitor, BZ1, retained off‐target affinity for the PCAF, GCN5, and CECR2 bromodomains (BPTF *K*_d_ = 6.3 nM, PCAF *K*_d_ = 12 nM, GCN5 *K*_d_ = 44 nM, CECR2 *K*_d_ = 11 nM, Figure 1B). We solved an X‐ray crystal structure of BPTF bound to a related inhibitor, Me2BZ1 (also called compound 19), which is structurally similar to BZ1. An overlay of the BPTF‐Me2BZ1 structure with that of PCAF revealed a unique lysine residue, K753, in the ZA loop region of PCAF bromodomain within reach of the aliphatic amine in Me2BZ1 (Figure 1B) [26]. Only 4 of the 46 bromodomain‐containing proteins in the human proteome have this unique lysine, including the GCN5 bromodomain [2]. Here, we develop the first lysine‐reactive TCI that is selective for the class I bromodomains with biased selectivity for PCAF and its paralog, GCN5. We hypothesized that by installing lysine‐reactive warheads/electrophiles onto the primary amine of BZ1‐analogs, we could develop a selective covalent inhibitor that targets K753 in the PCAF bromodomain. However, the reactivity of this surface‐exposed, high‐pKa lysine had not been established. We strategically installed lysine‐reactive warheads with a broad range of reactivity onto the primary amine of BZ1. Our two lead molecules, **5** (Figure 1) and an improved sulfonylfluoride discussed below, were shown to covalently label the PCAF bromodomain using intact protein mass spectrometry. Tandem mass spectrometry confirmed that these molecules label the unique K753 in the PCAF bromodomain. Given the low reactivity of **5**, we advanced with the sulfonylfluoride and assessed its ability to engage PCAF in biophysical and cellular assays. This work identifies two PCAF covalent inhibitors and establishes the ligandability of the unique K753 in the PCAF bromodomain. More selective electrophiles such as squarates will be further investigated. We believe this will inform future endeavors focused on selectively targeting PCAF bromodomain to fully understand its role in disease.

## 2 Results and Discussion

**FIGURE 2:**
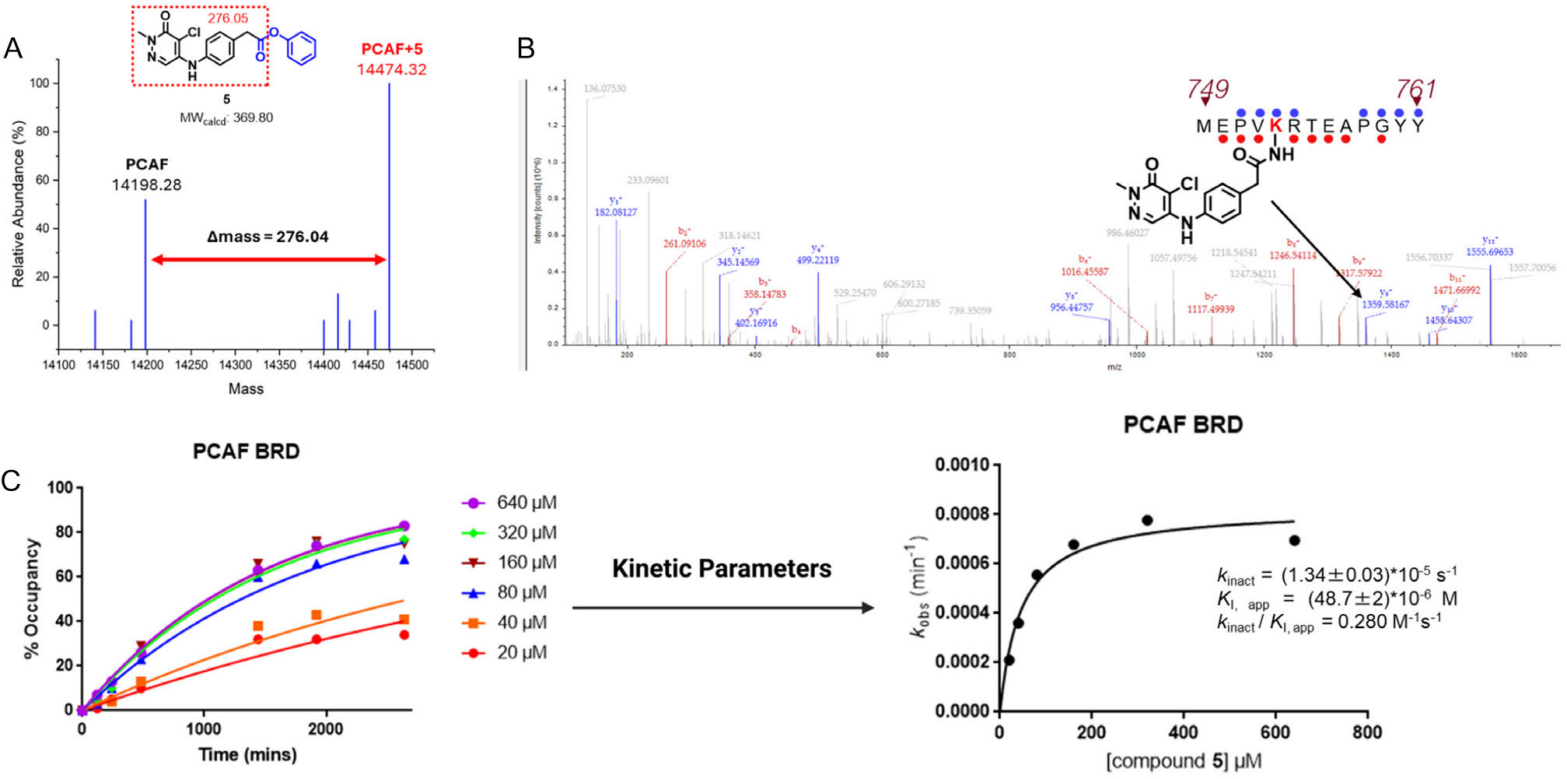
(A) Sample intact protein MS data obtained after treating PCAF bromodomain with **5** (B) Tandem MS data of compound **5**‐labeled peptide derived from PCAF bromodomain with K753 shown in red. (C) Labeling of PCAF bromodomain (20 µM) was assessed at different concentrations of **5** over time. Percentage total labeling data from time course intact MS were then fit to a modified Michaelis‐Menten to obtain the *k*_inact_ and *K*_I_ (*n* = 2).

Surface‐exposed lysine residues typically have a p*K*_a_ of 10.4 [27, 28]. Accordingly, the vast majority of these lysine residues will be in the protonated unreactive form at physiological pH 7.4. Thus, we explored lysine‐reactive warheads possessing a broad range of reactivity. Initially, we installed the highly reactive electrophiles dichlorotriazine and N‐hydroxysuccinimide ester on BZ1‐analogs to furnish **1** and **2** (Figure 1C). Data obtained by intact protein mass spectrometry showed covalent labeling of PCAF by these molecules supporting the presence and accessibility of a reactive lysine residue (Figure 1D). However, a significant amount of a double adduct was observed. These two warheads were not further pursued. It has been shown that trifluoroethyl esters and phenyl esters could also be used to selectively label lysine residues [29]. Inspired by this, we turned our attention to the less reactive warheads, trifluoroethyl‐, *p*‐cresol‐, phenyl‐, and monofluorophenyl esters for compounds **3, 4**, **5**, and **6** (listed in order of increasing reactivity, Figure 1C). Compounds **4**, **5**, and **6** labeled the PCAF bromodomain covalently, with only a mono‐labeled adduct observed after 24 h (Figures 1D and 2A). Compound **3** did not covalently label PCAF which may indicate a threshold for reactivity. Of the three phenyl esters (**4**–**6**), we chose **5** for further analysis due to its intermediate reactivity which is ideal for fast labeling while avoiding potential off‐targets.

To identify the residue being labeled by **5** on PCAF, tandem mass spectrometry of chymotrypsin‐digested peptides was employed. Of the digested peptides characterized covering 71% of the PCAF bromodomain, compound **5** was shown to uniquely label K753. No additional amino acid labeling was observed (Figure 2B). However, data obtained from in‐solution digestion of the PCAF‐**1** adduct showed that **1** labels both K753 and K785 on PCAF (data not included). These results confirmed our hypothesis that a mildly reactive electrophile could react with a nearby surface‐exposed lysine despite its high calculated pKa value of 10.7 (calculated with PROPKA, Schrodinger, Figure S10), using a high affinity PCAF bromodomain ligand.

Targeted covalent inhibition occurs in two steps: First, the molecule binds to the protein noncovalently using the reversibly binding motif, placing the warhead near the nucleophilic amino acid of interest. This is followed by a proximity‐driven covalent reaction between the warhead and the targeted amino acid. For amino acids like lysine which are only present in a low population of their reactive form, long residence times are needed to allow a subsequent reaction between the electrophilic warhead and nucleophilic amino acid. To evaluate the potency of a covalent inhibitor, efficiency of covalent bond formation (*k*_inact_/*K*_I_) is the recommended parameter as it accounts for both steps [30]. The *k*_inact_ is the maximum rate of covalent bond formation, while *K*_I_ is the concentration of the covalent inhibitor at which the rate constant is $\frac{1}{2}$ *k*_inact_, analogous to Michaelis constant (K_M_) [31]. Here, we employed time‐dependent intact protein mass spectrometry to determine *k*_inact_/*K*_I_ as reported previously [30]. Compound **5** had an average *k*_inact_ of 1.34 × 10^−5^ s^−1^ with an average *K*_I_ of 48.7 × 10^−6^ M, giving a *k*_inact_/*K*_I_ of 0.28 M^−1^ s^−1^ making **5** our first‐generation PCAF bromodomain covalent inhibitor (Figure 2C). However, unlabeled protein was still present in the sample after 24 h of incubation, likely due to hydrolysis of the phenyl ester warhead in **5** as others have observed [32, 33], improper orientation of the warhead toward the targeted lysine residue, or low inherent reactivity of the phenyl ester warhead. Thus, we then focused on developing an optimized second‐generation covalent inhibitor for PCAF.

**FIGURE 3:**
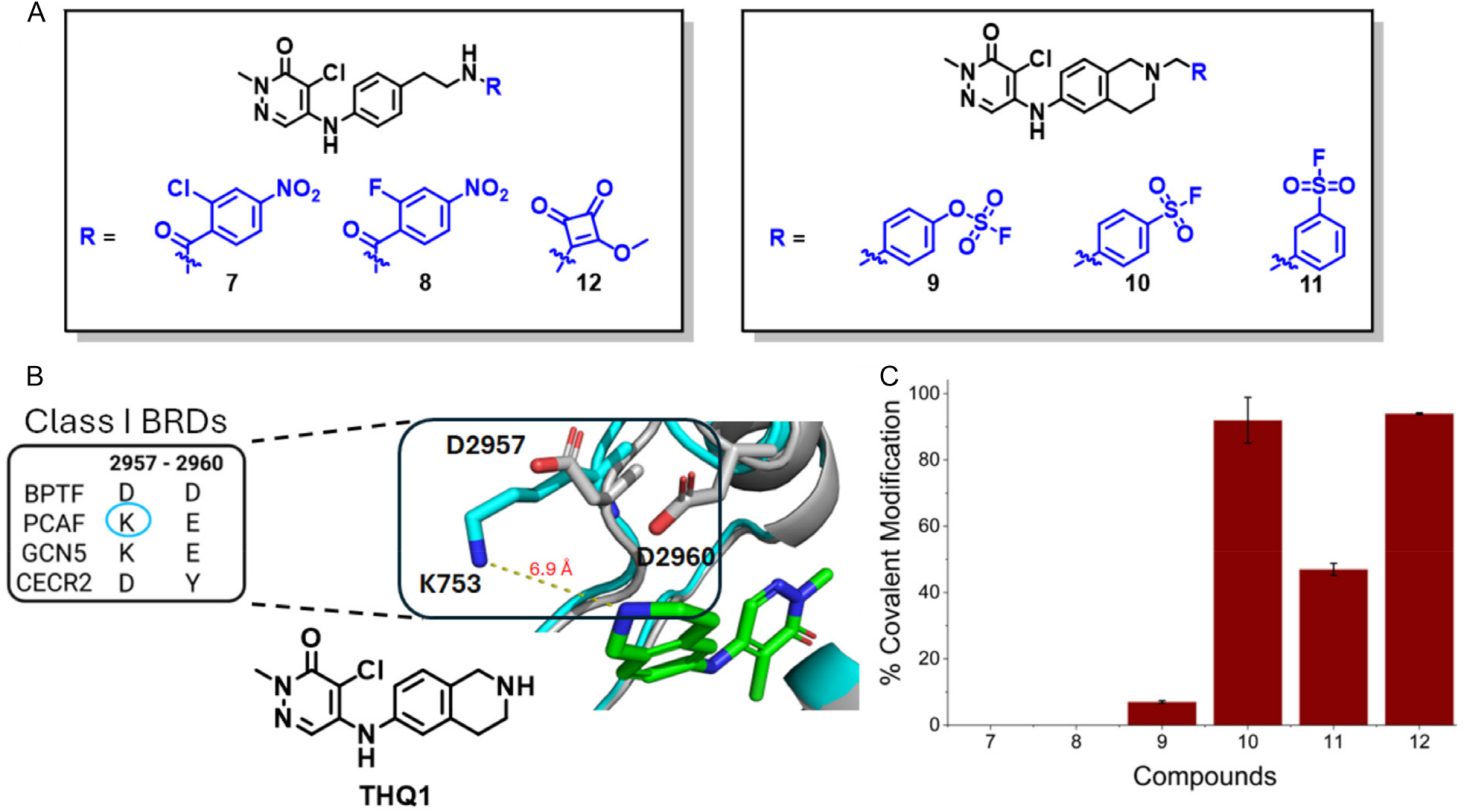
(A) Structures of covalent inhibitors synthesized for optimization of **5**. The warheads are in blue. (B) Crystal structure of BPTF‐THQ1 (gray, PDB: 7RWQ) overlaid on PCAF bromodomain (cyan, PDB: 6J3O). Structure of THQ1 is shown. (C) Percent labeling of PCAF bromodomain by compounds **7**–**12** determined by intact protein mass spectrometry after 24 h incubation ([PCAF]:[TCI] = 1:1.5, *n *≥ 2).

**FIGURE 4:**
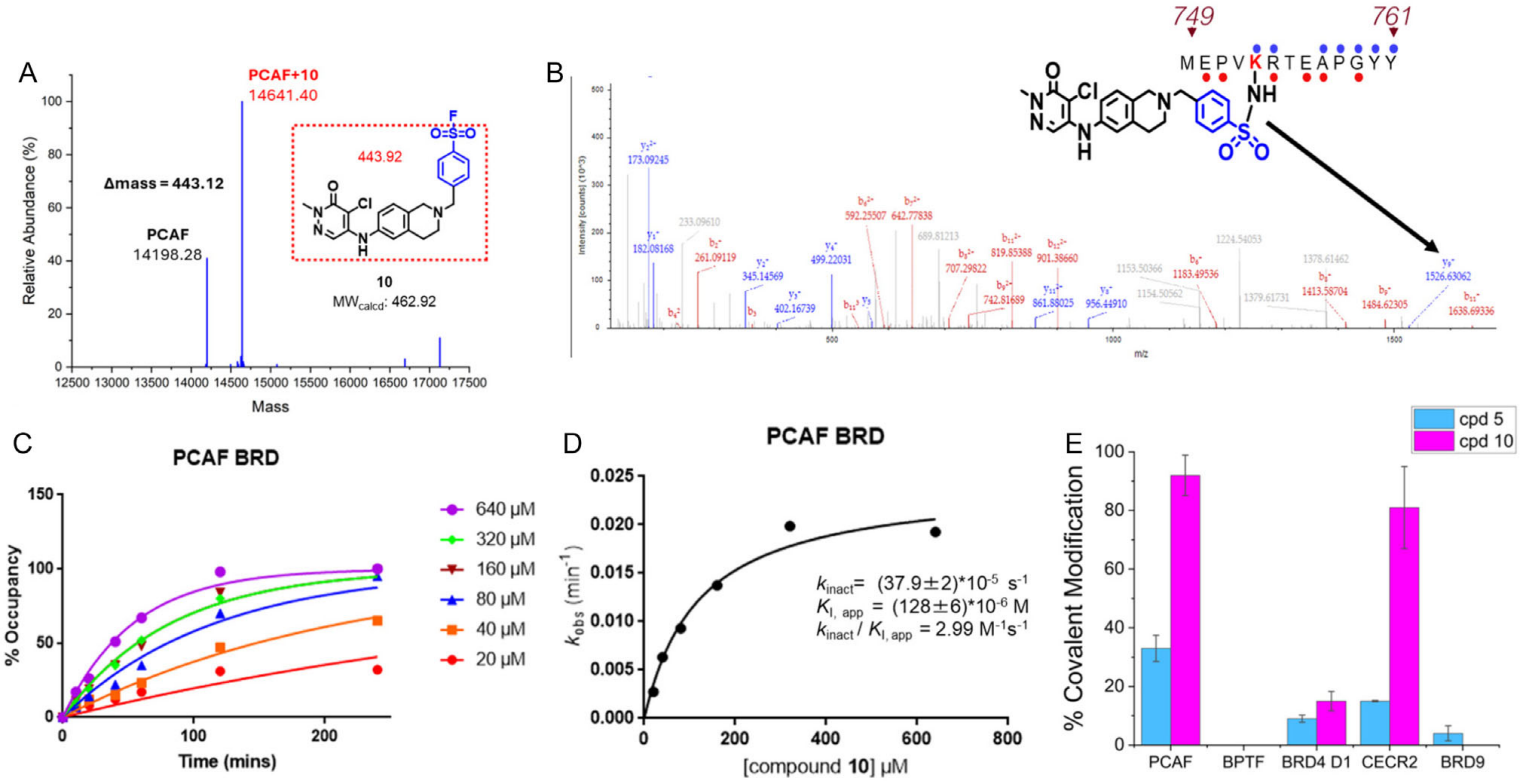
(A) Sample data obtained from intact protein mass spectrometry showing that **10** labels the PCAF bromodomain. (B) MS2 analysis of peptides obtained from chymotrypsin digestion of the PCAF‐**10** adduct shows that K753 (highlighted in red) was labeled. (C) Time course data from intact MS after PCAF was treated with various concentration of **10**. (D) Percentage total labeling data from time course intact MS were then fit to a modified Michaelis–Menten to obtain the *k*_inact_ and *K*_I_ (*n* = 2). (E) Selectivity comparison of **5** and **10** against BPTF, BRD4, CECR2, and BRD9 using intact protein MS (*n *≥ 2).

To rationally design this second‐generation covalent inhibitor for PCAF, we explored new warheads reported for selectively targeting lysine, including halogenated nitrobenzenes (**7** and **8**) [34], aryl fluorosulfates (**9**) [35], and sulfonylfluorides **10** and **11** (Figure 3A) [36]. We also explored a more rigid PCAF reversible binding motif called **THQ1** which has a constrained tetrahydroisoquinoline (Figure 3B). Assessing the crystal structure of BPTF‐**THQ1** overlaid on the PCAF bromodomain shows that **THQ1** has a similar binding pose to the bromodomain as BZ1 (Figure 3B). Based on this, we installed aryl fluorosulfate and aryl sulfonylfluoride warheads onto **THQ1** for **9, 10,** and **11** (Figure 3A,B). Compounds **10** and **11** were shown to label the PCAF bromodomain covalently, with **10** having a higher reactivity (Figures 3C and 4A). Compound **9** labeled PCAF covalently but had a slow rate of reaction (<10% after 24 h). This slow reactivity data is in agreement with reports by others which show the lower electrophilicity of the aryl fluorosulfate compared with the aryl sulfonylfluoride, likely due to resonance stabilization of the sulfur atom by the added oxygen in fluorosulfates [35]. No labeling was observed for **7** and **8**; however, a squarate analog **12** labled to similar levels as **10**.

Given the higher reactivity of sulfonylfluorides over squarates, tandem mass spectrometry using chymotrypsin‐digested PCAF was used to determine the amino acid on the PCAF bromodomain labeled by **10**. Analysis of the resulting peptides revealed that **10** labels K753 on PCAF, with no other amino acid residue labeled (Figure 4B). Here, the digested peptides had a 100% coverage of PCAF bromodomain, giving confidence that a second site of labeling was not missed. Assessing the *k*_inact_/*K*_I_ of **10** using time‐dependent intact protein mass spectrometry showed that **10** has a *k*_inact_/*K*_I_ of 2.99 M^−1 ^s^−1^, about 10 times more than that of **5** (Figure 4C,D). Lysine reactive electrophiles have a broad range of *k*_i__nact_/*K*_i_ values. A recent report of a lysine‐reactive TCI for the class II bromodomains had a 2‐ethynylbenzaldehyde‐based lead molecule with *k*_inact_/*K*_I_ of 1833 M^−1 ^s^−1^ [25]. Other kinact/KI values for lysine‐reactive TCIs include a squarate‐based TCI for aurora kinase A (45.6 M^−1^s^−1^) [37] and a sulfonylfluoride‐based TCI for eukaryotic translation initiation factor 4E (5500 M^−1^ s^−1^) [38].

Next, we investigated the covalent selectivity of **5** and **10**. We tested **5** and **10** against a panel of bromodomain‐containing proteins using intact protein mass spectrometry, including the closest class I off‐targets of BZ1, BPTF and CECR2, class IV bromodomain, BRD9, and class II bromodomain, BRD4. While compound **10** exhibited a higher labeling for PCAF compared to **5**, **10** maintained off‐target activity to CECR2 (Figure 4E). While we have not identified the site of covalent labeling on CECR2, sulfonylfluorides are known to also react with tyrosine residues found in the same loop (ZA loop) as K753 in PCAF [39]. Future work will explore other electrophiles like squarates (compound **12**, Figure 3A,C) as these warheads are reported to react selectively with amine nucleophiles over alcohols [40].

**FIGURE 5:**
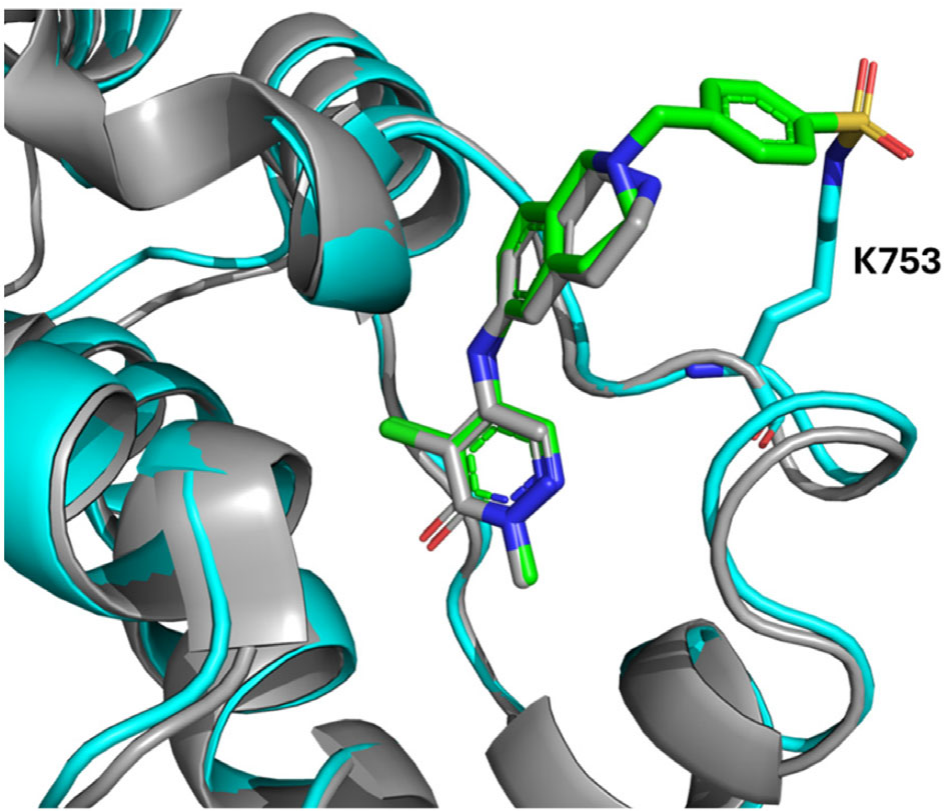
Docked binding pose of **10** covalently reacted with K753 (cyan) overlaid on cocrystal structure of BPTF‐THQ1 (gray, PDB: 7RWQ).

Attempts to crystallize the PCAF‐**10** covalent adduct have been unsuccessful. Consequently, we used covalent docking to determine a probable binding mode of the PCAF‐**10** adduct. An overlay of the docked PCAF‐**10** adduct and BPTF‐THQ1 structure shows both structures are in agreement (Figure 5). The K753 has a partially distorted geometry and suggests that next generation of inhibitors should be optimized to accommodate a more idealized geometry for K753. An overlay of the docked PCAF‐**5** adduct and BPTF‐Me2BZ1 also shows this K753 distortion (Figure S13).

**FIGURE 6:**
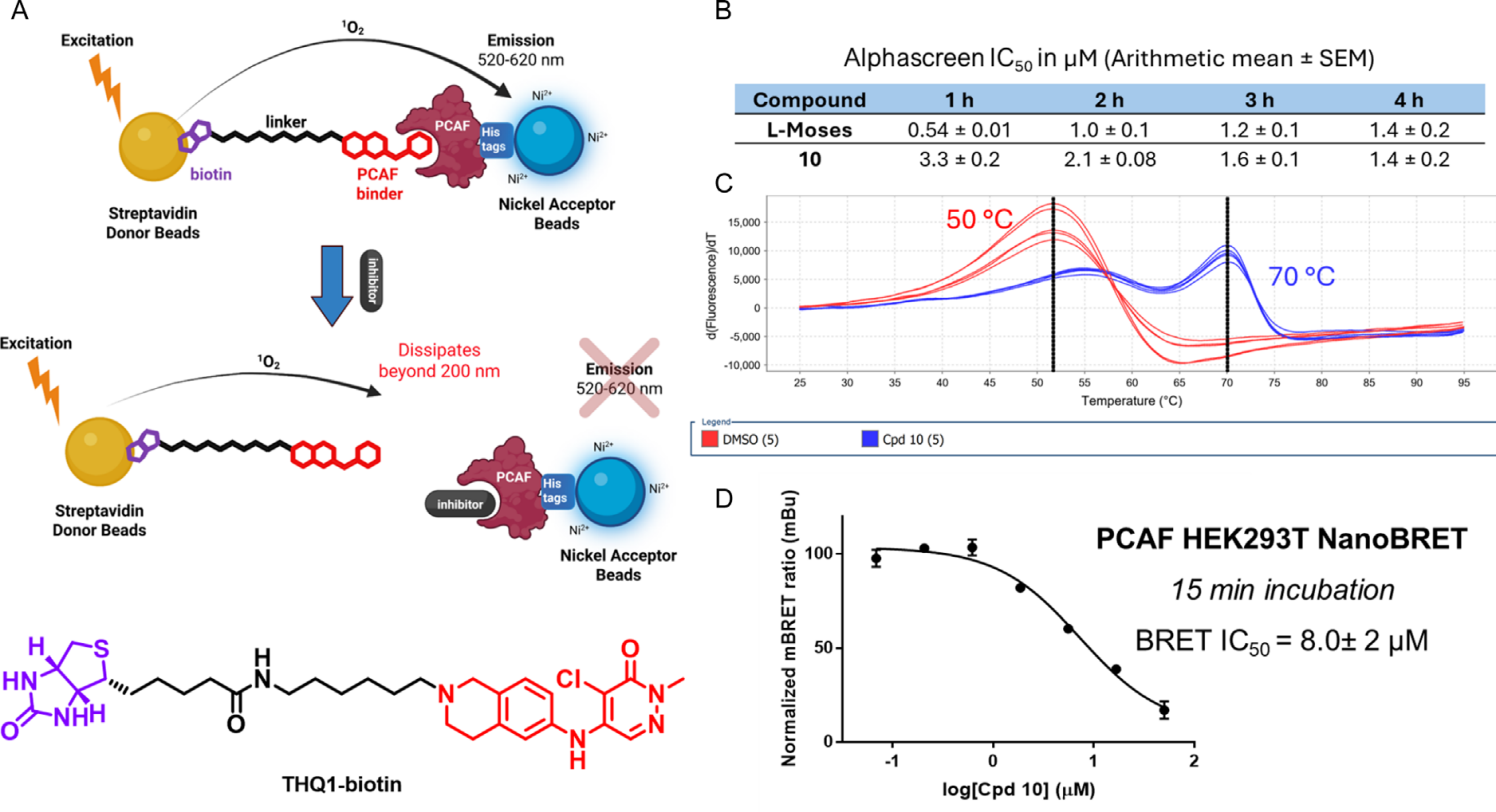
(A) Workflow of Alphascreen competition assay with PCAF probe THQ1‐biotin. (B) Time‐dependent IC_50_ values obtained after treatment of PCAF bromodomain with either L‐Moses or **10** (*n* = 3). Drop in signal likely due to beads getting photobleached with time. (C) DSF data showing stabilization of PCAF bromodomain by **10.** (D) Cell‐based NanoBRET PCAF BRD competition assay with **10** (*n* = 2).

With these results supporting the feasibility of covalently targeting the PCAF bromodomain, we subjected **10** to biophysical assays to assess time dependence, functional effects of inhibition, and in‐cell target engagement. To evaluate the functional effect of inhibiting the PCAF bromodomain with **10** in a time‐dependent manner, we developed an Alphascreen competition experiment using a synthesized biotinylated probe (**THQ1‐biotin**) based on **THQ1** (Figure 6A). Consistent with a covalent mechanism, the IC_50_ of **10** decreased as time progressed, while that of L‐Moses (reported non‐covalent inhibitor of PCAF, *K*_d_ = 126 nM) did not demonstrate a time‐dependent inhibition (Figure 6B).

Subsequently, we tested the effect of covalent inhibition on protein stabilization, as a covalent inhibitor could be either stabilizing or denaturing leading to the observed inhibition. Differential scanning fluorimetry (DSF) was used to evaluate the binding stabilization of PCAF by **10**. Data obtained shows that treatment of PCAF bromodomain with **10** resulted in a highly significant +20°C stabilization (Figure 6C). At initial time‐points, a second population of protein was observed at the intermediate stabilization (Δ*T*_M_ = 55°C), which disappears in a time‐dependent manner (Figure S7). We attribute this second population to the noncovalent complex of **10** and PCAF. Finally, to evaluate the ability of **10** to engage PCAF inside HEK293T cells, we used a NanoBRET target engagement assay with a BODIPY‐tagged PCAF probe [41]. In this assay, compound **10** had a BRET IC_50_ of 8.0 ± 2 μM (Figure 6D), supporting the permeability of **10** for downstream applications. Notably, raw donor emission (which is a proxy for cell toxicity) remains high even at the top concentration of **10.** This implies that the observed IC_50_ of **10** is due to inhibition and not cytotoxicity, as the latter is usually accompanied by very low donor emission at high concentrations of the compound (Figure S11). We also tested **10** in the NanoBRET assay targeting paralog GCN5. A similar IC_50_ of 11 ± 0.4 μM was determined from the NanoBRET assay (Figure S12). Additionally, covalent inhibition of recombinant GCN5 was verified with **10** (Figure S14). Together, these results are consistent with covalent inhibition of both class I bromodomains in cells, setting the stage for future cellular investigations.

## 3 Conclusion

In summary, we have established the unique reactivity of a surface‐exposed lysine for TCI development to generate a covalent inhibitor of the PCAF bromodomain. Analysis of an X‐ray crystal structure revealed the presence of a targetable unique lysine residue. Using the ligand‐first approach, we initially identified a PCAF bromodomain reversible binder (BZ1) and rationally installed lysine‐reactive warheads to develop TCIs for the PCAF bromodomain. This resulted in first‐ and second‐generation TCIs of PCAF, with their covalent inhibition assessed using mass spectrometry, biophysical assays, and cell‐based assays. Initial selectivity assessment showed that a lysine‐reactive electrophile achieved selectivity over a close off‐target class I bromodomain, BPTF. However, CECR2 bromodomain selectivity has yet to be achieved and represents an area for further optimization. Future optimization of **10** or squarate **12** will be a valuable starting point to develop a high affinity bromodomain probe to study the role of PCAF bromodomain in replication of integrated HIV‐1 genome and progression of glioblastoma, and future heterobifunctional molecules based on the PCAF/GCN5 histone acetyltransferase domains.

## Supporting Information

Additional supporting information can be found online in the Supporting Information section.

## Author Contributions

**Richard R.** **Ede:** investigation, conceptualization, writing – original draft, methodology, validation, writing – review and editing, formal analysis, data curation. **Richard K. Begyinah:** data curation, methodology, writing – review and editing. **William C. K. Pomerantz:** supervision, resources, formal analysis, writing – review and editing, writing – original draft, funding acquisition, conceptualization, investigation. **Molly S. Sneddon:** writing – review and editing, methodology, formal analysis, data curation. **Kerstin E. Peterson:** writing – review and editing, methodology, data curation. **Ana Katrina Y. Tiu:** investigation, validation, methodology, formal analysis, data curation. **Anang A. Shelat:** writing – review and editing, project administration, resources, supervision, funding acquisition. **Marcus Fischer:** project administration, resources, supervision, writing – review and editing, funding acquisition. **Irin P. Tom:** methodology, writing – review and editing, data curation. **Jason Ochoada:** writing – review and editing, methodology, validation, formal analysis, data curation.

## Funding

This work was supported by the National Institutes of Health (R01CA290805, M.F, A.A.S, R35GM140837 W.C.K.P, and T32GM132029, M.S). KEP was additionally supported by the NSF GRFP. The American Lebanese Syrian Associated Charities (ALSAC) also supports the research of A.A.S and M.F.

## Conflicts of Interest

The authors declare no conflicts of interest.

## Supporting information

Supplementary Material

## Data Availability

The data that support the findings of this study are available in protein data bank at https://www.rcsb.org/, reference number PDBIDs 7M2E, 6J3O, 7RWQ. These data were derived from the following resources available in the public domain: ‐ PDB, https://www.rcsb.org/.
