# Supplementary material for: Development of a Lysine‐Reactive Targeted Covalent Inhibitor for the P300/CBP‐Associated Factor Bromodomain Through Structure‐Based Design
Source: ChemMedChem. 2026 May 22;21(10):e70301. doi: 10.1002/cmdc.70301 (PMC13206289; doi:10.1002/cmdc.70301)
Supplement: Supplementary file 1 — Supplementary Material [file CMDC-21-e70301-s001.pdf]

SUPPORTING INFORMATION

Development of a Lysine-Reactive Targeted Covalent Inhibitor for the P300/CBP-Associated Factor (PCAF) Bromodomain Through Structure-Based Design

Richard R. Ede, Kerstin E. Peterson, Richard Begyinah, Irin P. Tom, Jason Ochoada, Molly Sneddon, Ana Katrina Y. Tiu, Marcus Fischer, Anang A. Shelat, William C. K. Pomerantz*

WILEY-VCH

Table of Contents

Supplementary figures and tables: 3
General materials and methods 15
Protein expression and purification 15
LC-MS analysis of PCAF BRD modification 15
Determination of k_{inact}/K_I 15
LC-MS/MS analysis of PCAF modification by compounds 5 and 10 16
Alphascreen competition 17
Differential scanning fluorimetry 17
NanoBRET target engagement assay 17
Covalent Docking 17
Compound synthesis and characterization 18
References 27
NMR Spectra 28

WILEY-VCH

Supplementary figures and tables:

Table S1. Covalent modification of PCAF BRD after 24 h incubation with respective molecules determined by intact protein mass spectrometry. Arithmetic mean±SEM (standard error of the mean) shown.

Compound	Number of replicates (n)	%labelling
**1**	3	69±0.6
**2**	2	95±4
**3**	2	0
**4**	2	18±5
**5**	3	33±5
**6**	3	53±0.8
**7**	2	0
**8**	3	0
**9**	2	7±0.4
**10**	3	92±7
**11**	2	47±2
**12**	3	94±0.2

WILEY-VCH

WILEY-VCH

A

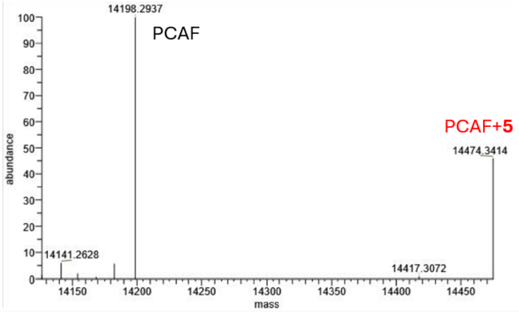

B

Xtract Masses Table					
Monoisotopic Mass	Sum Intensity	Number of Charge States	Average Charge	Delta Mass	Relative Abundance
14198.2937	962464.94	12	16.22	0.0000	100.0000
14474.3414	445563.24	10	16.15	276.0477	46.2940
14141.2628	57836.17	6	17.19	-57.0309	6.0092
14182.2831	56917.66	7	17.26	-16.0106	5.9137
14154.2669	20069.46	4	18.06	-44.0268	2.0852
14126.2434	14445.55	4	18.08	-72.0503	1.5009
14417.3072	12541.84	3	17.85	219.0135	1.3031
14168.2747	9154.94	3	18.61	-30.0190	0.9512

Figure S1. Representative intact MS data obtained from the incubation of PCAF BRD (20 μM) with compound **5 (**30 μM) for 24 h at room temperature.

A

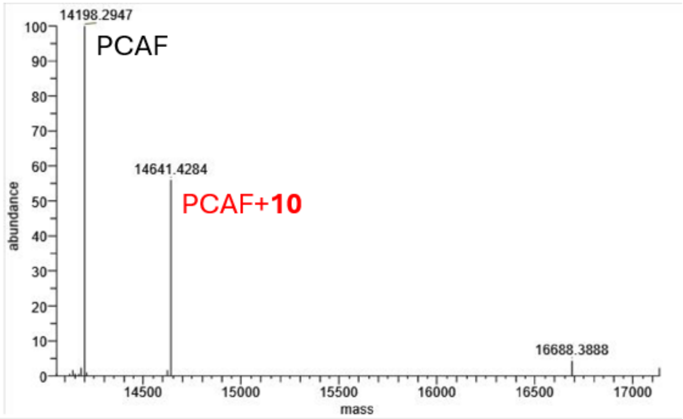

B

Xtract Masses Table					
Monoisotopic Mass	Sum Intensity	Number of Charge States	Average Charge	Delta Mass	Relative Abundance
14198.2947	530611.77	14	16.48	0.0000	100.0000
14641.4284	298873.17	14	16.72	443.1337	56.3261
16688.3888	24571.13	8	21.28	2490.0941	4.6307
17131.4794	13871.56	6	21.87	2933.1847	2.6143
14183.3142	13689.94	4	19.01	-14.9805	2.5800
14141.2586	10150.95	5	18.50	-57.0361	1.9131
14625.3852	9400.72	5	18.74	427.0905	1.7717
14214.3000	6298.53	3	17.94	16.0053	1.1870
14169.2846	4847.55	3	18.79	-29.0101	0.9136
14154.2656	4116.20	3	18.94	-44.0291	0.7757
14125.2402	3860.54	3	18.83	-73.0545	0.7276
14054.2395	3620.33	3	18.86	-144.0552	0.6823

Figure S2. Representative intact MS data obtained from the incubation of PCAF BRD (20 µM) with compound 10 **(**30 µM) for 4 h at room temperature. Notably, there is no evidence of double labelling.

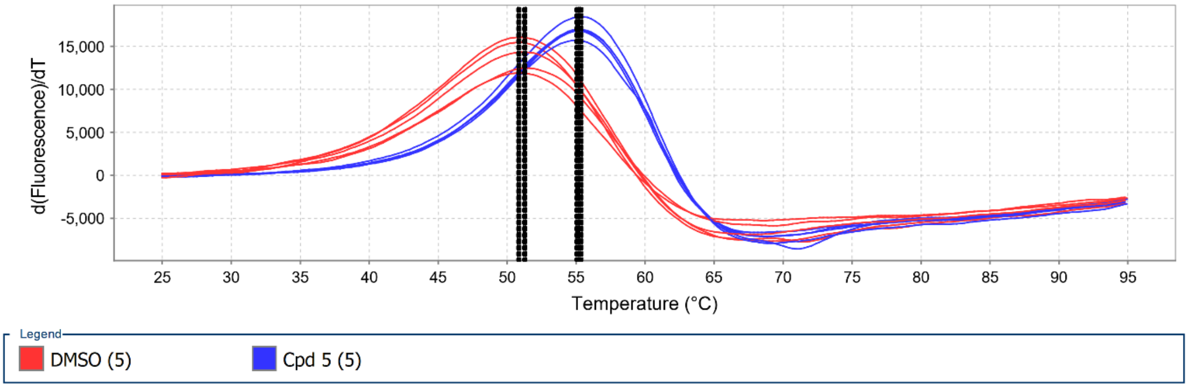

Figure S3. Differential Scanning Fluorimetry (DSF) plots obtained from the pre-incubation of PCAF BRD (15 µM) and compound **5** (75 µM). Red traces correspond to the PCAF BRD alone and blue traces are in the presence of compound **5**. Data shown for 5 technical replicates.

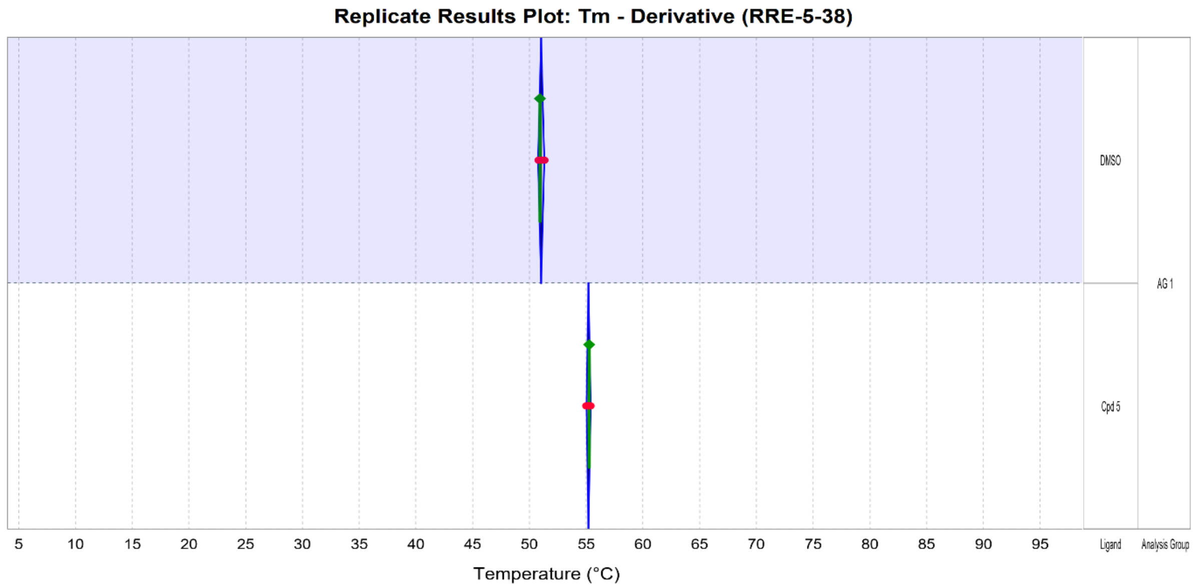

Figure S4. DSF replicate plots from the pre-incubation of PCAF BRD (15 μM) and compound **5** (75 μM). Data shown for 5 technical replicates.

WILEY

Peptide Summary

Sequence: MEPVKRTEAPGYY, K5-phenyl ester (275.04620 Da)

Charge: +2, Monoisotopic m/z: 908.40186 Da (+1.9 mmu/+2.09 ppm), MH+: 1815.79643 Da, RT: 20.4358 min,

Identified with: Sequest HT (v1.17); XCorr:3.21,

Fragment match tolerance used for search: 0.02 Da

Fragments used for search: y-H_2O; y-NH_3; b; b-H_2O; b-NH_3; y

Fragment Matches

Value Type: Theo. Mass [Da]

Ion Series | Neutral Losses | Precursor Ions | Internal Fragments

#1	b^+	b^{2+}	Seq.	y^+	y^{2+}	#2
1	132.04776	66.52752	M			13
2	261.09035	131.04882	E	1684.75216	842.87972	12
3	358.14312	179.57520	P	1555.70956	778.35842	11
4	457.21153	229.10940	V	1458.65680	729.83204	10
5	860.35269	430.67999	K-phenyl e...	1359.58838	680.29783	9
6	1016.45381	508.73054	R	956.44722	478.72725	8
7	1117.50148	559.25438	T	800.34611	400.67669	7
8	1246.54408	623.77568	E	699.29843	350.15285	6
9	1317.58119	659.29423	A	570.25584	285.63156	5
10	1414.63395	707.82062	P	499.21873	250.11300	4
11	1471.65542	736.33135	G	402.16596	201.58662	3
12	1634.71875	817.86301	Y	345.14450	173.07589	2
13			Y	182.08117	91.54422	1

Figure S5. Modified peptide showing unique lys753 modified obtained from chymotrypsin digestion of PCAF-**5** adduct.

WILEY

Peptide Summary

Sequence: MEPVKRTEAPGYY, K5-ArSO2F RRE (442.08660 Da)
Charge: +3, Monoisotopic m/z: 661.61584 Da (-0.02 mmu/-0.03 ppm), MH+: 1982.83298 Da, RT: 37.3948 min,
Identified with: Sequest HT (v1.17); XCorr:3.56,
Fragment match tolerance used for search: 0.02 Da
Fragments used for search: y-H_2O; y-NH_3; b; b-H_2O; b-NH_3; y

Fragment Matches

Value Type: Theo. Mass [Da]

Ion Series | Neutral Losses | Precursor Ions | Internal Fragments

#1	b^+	b^{2+}	b^{3+}	Seq.	y^+	y^{2+}	y^{3+}	#2
1	132.04776	66.52752	44.68744	M				13
2	261.09035	131.04882	87.70164	E	1851.79256	926.39992	617.93570	12
3	358.14312	179.57520	120.05256	P	1722.74996	861.87862	574.92151	11
4	457.21153	229.10940	153.07536	V	1625.69720	813.35224	542.57058	10
5	1027.39309	514.20019	343.13588	K-ArSO2F ...	1526.62878	763.81803	509.54778	9
6	1183.49421	592.25074	395.16959	R	956.44722	478.72725	319.48726	8
7	1284.54188	642.77458	428.85215	T	800.34611	400.67669	267.45355	7
8	1413.58448	707.29588	471.86634	E	699.29843	350.15285	233.77100	6
9	1484.62159	742.81443	495.54538	A	570.25584	285.63156	190.75680	5
10	1581.67435	791.34082	527.89630	P	499.21873	250.11300	167.07776	4
11	1638.69582	819.85155	546.90346	G	402.16596	201.58662	134.72684	3
12	1801.75915	901.38321	601.25790	Y	345.14450	173.07589	115.71968	2
13				Y	182.08117	91.54422	61.36524	1

Figure S6. Modified peptide showing unique lys753 modified obtained from chymotrypsin digestion of PCAF-**10** adduct.

WILEY

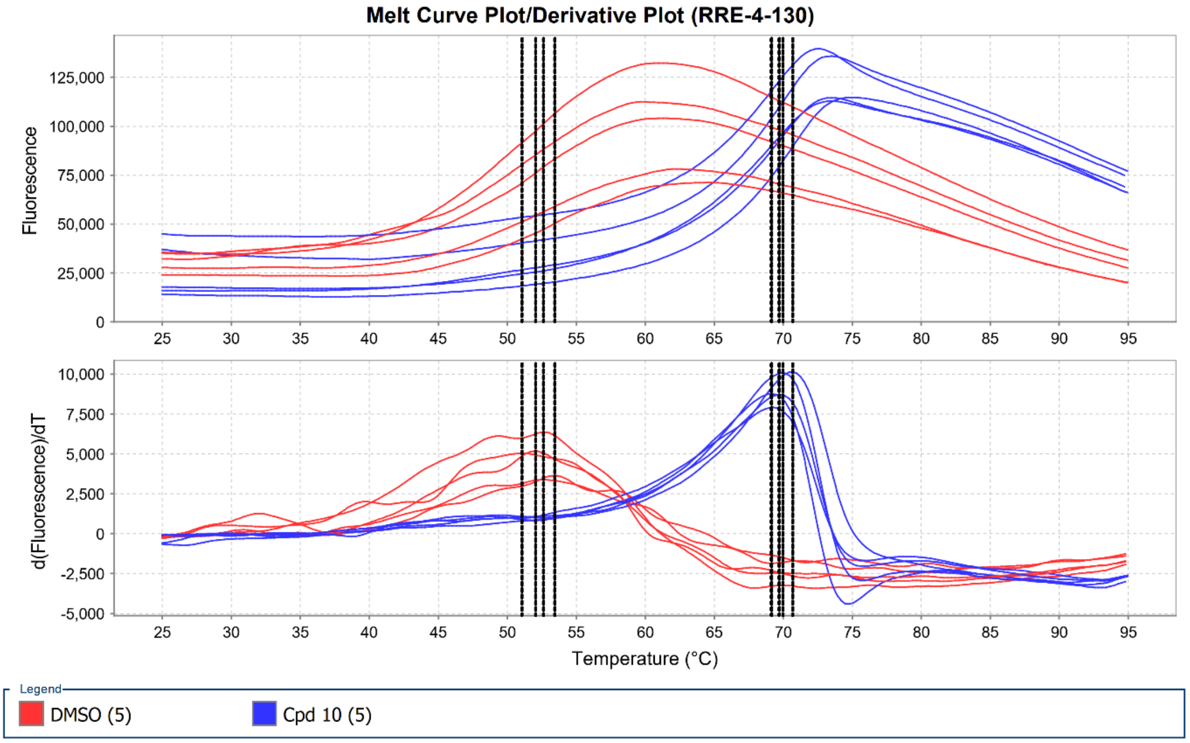

Figure S7. Differential Scanning Fluorimetry (DSF) plots obtained from the pre-incubation of PCAF BRD (15 µM) and compound **10** (75 µM) for 4 h before initiating experiment. Red traces correspond to the PCAF BRD alone and blue traces are in the presence of compound **10**. Data shown for 5 technical replicates.

WILEY-VCH

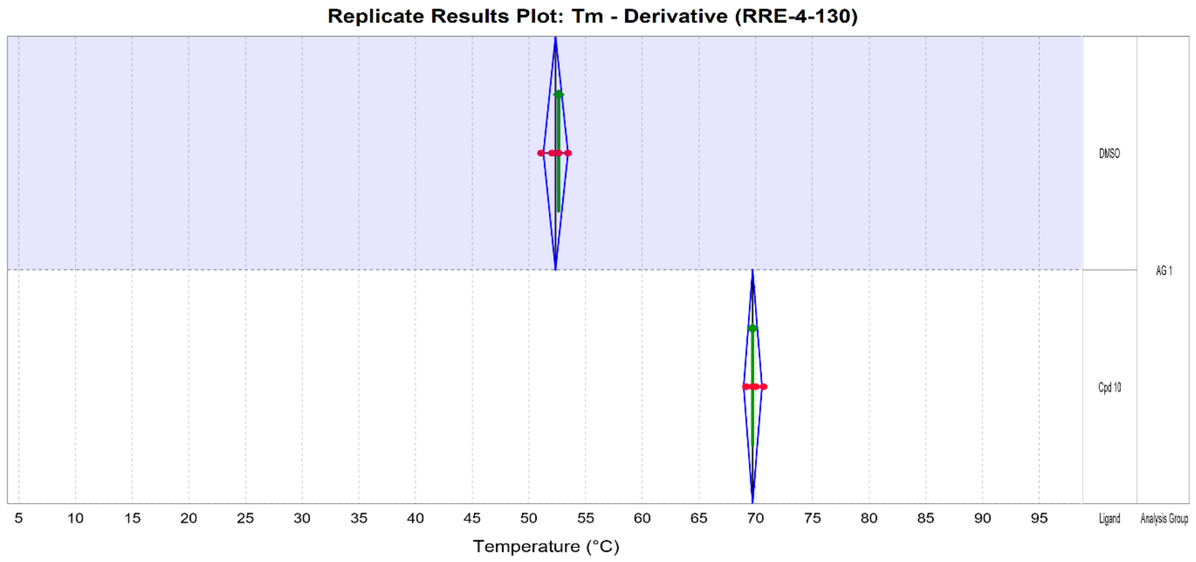

Figure S8. DSF replicates plot from the pre-incubation of PCAF BRD (15 µM) and compound **10** (75 µM) for 4 h before initiating experiment. Data shown for 5 technical replicates.

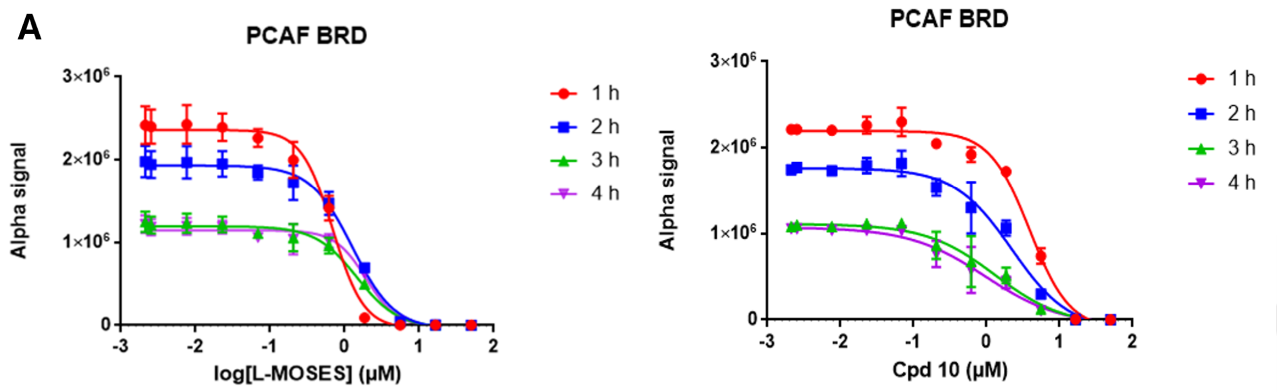

Figure S9. Alphascreen isotherms from incubation of PCAF BRD with either L-Moses or **10**, with IC_{50} values measured after 1 h, 2 h, 3 h, and 4 h related to Figure 5B (means±SEM, n = 3).

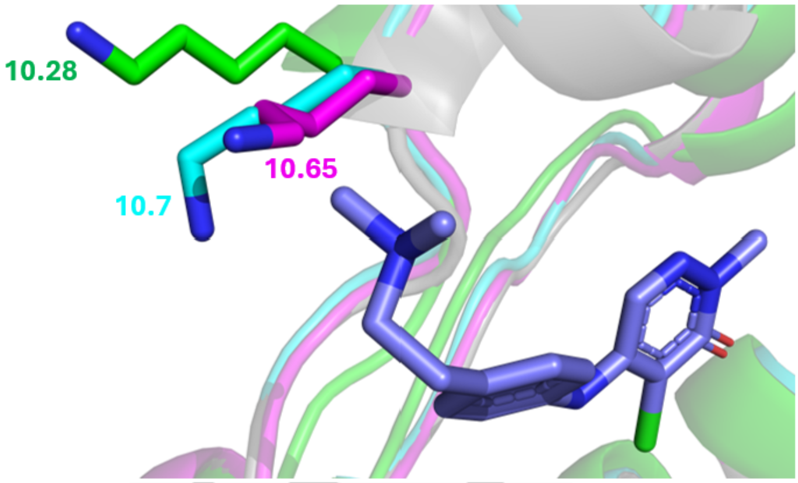

Figure S10. X-ray crystal structure of **Me2BZ1** bound to BPTF BRD (grey, PDB ID 7M2E) overlaid on PCAF BRD (cyan, 6J3O), GCN5 BRD (magenta, 5MLJ), and PB1(1) BRD (green, 3IU5). The equivalent of lysine753 in PCAF is highlighted in GCN5 and PB1(1). The p*Ka* predictions, obtained using the PROPKA function in Schrodinger.

WILEY-VCH

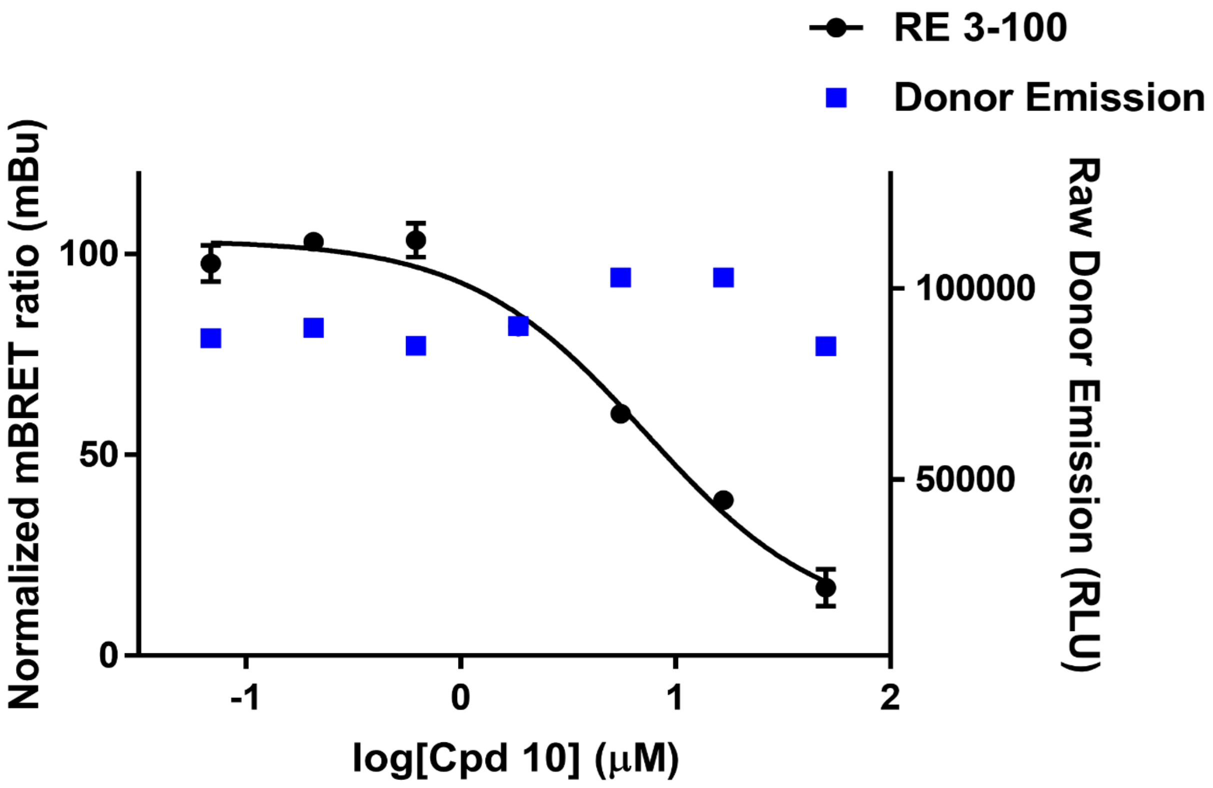

Figure S11. Donor emissioin for the NanoBRET experiment with **10.** No significant drop in donor emission is observed at high concentrations of **10,** which suggests that **10** is actually inhibiting the PCAF bromodomain in cells, rather than being toxic to the cells. The raw donor emission has been plotted as an average of three technical replicates, two experimental replicates (n=2).

WILEY-VCH

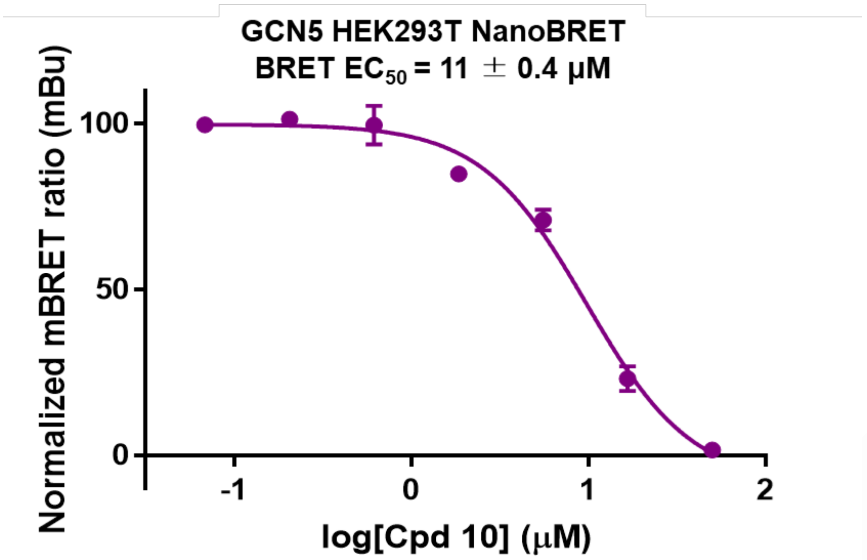

Figure S12. Cell-based NanoBRET competition assay data showing that **10** can outcompete BODIPY probe for binding to the GCN5 bromodomain (n=2).

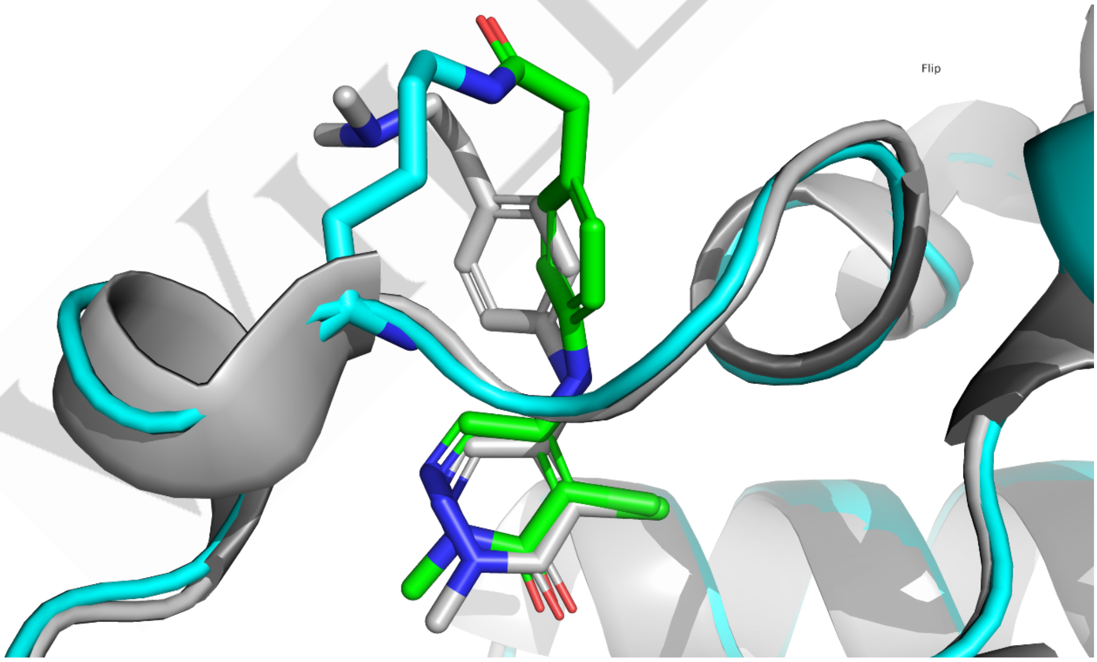

Figure S13. A docked binding pose of cpd **5** covalently reacted with K753 (cyan) overlaid on an X-ray cocrystal structure of BPTF-Me2BZ1 (grey, PDB: 7M2E)

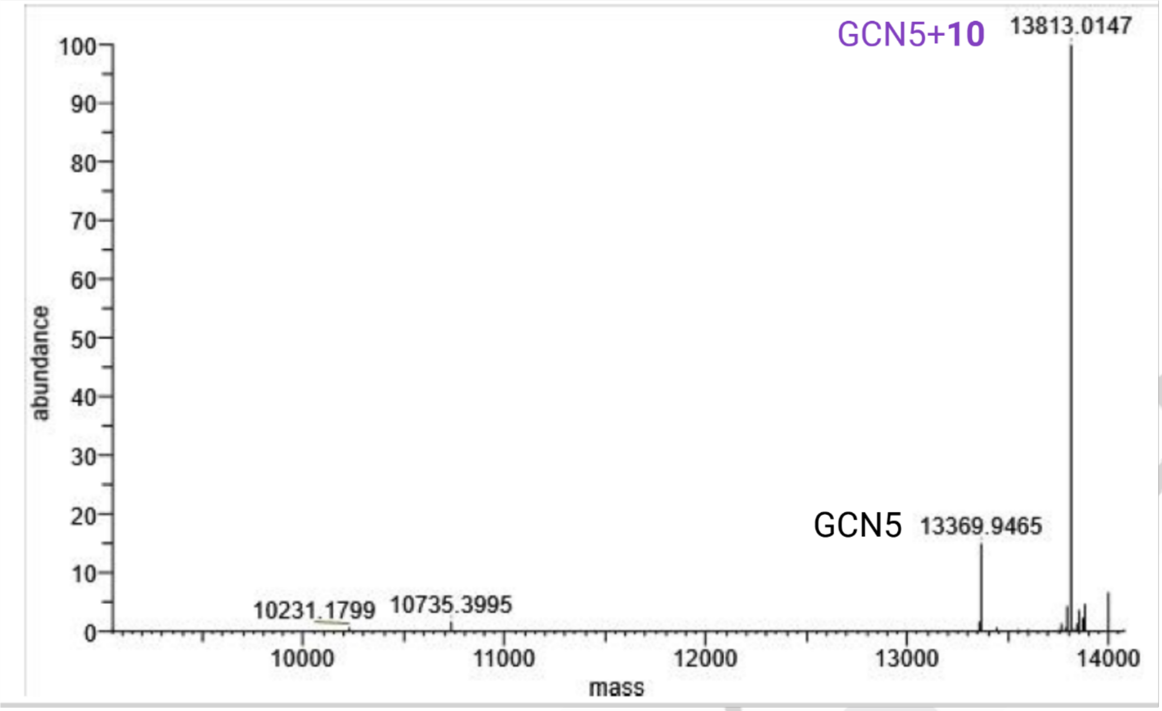

Xtract Masses Table					
Monoisotopic Mass	Sum Intensity	Number of Charge States	Average Charge	Delta Mass	Relative Abundance
13813.0147	30326725.10	14	11.27	0.0000	100.0000
13369.9465	4645240.42	13	11.42	-443.0682	15.3173
13996.0369	2074370.79	11	11.55	183.0222	6.8401
13883.9816	1421624.38	10	10.20	70.9669	4.6877
13796.0127	1351620.14	11	11.30	-17.0020	4.4569
13853.9786	1158776.86	10	10.23	40.9639	3.8210
13867.9684	787621.09	10	10.32	54.9537	2.5971
13352.8879	531777.27	7	9.78	-460.1268	1.7535
10735.3995	525022.93	5	11.26	-3077.6152	1.7312
13767.9886	459838.03	6	11.32	-45.0261	1.5163
13839.9743	416302.14	7	10.46	26.9596	1.3727
13782.0061	274921.96	7	11.70	-31.0086	0.9065

Figure S14. Representative intact MS data obtained from the incubation of GCN5 BRD with compound **10 (**GCN5:10 = 1:1.5) for 24 h at room temperature. Representative data is one of three technical replicates.

General materials and methods

Protein expression and purification

Expression and purification of PCAF BRD:

Protein expression for PCAF BRD was performed in line with previous report.[1] PCAF BRD construct was cloned onto a pET-28a(+)-TEV vector encoding for residues 715-831 from the human PCAF BRD (NCBI Accession: NP_003875.3, UniProt entry number: Q92831) with an N-terminal His9 tag followed by a TEV cleavage site (Genscript). BL21 Star (DE3) E. coli cells were transformed with the PCAF BRD plasmid onto kanamycin agar plates. The incubated were incubated at 37 °C for 18 h. Of the resulting colonies, one colony was then selected for each 5 mL primary culture tube containing kanamycin (50 *µ*g/mL) in Luria Broth (LB) media. These primary cultures were incubated were shaken at 250 rpm at 37 °C for 18 h. Afterwards, the primary cultures were used to inoculate 1 L of LB medium with kanamycin (50 *µ*g/mL). This secondary culture was incubated at 37 °C with shaking until the cells reached an optical density (OD) at 600 nm of 0.6-0.8. The cells were induced by addition of 1mM isopropyl β-D-thiogalactoside (IPTG) at 20 °C overnight, with the resulting cells pelleted via ultracentrifugation. The resulting cells cell pellet was resuspended in 30 mL lysis buffer (50 mM phosphate, 300 mM NaCl, pH 7.4) containing 5 mM PMSF, lysed via sonication, centrifuged at 10,000*g* for 30 min at 4 °C, filtered before the supernatant was applied to a HisTrap FF 5 mL column (Cytiva). Buffer A: Wash (50 mM phosphate, 100 mM NaCl, 30 mM imidazole, pH 7.4) and Buffer B: Elution (50 mM phosphate, 400 mM imidazole, pH 7.4) with gradient as follows: 0% B for 5 Column Volume (CV), then 0-100% B for 20 CV, and finally 100% B for 5 CV. The purified protein was then buffer exchanged into 50 mM HEPES, 100 mM NaCl, pH 7.4 and stored at -80 °C after being flash frozen. For instances where the His-tag was not needed, TEV was used to cleave tag from the protein before flash freezing and storing in the -80 °C.

Expression and purification of GCN5 BRD:

A pET28a plasmid containing the bromodomain sequence of human GCN5 was obtained from GenScript and transformed into E. coli BL21 (DE3) cells. Cells were grown in 2XYT medium until an OD600 of 0.6-0.8 was reached. Protein expression was induced with 1 mM IPTG at 18°C. After 16 hours of overexpression, the cells were collected by centrifugation. The purification protocol was adapted from the previous report by Huang et al.[2] Briefly, the cell pellets were resuspended in buffer containing 50 mM HEPES pH 7.5, 500 mM NaCl, 5 mM Imidazole, 0.5 mM TCEP, 5% glycerol and SIGMAFASTTM Protease inhibitor cocktail (Sigma Aldrich, #S8830). The cells were lysed using sonication and the supernatant was collected by centrifugation at 30,000 rpm for 1 hour. His6-tag containing protein was purified by Nickel affinity chromatography. Protein fractions were pooled and dialyzed with TEV protease overnight and re-applied to a Nickel column to purify the tag-cleaved GCN5 bromodomain. The protein was further purified by Superdex-75 size-exclusion chromatography with a buffer containing 20 mM HEPES pH 7.5, 100 mM NaCl and 0.5 mM TCEP. The protein eluted as single peak, and the pooled fractions were concentrated to 14.57 mg/ml. The protein was aliquoted and stored at -80°C.

LC-MS analysis of PCAF BRD modification

PCAF BRD was treated with compounds **1** – **11** (1:1.5 ratio of protein:inhibitor) in 50 mM HEPES, 100 mM NaCl, pH 7.4 and incubated at room temperature for 24 h (1-10% DMSO). For selectivity assessment involving BPTF, BRD4 D1, and BRD9 the respective proteins were incubated with either cpd **5** or cpd **10** under same conditions. After incubation, samples were quenched with an equal volume of 1% acetic acid, buffer exchanged into 10 mM ammonium acetate using Millipore Sigma Amicon Ultra centrifugal filters with 10K molecular weight cutoff (part number UFC501096). Samples were then analyzed via intact-protein LC-MS further described below. Experiments were performed at least twice.

Determination of k_{inact}/K_I

For obtaining the values of K_I and k_{inact}, 20 µM of PCAF BRD in 50 mM HEPES, 100 mM NaCl, pH 7.4 was treated with either **5** or **10** (final concentration 20-640 µM) at room temperature with a final volume of 120 µL for each sample (1% DMSO). For **5**, incubation times ranged from 0 – 44 hours while for **10**, incubation times ranged from 0 – 4 hours. At each time point, a 20 µL aliquot was taken out, quenched with an equal volume of 1% acetic acid, flash frozen and stored at -80 °C. Before LC-MS analysis, samples were buffer exchanged into 10 mM ammonium acetate as described above. The LC-MS runs were performed as follows:

All intact protein LC-MS analysis were performed at the Analytical Biochemistry Core Facility at the University of Minnesota's Masonic Cancer Centre. The specific instrument used for this is a Thermo Scientific Orbitrap Elite LC-MS (Waltham, MA) with an UltiMate 3000 RSLCNano UPLC (Thermo Scientific) with positive electrospray ionization. For the LC component, Chromatography was performed using Agilent (Santa Clara, CA) ZORBAX RRHD C3 column 300Å, 2.1 x 100 mm, 1.8 µm. For the mobile phases consisted of buffer A (0.1% formic acid in H_2O) and buffer B (0.1% formic acid in CH_3CN) with the following gradient profile: 0.00-1.0 min for loading, 20% B, flowrate 300.0 µL/min; 1.0-3.0 min, 20-100% B, 300.0 µL/min; 3.0-5.0 min, 100% B, 300.0 µL/min; 5.0-5.12 min, 100-20% B, 300.0µL/min. The resulting raw files were processed using Protein Deconvolution 2.0.54.2 SP2 (Thermo Scientific) with the Manual Xtract, isotopically resolved experiment type, giving the ion counts observed for the most abundant species.

For time-dependent labeling while evaluating kinetic parameters, the %Labelling (%of PCAF modified) of the compounds was determined using the following formula:

$$\%\ \text{Covalent Modification} = \frac{\text{abundance of labelled}}{\text{abundance of unlabelled} + \text{abundance of labelled}} \times 100 \qquad (1)$$

The %labelling of labeled PCAF was plotted as a function of time (experimental duplicates) and fit to a single phase exponential association curve (restricted to a plateau of 100% and an initial value of 0%) to obtain k_{obs}, which was plotted against the concentration of the TCI, and then fit to the Michaelis-Menten equation to yield the values of K_I and k_{inact} (See equations 2 and 3) with GraphPad Prism 7. Experiments were performed twice (experimental duplicates).[2] It is worth noting that at the lower concentrations of the inhibitor, protein concentration is no longer negligible compared to the concentration of the inhibitor.

$$k_{\text{obs}} = \frac{k_{\text{inact}}[\text{I}]}{\text{K}_\text{I} + [\text{I}]} \qquad (2)$$

$$\text{V}_\text{o} = \frac{\text{V}_{\text{max}}[\text{S}]}{\text{K}_\text{m} + [\text{S}]} \qquad (3)$$

LC-MS/MS analysis of PCAF modification by compounds 5 and 10

WT PCAF BRD in 50 mM HEPES, 100 mM NaCl, pH 7.4 was treated with either compound **5** or **10** (protein:TCI = 1:2) and incubated for 24 h at room temperature. Ammonium bicarbonate was then added to give a final concentration of 50 mM. The sample was then reduced using 30 mM Tris-2-carboxyethyl phosphine hydrochloride (TCEP) followed by incubation for with shaking for 5 minutes at 40 °C. Resulting sample was then alkylated by adding S-Methyl methanethiosulfonate (MMTS) to a final concentration of 10 mM an incubating at room temperature for 10 minutes. Next, 2.5 µL of sequencing grade chymotrypsin (1 µg/µL, Promega) was added and pH adjusted to 8 by addition of ammonium bicarbonate. The mixture was then incubated for 18 h at 37 °C. Afterwards, samples were allowed to cool down to room temperature and digestion was quenched by adding a solution of 2.5% trifluoroacetic acid (TFA) until a pH <4. The resulting peptides were desalted with C18 OMIX ziptips (Agilent) following the manufacturers protocol (80% acetonitrile/0.1% TFA elution), dried, and resuspended in 0.1% formic acid. These peptides were then analyzed by LC-MS/MS at the Analytical Biochemistry Core Facility at the University of Minnesota's Masonic Cancer Centre. The instrument utilized is a hybrid high field Orbitrap mass spectrometer (Fusion, Thermo Scientific, Waltham, MA) with an HPLC (Ultimate 3000, Thermo Scientific, Waltham, MA) equipped with a reverse-phase C18 column (4.6 × 250 mm, 100 Å, 5µm Luna Phenomenex, Torrance, CA) operating in positive nanospray ionization mode at a voltage of 2.1 kV. The ion transfer tube temperature was set to 305°C and the RF Lens setting was maintained at 60%. Fragmentation was done by CID. Master Scan survey scans were performed with a cycle time of 2.5 seconds. Orbitrap resolution was set at 60,000 with a scan range of 375-1600 (m/z). Peptides were eluted with buffer A (0.1% formic acid in H_2O) and buffer B (0.1% formic acid in CH_3CN) with the following gradient: 0-5.5 min for loading, 2% B, flowrate 1.0 µL/min; 5.5-26 min, 2% B, 0.3 µL/min; 26-26.5 min, 2-40% B, 0.3 µL/min; 26.5-27 min, 40-95% B, 0.3 µL/min; 27-30.2 min, 95% B, 1.0 µL/min; 30.2-33 min 95-2% B, 1.0 µL/min. The resulting raw files were analyzed using Proteome Discoverer 3.0.1.27 (Thermo Scientific) for chromatogram processing and fragment spectra isolation; Digestion enzyme was set to chymotrypsin with a maximum of 2 missed cleavages. Constant modification was set to methyl disulfide for MMTS; variable

WILEY-VCH

modifications were set to oxidation (M), acetyl (protein N-term), and compound **5** (275.0462 Da) or **10** addition (442.0866 Da); FDR was set to 5% at the protein level and 1% at the peptide level.

Alphascreen competition

Samples were prepared in a buffer consisting of 50 mM HEPES, 100 mM NaCl, 0.1% BSA, 0.05% CHAPS, pH 7.4. Serial dilutions were made by varying concentration of ligand, with concentration of the protein (His9-tagged PCAF BRD) kept constant at 20 nM (0.25 % DMSO). 5 µl of these solutions were then added to a 384-well white plate, to which 5 uL of Alphascreen probe, THQ1-biotin, was added to a final probe concentration of 200 nM. 10 µL of a combined solution of Alphascreen streptavidin donor and nickel chelate acceptor beads (6760619M, Perkin Elmer) were added to furnish a final concentration of 40 µg/mL and allowed to incubate for 1 h, 2 h, 3 h, and 4 h at room temperature in the dark. Subsequently, emission was measured on a Tecan Spark reader using Alphascreen filter settings. Data obtained was polled in GraphPad Prism 7 using a sigmoidal 4-parameter logistic (4PL) curve fit.

Differential scanning fluorimetry

The thermal melting points were assessed using an Mx3000 PCR Machine (QuantStudio 5 384-Well Block). To a 384-well plate was added PCAF BRD solution (buffered in 50 mM HEPES, 100 mM NaCl, at pH 7.4), an equal volume of either DMSO, L-Moses, or compound **10** to a final concentration of 75 µM for each compound (2.5% DMSO). PCAF BRD concentration was maintained at 15 µM. SYPRO Orange dye (Prod. # S5692, 1000x) was used as a fluorescent probe (final concentration of 2x). The final volume of liquid in each well was 20 µL. After addition of all reagents, the plate was then sealed and read, with temperature increasing from 25 ^{o}C and 95 ^{o}C at a rate of 0.5 ^{o}C per minute. Data was visualized using GraphPad Prism.

NanoBRET target engagement assay

BRET assays were performed according to the Promega NanoBRET TE 590 Dyes TM 697 manufacturer protocol using white, 384-well tissue-culture-treated plates (Corning #3570). HEK293T cells were plated at 7 x 10^3 cells per well. PCAF with C-terminal Nanoluciferase (Nluc) and N-terminal Nluc-GCN5 constructs were used (Promega) with a concentration of 1 µM fluorescent tracer MS-1. Stock DMSO solutions of inhibitors were diluted to a 1,000X concentration in 100% DMSO and subsequently dissolved to a final 10X concentration in Opti-MEM. Inhibitor was added to each well, then a 3X stock solution of NanoBRET Nano-Glo Substrate and Extracellular NanoLuc Inhibitor in Opti-MEM was dispensed into each well. BRET was then immediately measured with a Tecan Spark plate reader following a 30 second orbital shake with an amplitude of 1 mm and frequency of 510 rpm. Luminescence was measured using the luminescence multi color setting with a donor emission of 445 nm – 470 nm and acceptor emission of 610 nm – 700 nm using an integration time of 300 ms. A BRET ratio for each concentration was calculated as acceptor response divided by donor response. The BRET ratio was then subtracted by the no tracer DMSO control well ratio and multiplied by 1000 to obtain a milliBRET (mBRET) value. The mBRET values were plotted and fit using the 4-parameter dose-response variable slope curve in GraphPad Prism.

Covalent Docking

Schrödinger Maestro molecular modeling suite (Schrödinger Release 2025-3: LigPrep, Glide, Covalent Docking; Schrödinger, LLC, New York, NY, 2025) was used for all molecular modeling work. A fragment bound to the PCAF bromodomain was obtained from the Protein Data Bank (PDB code: 5MKX, as it contained an identical fragment to those in our molecules of interest.[3] Waters were removed if more than 5 Å from the ligand or if they had fewer than three hydrogen bonds to non-water atoms. Hydrogen-bond optimization and restrained minimization of the complex (converging heavy atoms to an RMSD of 0.3 Å) were completed to provide the starting structure for modeling.

10 and **5** were docked into 5MKX using the Schrödinger Glide Standard Precision (SP) method to confirm acceptable conformations that positioned the sulfonyl fluoride warhead near Lys753.[4] After confirmation, initial attempts to covalently dock the ligands were unsuccessful.[5] This was due to inadequate flexibility of the target lysine residue in the covalent docking program. To address this, the dihedral angle between C4 and C5 of Lys753 was rotated by 60 degrees to the alternate linear form. Subsequent covalent docking was successful and yielded the expected products.

Compound synthesis and characterization

Unless otherwise stated, all NMR spectra were recorded on a Bruker Avance III AX-400. Chemical shifts were reported as parts per million (ppm) from solvent references. Coupling constants (J) are in Hz. High resolution ESI-MS spectra were recorded on a SCIEX X500R QTOF system. All commercially available reagents were used without further purification. Flash column chromatography was performed on a Teledyne-Isco Rf-plus CombiFlash instrument with RediSep columns.

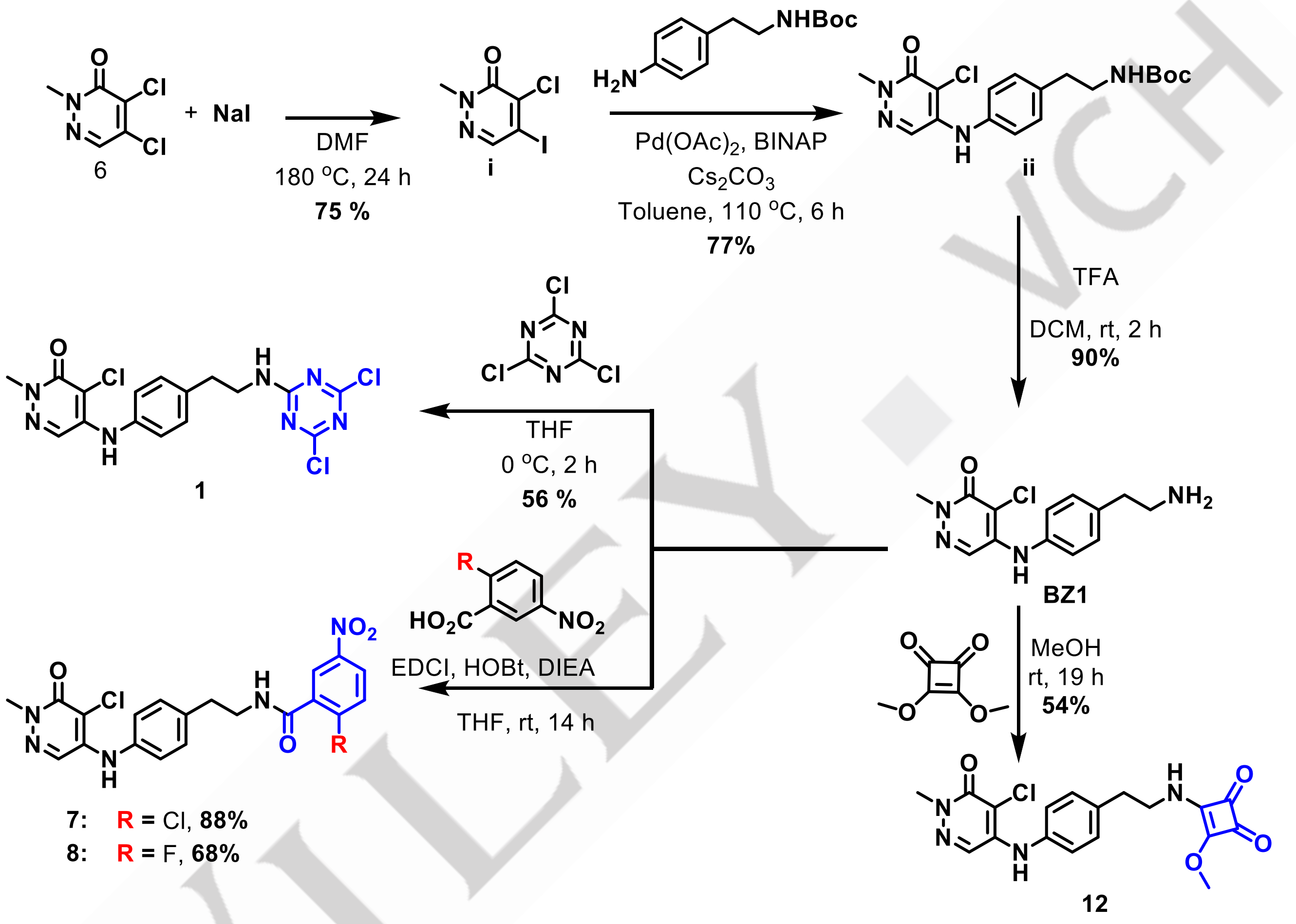

Scheme S1. Chemical synthesis of compounds **1, 7, 8**, and **12**

General Procedure A for synthesis of ii, compound iii, and compound v:

Two oven-dried round bottom flasks were capped and filled with nitrogen. Both flasks were then purged three times with nitrogen. To one of the flasks (A) was added BINAP (0.09 equiv), $Pd(OAc)_2$ (0.06 equiv), and toluene (0.2 M) while to the other flask (A) was added the aryl halide (1.0 equiv), the amine (1.5 equiv), and toluene (0.2 M). Once the aryl halide and amine were fully dissolved, contents of flask B were then transferred to flask A and purged. Cesium carbonate (2.4 equiv) was then added, and the mixture was purged. The reaction was stirred under nitrogen for 6 h at 110 °C.[6,7] Following completion of the reaction, the mixture was filtered through a silica gel plug to remove left-over catalyst, concentrated in vacuo, and purified by flash column chromatography (CombiFlash Rf system, hexanes/ethyl acetate, 0-100% ethyl acetate) to obtain the final product.

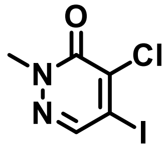

Synthesis of 4-chloro-5-iodo-2-methylpyridazin-3(*2H*)-one (compound i): Synthesis adapted from Dunkel at al.[8] To a round bottom flask containing dimethyl formamide (354 mL, 0.5 M) was added 4,5-dichloro-2-methylpyridazin-3(*2H*)-one (31.7 g, 177 mmol) and sodium iodide (79.65 g, 530 mmol). The reaction mixture was refluxed at 180 °C for 24 h. After completion of the reaction, resulting mixture was washed with a solution of 8 M lithium chloride (x3), extracted into ethyl acetate (x3), dried using anhydrous magnesium sulfate and filtered. The crude reaction mixture was then concentrated in vacuo to remove excess solvent. Recrystallization was then performed using methanol to obtain compound **I** as a white solid. No purification was performed.

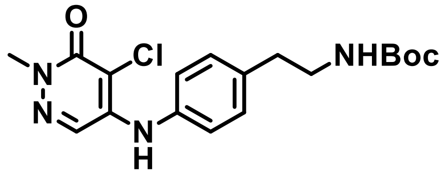

Synthesis of tert-butyl (4-((5-chloro-1-methyl-6-oxo-1,6-dihydropyridazin-4-yl)amino)phenethyl)carbamate (compound ii): Following General procedure A, tert-butyl (4-aminophenethyl)carbamate (1.3 g, 5.55 mmol, 1.5 equiv), compound **i** (1g, 3.70 mmol, 1.0 equiv), Pd(OAc)$_2$ (49 mg, 0,22 mmol, 0.06 equiv), BINAP (205 mg, 0.33 mmol, 0.09 equiv), Cesium carbonate (2.9 g, 8.9 mmol, 2.4 equiv) were reacted. Compound **ii** was obtained as a brown solid (1.09 g, 2.9 mmol, 77%). ^{1}H NMR (400 MHz, CDCl$_3$) δ 7.64 (s, 1H), 7.24 (d, *J* = 8.3 Hz, 2H), 7.13 (d, *J* = 8.3 Hz, 2H), 6.38 (s, 1H), 4.59 (s, 1H), 3.76 (s, 3H), 3.38 (q, *J* = 6.6 Hz, 2H), 2.82 (t, *J* = 7.2 Hz, 2H), 1.44 (s, 9H). HRMS (ESI) calculated for $C_{18}H_{23}ClN_4O_3$ [M+H]$^+$: 379.1531, found 379.1519. Spectral characterization for this molecule was consistent with previous report.[9]

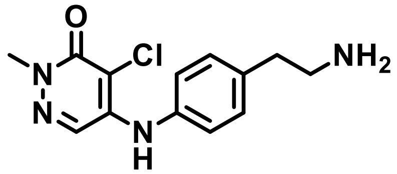

Synthesis of 5-((4-(2-aminoethyl)phenyl)amino)-4-chloro-2-methylpyridazin-3(2H)-one (BZ1): The purified compound **ii** (1.09 g, 2.9 mmol, 1 equiv) was then dissolved in DCM (1-3 mL) at room temperature. To the mixture was added trifluoroacetic acid (5.0 equiv), and the resulting mixture was stirred at room temperature overnight. DCM was then removed under a stream of nitrogen. To obtain **BZ1** as a free base, the mixture was extracted into DCM and then adjusted to a pH > 10 using a solution of 1 M NaOH. The organic layer was then separated and dried using anhydrous magnesium sulfate, filtered, and DCM removed in vacuo to furnish the product. The crude product was used for the next step without further purification.

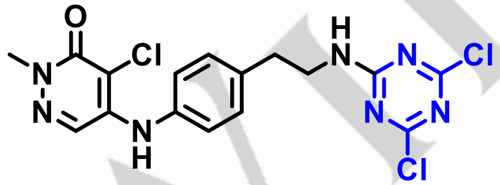

Synthesis of 4-chloro-5-((4-(2-((4,6-dichloro-1,3,5-triazin-2-yl)amino)ethyl)phenyl)amino)-2-methylpyridazin-3(2H)-one (compound 1): Procedure adapted from Shannon et al.[10] A solution of 2,4,6-trichloro-1,3,5-triazine (84 mg, 0.46 mmol, 1.28 equiv) in anhydrous THF (4 mL) was cooled to 0 °C. In a separate vial, **BZ1** (100 mg, 0.36 mmol, 1.0 equiv) was suspended in anhydrous THF (2.5 mL) and DIEA (0.13 mL, 0.72 mmol, 2.0 equiv) was added. This resulting suspension of **BZ1** was then added dropwise to the chilled triazine solution over the course of 1 h. The resulting mixture was then stirred at 0 °C for 2 h. Upon completion of the reaction, the solvent was removed in vacuo. The product was then purified using flash column chromatography (CombiFlash Rf system, hexanes/ethyl acetate, 0-100% ethyl acetate) to obtain compound **1** as a white solid (85.5 mg, 0.2 mmol, 56%). ^{1}H NMR (400 MHz, DMSO) δ 9.19 (t, 1H), 8.67 (s, 1H), 7.58 (s, 1H), 7.26 (d, *J* = 8.6 Hz, 2H), 7.17 (d, *J* = 8.6 Hz, 2H), 3.60 (s, 3H), 3.54 (q, *J* = 6.5 Hz, 2H), 2.83 (t, *J* = 7.2 Hz, 2H). ^{13}C NMR (101 MHz, DMSO) δ 207.04, 206.05, 202.87, 194.65, 180.11, 174.31, 173.17, 167.34, 165.10, 161.42, 145.73, 86.23, 79.78, 71.18. HRMS (ESI) calculated for $C_{16}H_{14}Cl_3N_7O$ [M+H]$^+$: 426.0398, found 426.0372.

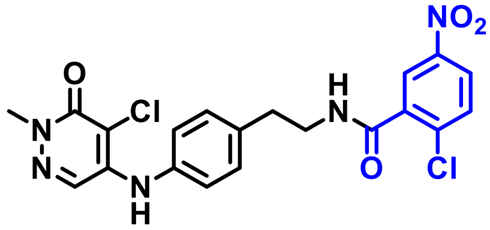

Synthesis of 2-chloro-N-(4-((5-chloro-1-methyl-6-oxo-1,6-dihydropyridazin-4-yl)amino)phenethyl)-5-nitrobenzamide (compound 7): Procedure adapted from Shannon et al.[10] In a vial (flask 1) the 2-chloro-5-nitrobenzoic acid (32 mg, 0.16 mmol, 1.0 equiv) was dissolved in acetonitrile (4 mL). HOBT (35 mg, 0.26 mmol, 1.6 equiv) and EDCI (79 mg, 0.26 mmol, 1.6 equiv). The mixture in flask 1 was stirred at room temperature for 1 hour. In a separate vial (flask 2), **BZ1** (70 mg, 0.25 mmol, 1.6 equiv) and DIEA (0.12 mL, 0.67 mmol, 4.2 equiv) were dissolved in THF (5 mL). The activated acid solution in flask 1 was then added to flask 2 and stirred for 3 hours. Following completion of the reaction, the crude product was concentrated in vacuo, washed with distilled water (x3), extracted into ethyl acetate (x3), dried using anhydrous magnesium sulfate, and purified using flash column chromatography (CombiFlash Rf system, hexanes/ethyl acetate, 0-100% ethyl acetate) to obtain compound **7** as a white solid (66 mg, 0.14 mmol, 88%). ^{1}H NMR (400 MHz, DMSO) δ 8.76 (t, *J* = 5.6 Hz, 1H), 8.69 (s, 1H), 8.26 (dd, *J* = 8.8, 2.8 Hz, 1H), 8.15 (d, *J* = 2.8 Hz, 1H), 7.80 (d, *J* = 8.8 Hz, 1H), 7.58 (s, 1H), 7.31 (d, *J* = 8.4 Hz, 2H), 7.20 (d, *J* = 8.4 Hz, 2H), 3.60 (s, 3H), 3.53 (q, *J* = 7.3 Hz, 2H), 2.87 (t, *J* = 7.2 Hz, 2H). ^{13}C NMR (101 MHz, DMSO) δ 164.22, 157.02, 145.94, 142.54, 137.82, 137.04, 136.57, 136.12, 131.30, 129.73, 127.44, 125.35, 123.84, 123.61, 108.01, 40.45, 39.59, 34.14. HRMS (ESI) calculated for $C_{20}H_{17}Cl_2N_5O_4$ [M+H]$^+$: 462.0730, found 462.0694.

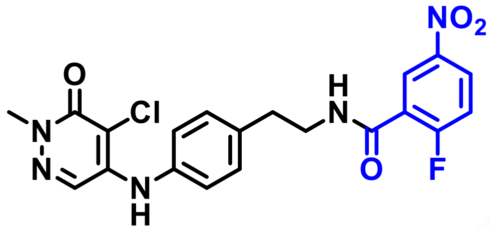

Synthesis of N-(4-((5-chloro-1-methyl-6-oxo-1,6-dihydropyridazin-4-yl)amino)phenethyl)-2-fluoro-5-nitrobenzamide (compound 8): Procedure adapted from Shannon et al.[10] The 2-fluoro-5-nitrobenzoic acid (41 mg, 0.22 mmol, 1.0 equiv) was dissolved in THF (0.15 M). The solution was stirred until all components had dissolved fully. Afterwards **BZ1** (70 mg, 0.22 mmol, 1.0 equiv), HOBT (18 mg, 0.13 mmol, 0.6 equiv), was added DIEA (0.08 mL, 0.44 mmol, 1.0 equiv), and EDCI (85 mg, 0.28 mmol, 1.3 equiv). The mixture was then stirred at room temperature for 4 hours. After completion of the reaction, the THF was removed in vacuo. The crude product was then dissolved in ethyl acetate, washed with 1M HCl (x2), distilled water, saturated sodium bicarbonate (x2), and saturated brine. Resulting solution was then dried using anhydrous magnesium sulfate and purified using flash column chromatography (CombiFlash Rf system, hexanes/ethyl acetate, 0-100% ethyl acetate) to obtain compound **8** as a white solid (67 mg, 0.15 mmol, 68%). ^{1}H NMR (400 MHz, $CDCl_3$) δ 8.96 – 8.90 (m, 1H), 8.38 – 8.29 (m, 1H), 7.63 (s, 1H), 7.30 (d, *J* = 7.9 Hz, 3H), 7.16 (d, *J* = 8.3 Hz, 2H), 6.85 – 6.77 (m, 1H), 6.44 (s, 1H), 3.79 (q, 2H), 3.74 (s, 3H), 2.98 (t, *J* = 7.0 Hz, 2H). ^{13}C NMR (101 MHz, $CDCl_3$) δ 164.67, 162.11, 161.06, 157.90, 142.18, 136.91, 136.15, 130.39, 128.47 (d, *J* = 11.4 Hz), 128.34 (d, *J* = 4.4 Hz), 126.70, 124.52, 124.46, 117.69 (d, *J* = 27.5 Hz), 41.56, 41.44, 40.39, 35.08. ^{19}F NMR (376 MHz, $CDCl_3$) δ -104.72. HRMS (ESI) calculated for $C_{20}H_{17}ClFN_5O_4$ [M+H]$^+$: 446.1026, found 446.0983.

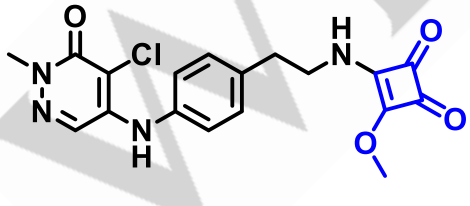

Synthesis of 3-((4-((5-chloro-1-methyl-6-oxo-1,6-dihydropyridazin-4-yl)amino)phenethyl)amino)-4-methoxycyclobut-3-ene-1,2-dione (compound 12): Procedure adapted from Taylor et al.[11] To a solution of 3,4-Dimethoxy-3-cyclobutene-1,2-dione (48.4 mg, 0.33 mmol, 1.1 equiv) in MeOH (5 mL), **BZ1** (99.3 mg, 1.00 mmol, 1 equiv) was added and the reaction was stirred at 27°C for 19 h. After completion of the reaction, white precipitate was formed. However, upon washing with cold MeOH for filtration, the precipitate dissolved. The liquid filtrate was then put on ice, leading to the formation of precipitate. MeOH was removed in vacuo (bath at 25 °C) and the crude product was then dissolved in ethyl acetate and purified using flash column chromatography (Combiflash Rf system, hexanes/ethyl acetate 0-100% ethyl acetate) to obtain **12** as a white solid (63.2 mg, 0.16 mmol, 54%). ^{1}H NMR (400 MHz, $CDCl_3$) δ 7.56 (s, 1H), 7.15 (d, J = 6.7 Hz, 2H), 7.06 (d, J = 8.4 Hz, 2H), 4.24 (d, J = 11.5 Hz, 3H), 3.64 (s, 3H), 3.58 – 3.52 (m, 2H), 2.83 (t, J = 7.2 Hz, 2H). ^{13}C NMR (101 MHz, $CDCl_3$) δ 172.53, 158.46, 142.71, 136.25, 136.02, 135.74, 130.31, 127.36, 124.43, 60.53, 60.35, 45.77, 45.40, 40.27, 36.85, 36.29. HRMS (ESI) calculated for $C_{18}H_{17}ClN_4O_4$ [M+H]$^+$: 389.10110, found 389.0985.

WILEY-VCH

General Procedure B for synthesis of compounds 2-5:

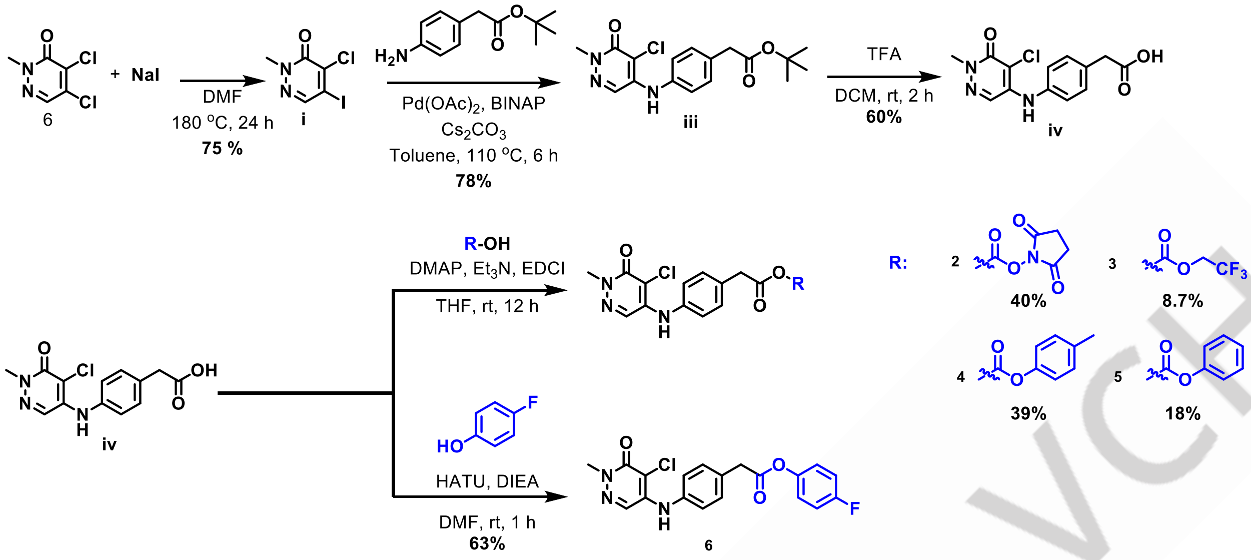

Scheme S2. Chemical synthesis of compounds **2 – 6**

Procedure adapted from Steglich et al.[12] The carboxylic acid (1.0 equiv) was stirred in THF (0.12 M), then to the solution was added DMAP (0.1 equiv), EDCI (2.0 equiv), and triethylamine (2.0 equiv). The mixture was stirred at room temperature for 5 minutes, after which the alcohol (2.5 equiv) was added. The mixture was stirred at room temperature for 20 hours. After completion of the reaction, THF was then removed in vacuo. The resulting crude mixture was then dissolved in ethyl acetate, washed with brine (x5), and purified by flash column chromatography (CombiFlash Rf system, DCM/MeOH, 0-20% methanol) to obtain the final product.

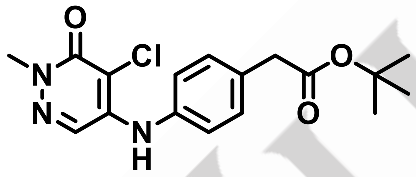

Synthesis of tert-butyl 2-(4-((5-chloro-1-methyl-6-oxo-1,6-dihydropyridazin-4-yl)amino)phenyl)acetate (compound iii): Following General procedure A, tert-butyl 2-(4-aminophenyl)acetate (1.21 g, 5.84 mmol, 1.5 equiv), compound **i** (1g, 3.68 mmol, 1.0 equiv), Pd(OAc)$_2$ (50 mg, 0,22 mmol, 0.06 equiv), BINAP (208 mg, 0.34 mmol, 0.09 equiv), Cesium carbonate (2.9 g, 8.8 mmol, 2.4 equiv) were reacted. Compound **iii** was obtained as a white solid (999 mg, 2.86 mmol, 78%). ^{1}H NMR (400 MHz, $CDCl_3$) δ 7.66 (s, 1H), 7.31 (d, J = 8.4 Hz, 2H), 7.14 (d, J = 8.4 Hz, 2H), 6.45 (s, 1H), 3.75 (s, 3H), 3.54 (s, 2H), 1.44 (s, 9H). ^{13}C NMR (101 MHz, $CDCl_3$) δ 170.65, 157.92, 142.15, 136.27, 133.01, 130.88, 126.79, 124.10, 110.01, 81.30, 42.02, 40.36, 28.14. HRMS (ESI) calculated for $C_{17}H_{20}ClN_3O_3$ $[M+H]^+$: 350.1266, found 350.1268.

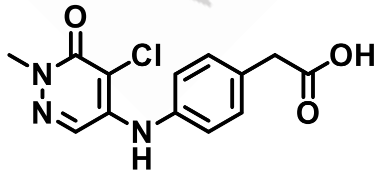

Synthesis of 2-(4-((5-chloro-1-methyl-6-oxo-1,6-dihydropyridazin-4-yl)amino)phenyl)acetic acid (compound iv): Compound **iii** was dissolved in DCM (1-3 mL) at room temperature. To the mixture was added trifluoroacetic acid (5.0 equiv), and the resulting mixture was stirred at room temperature overnight. To the resulting mixture was then added 5 mL 6 M NaOH and 5 mL DCM in a separating funnel. The DCM layer was drained and discarded. Afterwards, 5 mL of 6 M HCl was added and the product was extracted from the aqueous phase using ethyl acetate (x3), dried with anhydrous magnesium sulfate, filtered, and concentrated in vacuo. The resulting crude compound **iv** was used without further purification.

WILEY-VCH

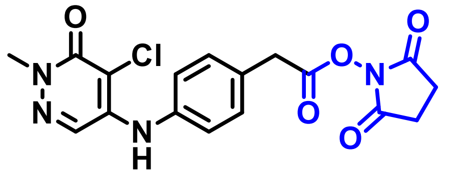

Synthesis of 2,5-dioxopyrrolidin-1-yl 2-(4-((5-chloro-1-methyl-6-oxo-1,6-dihydropyridazin-4-yl)amino)phenyl)acetate (compound 2):): Following general procedure B, 2-(4-((5-chloro-1-methyl-6-oxo-1,6-dihydropyridazin-4-yl)amino)phenyl)acetic acid (106 mg, 0.33 mmol, 1.0 equiv), 1-hydroxypyrrolidine-2,5-dione (95 mg, 0.83 mmol, 2.5 equiv), EDCI (127 mg, 0.66 mmol, 2.0 equiv), DMAP (4 mg, 0.03 mmol, 0.1 equiv), and triethylamine (0.088 mL, 0.66 mmol, 2 equiv) were reacted. Compound **2** was obtained as a yellow solid (51 mg, 0.13 mmol, 40 %). ^{1}H NMR (400 MHz, $CDCl_3$) δ 7.69 (s, 1H), 7.40 (d, *J* = 8.4 Hz, 2H), 7.19 (d, *J* = 8.4 Hz, 2H), 6.41 (s, 1H), 3.95 (s, 2H), 3.77 (s, 3H), 2.85 (s, 4H). ^{13}C NMR (101 MHz, $CDCl_3$) δ 172.16, 169.18, 166.64, 158.20, 142.13, 137.15, 131.04, 129.58, 127.03, 124.22, 110.42, 40.59, 37.15, 25.71, 25.51. HRMS (ESI) calculated for $C_{17}H_{15}ClN_4O_5$ $[M+H]^+$: 391.0804, found 391.0773.

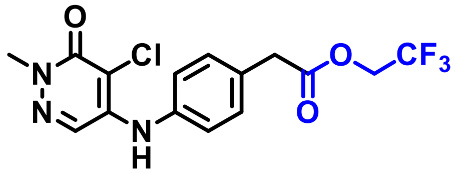

Synthesis of 2,2,2-trifluoroethyl 2-(4-((5-chloro-1-methyl-6-oxo-1,6-dihydropyridazin-4-yl)amino)phenyl)acetate (compound 3): Following general procedure B, 2-(4-((5-chloro-1-methyl-6-oxo-1,6-dihydropyridazin-4-yl)amino)phenyl)acetic acid (800 mg, 2.5 mmol, 1.0 equiv), 2,2,2-trifluoroethanol (625 mg, 6.25 mmol, 2.5 equiv), EDCI (959 mg, 5.0 mmol, 2.0 equiv), DMAP (31 mg, 0.25 mmol, 0.1 equiv), and triethylamine (0.67 mL, 5 mmol, 2 equiv) were reacted. Compound **3** was obtained as a yellow solid (82 mg, 0.22 mmol, 8.7 %). ^{1}H NMR (400 MHz, $CDCl_3$) δ 7.67 (s, 1H), 7.33 (d, *J* = 8.4 Hz, 2H), 7.17 (d, *J* = 8.4 Hz, 2H), 6.46 (s, 1H), 4.50 (q, *J* = 8.4 Hz, 2H), 3.76 (s, 3H), 3.75 (s, 2H). ^{13}C NMR (101 MHz, $CDCl_3$) δ 169.77, 157.92, 141.98, 136.97, 130.97, 130.92, 126.74, 124.15, 113.63, 110.42, 60.82 (q, *J* = 36.7 Hz), 40.42, 40.00. ^{19}F NMR (376 MHz, $CDCl_3$) δ -73.75. HRMS (ESI) calculated for $C_{15}H_{13}ClF_3N_3O_3$ $[M+H]^+$: 376.0670, found 376.0657.

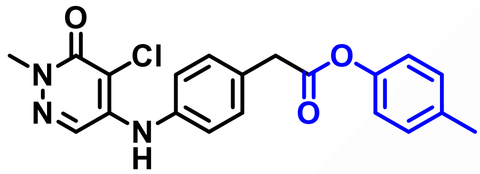

Synthesis of p-tolyl 2-(4-((5-chloro-1-methyl-6-oxo-1,6-dihydropyridazin-4-yl)amino)phenyl)acetate (compound 4): Following general procedure B, with 2-(4-((5-chloro-1-methyl-6-oxo-1,6-dihydropyridazin-4-yl)amino)phenyl)acetic acid (63 mg, 0.2 mmol, 1.0 equiv), *p*-cresol (54 mg, 0.5 mmol, 2.5 equiv), EDCI (77 mg, 0.4 mmol, 2.0 equiv), DMAP (2.4 mg, 0.02 mmol, 0.1 equiv), and triethylamine (0.05 mL, 0.4 mmol, 2 equiv) were reacted. Compound **4** was obtained as a yellow solid (30 mg, 0.07 mmol, 39%). ^{1}H NMR (400 MHz, $CDCl_3$) δ 7.69 (s, 1H), 7.42 (d, *J* = 8.6 Hz, 2H), 7.18 (d, *J* = 8.3 Hz, 2H), 7.15 (d, *J* = 8.7 Hz, 2H), 6.94 (d, *J* = 8.6 Hz, 2H), 6.48 (s, 1H), 3.86 (s, 2H), 3.76 (s, 3H), 2.33 (s, 3H). ^{13}C NMR (101 MHz, $CDCl_3$) δ 169.97, 157.91, 148.48, 142.02, 136.76, 135.79, 131.70, 131.00, 130.05, 126.76, 124.15, 121.12, 110.28, 40.80, 40.40, 20.96. HRMS (ESI) calculated for $C_{20}H_{18}ClN_3O_3$ $[M+H]^+$: 384.1109, found 384.1080.

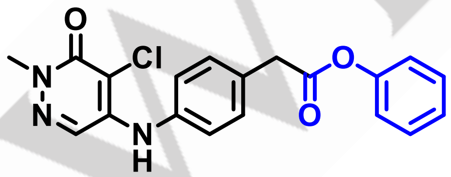

Synthesis of phenyl 2-(4-((5-chloro-1-methyl-6-oxo-1,6-dihydropyridazin-4-yl)amino)phenyl)acetate (compound 5): Following general procedure B, 2-(4-((5-chloro-1-methyl-6-oxo-1,6-dihydropyridazin-4-yl)amino)phenyl)acetic acid (264 mg, 0.81 mmol, 1.0 equiv), phenol (191 mg, 2.03 mmol, 2.5 equiv), EDCI (310 mg, 1.62 mmol, 2.0 equiv), DMAP (10 mg, 0.08 mmol, 0.1 equiv), and triethylamine (0.22 mL, 1.62 mmol, 2.0 equiv) were reacted. Compound **5** was obtained as a brown solid (53 mg, 0.14 mmol, 18%). ^{1}H NMR (400 MHz, $CDCl_3$) δ 7.69 (s, 1H), 7.43 (d, *J* = 8.4 Hz, 2H), 7.37 (t, *J* = 7.7 Hz, 2H), 7.24 (d, *J* = 7.5 Hz, 1H), 7.19 (d, *J* = 8.3 Hz, 2H), 7.07 (d, *J* = 7.5 Hz, 2H), 6.48 (s, 1H), 3.88 (s, 2H), 3.76 (s, 3H). ^{13}C NMR (101 MHz, $CDCl_3$) δ 169.71, 157.86, 150.63, 141.95, 136.70, 131.54, 130.94, 129.48, 126.70, 126.05, 124.09, 121.38, 110.22, 40.73, 40.35. HRMS (ESI) calculated for $C_{19}H_{16}ClN_3O_3$ $[M+H]^+$: 370.0953, found 370.0931.

WILEY-VCH

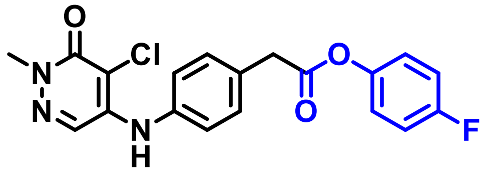

Synthesis of 4-fluorophenyl 2-(4-((5-chloro-1-methyl-6-oxo-1,6-dihydropyridazin-4-yl)amino)phenyl)acetate (compound 6): Procedure adapted from Dalton et al.[13] To a vial containing dimethyl formamide (1 mL) was added 2-(4-((5-chloro-1-methyl-6-oxo-1,6-dihydropyridazin-4-yl)amino)phenyl)acetic acid (100 mg, 0.34 mmol, 1 equiv), HATU (155 mg, 0.41 mmol, 1.2 equiv), DIEA (0.12 mL, 0.68 mmol, 2 equiv). The mixture was stirred for 5 minutes and then 4-fluorophenol (57 mg, 0.51 mmol, 1.5 equiv) was added. Stirring was continued for 3 hours. Upon completion of the reaction, mixture was transferred to a separating funnel using ethyl acetate and washed with 8 M lithium chloride (x3). The organic layer was then dried using anhydrous magnesium sulfate, filtered, and purified by flash column chromatography (CombiFlash Rf system, hexanes/ethyl acetate, 0-100% ethyl acetate) to obtain compound **6** as a white solid (84 mg, 0.22 mmol, 63%). ^{1}H NMR (400 MHz, $CDCl_3$) δ 7.70 (s, 1H), 7.42 (d, *J* = 8.6 Hz, 2H), 7.20 (d, *J* = 8.4 Hz, 2H), 7.05 (d, *J* = 0.9 Hz, 2H), 7.03 (s, 2H), 6.51 (s, 1H), 3.87 (s, 2H), 3.77 (s, 3H). ^{13}C NMR (101 MHz, $CDCl_3$) δ 169.84, 161.62, 159.19, 157.93, 146.51, 142.00, 136.89, 131.41, 131.02, 126.76, 124.18, 122.90 (d, *J* = 8.8 Hz), 116.23 (d, *J* = 23.5 Hz), 110.39, 40.70, 40.44. ^{19}F NMR (376 MHz, $CDCl_3$) δ -116.58. HRMS (ESI) calculated for $C_{19}H_{15}ClFN_3O_3$ $[M+H]^+$: 388.0859, found 388.0823.

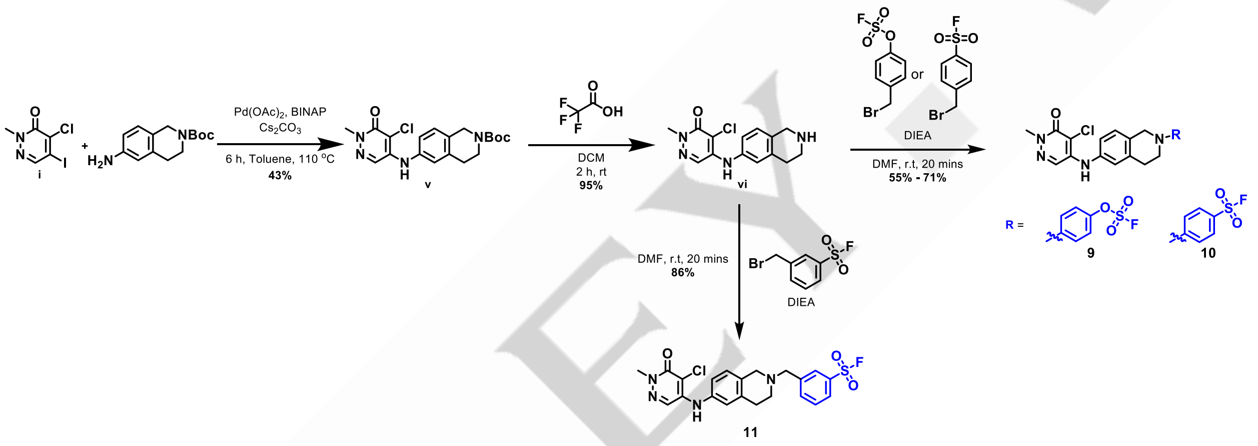

Scheme S3. Chemical synthesis of compounds **9 – 11**

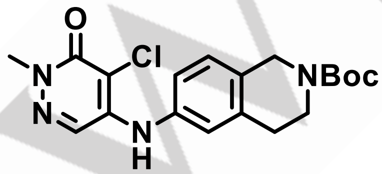

Synthesis of tert-butyl 6-((5-chloro-1-methyl-6-oxo-1,6-dihydropyridazin-4-yl)amino)-3,4-dihydroisoquinoline-2(1H)-carboxylate (compound v): Following General procedure A, tert-butyl 6-amino-3,4-dihydroisoquinoline-2(1H)-carboxylate (1.4 g, 5.6 mmol, 1.5 equiv), compound **i** (1g, 3.70 mmol, 1.0 equiv), $Pd(OAc)_2$ (45 mg, 0.2 mmol, 0.06 equiv), BINAP (187 mg, 0.3 mmol, 0.09 equiv), Cesium carbonate (2.9 g, 8.9 mmol, 2.4 equiv) were reacted. Compound **v** was obtained as a brown solid (620 mg, 1.6 mmol, 43%). ^{1}H NMR (400 MHz, $CDCl_3$) δ 7.64 (s, 1H), 7.15 (d, *J* = 8.2 Hz, 1H), 7.02 (d, *J* = 2.5 Hz, 1H), 6.98 (s, 1H), 6.39 (s, 1H), 4.57 (s, 2H), 3.76 (s, 3H), 3.65 (t, *J* = 5.9 Hz, 2H), 2.83 (t, *J* = 5.8 Hz, 2H), 1.49 (s, 9H). Spectral characterization for this molecule was consistent with previous report.[9]

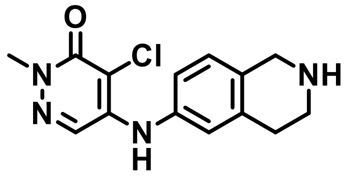

Synthesis of 4-chloro-2-methyl-5-((1,2,3,4-tetrahydroisoquinolin-6-yl)amino)pyridazin-3(2H)-one (compound vi): Step 1: The purified compound **v** (620 mg, 1.6 mmol, 1 equiv) was then dissolved in DCM (1-3 mL) at room temperature. To the mixture was added trifluoroacetic acid (5.0 equiv), and the resulting mixture was stirred at room temperature overnight. Step 2: DCM was then removed under a stream of nitrogen. To obtain compound **vi** as a free base, the mixture was extracted into DCM and then adjusted to a pH > 10 using a solution of 1 M NaOH. The organic layer was then dried using anhydrous magnesium sulfate, filtered, and DCM removed in vacuo to furnish the product. The crude product was used for the next step without further purification.

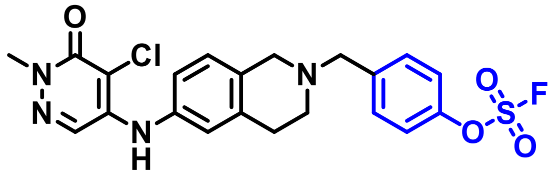

Synthesis of 4-((6-((5-chloro-1-methyl-6-oxo-1,6-dihydropyridazin-4-yl)amino)-3,4-dihydroisoquinolin-2(1H)-yl)methyl)phenyl sulfurofluoridate (compound 9): Procedure adapted from Zhao et al.[14] To a mixture of compound **vi** (15.7 mg, 0.05 mmol, 1.0 equiv) and 4-(bromomethyl)phenyl sulfurofluoridate (15 mg, 0.06 mmol, 1.0 equiv) in DMF (0.15 M) was added DIEA (0.04 mL, 0.22 mmol, 4 equiv). The reaction was stirred at room temperature for 20 minutes. After completion of the reaction, DMF was removed in a separating funnel by washing with 8 M LiCl (x3) and extracting into ethyl acetate (x3). Mixture was washed with sodium bicarobonate, saturated brine, dried over anhydrous magnesium sulfate, and concentrated in vacuo. Purification was done by flash column chromatography (CombiFlash Rf system, hexanes/ethyl acetate, 0-100% ethyl acetate) to obtain compound **9** as a white solid (14 mg, 0.03 mmol, 55%). ^{1}H NMR (400 MHz, $CDCl_3$) δ 7.63 (s, 1H), 7.54 (d, *J* = 7.8 Hz, 2H), 7.32 (d, *J* = 8.7 Hz, 2H), 7.05 (d, *J* = 8.9 Hz, 1H), 6.96 (d, *J* = 7.0 Hz, 2H), 6.37 (s, 1H), 3.76 (s, 5H), 3.67 (s, 2H), 2.94 (s, 2H), 2.80 (s, 2H). ^{13}C NMR (101 MHz, $CDCl_3$) δ 157.92, 149.39, 142.30, 136.10, 135.69, 130.98, 128.22, 126.81, 124.32, 121.95, 121.02, 61.54, 55.49, 53.70, 50.32, 40.37, 29.02, 18.72, 17.52. ^{19}F NMR (376 MHz, $CDCl_3$) δ 37.59. HRMS (ESI) calculated for $C_{21}H_{20}ClFN_4O_4S$ $[M+H]^+$: 479.0951, found 479.0953.

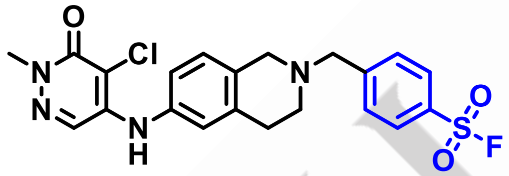

Synthesis of 4-((6-((5-chloro-1-methyl-6-oxo-1,6-dihydropyridazin-4-yl)amino)-3,4-dihydroisoquinolin-2(1H)-yl)methyl)benzenesulfonyl fluoride (compound 10): Procedure adapted from Zhao et al.[14] To a mixture of compound **vi** (50 mg, 0.17 mmol, 1.0 equiv) and 4-(bromomethyl)benzenesulfonyl fluoride (44 mg, 0.18 mmol, 1.0 equiv) in DMF (0.15 M) was added DIEA (0.12 mL, 0.68 mmol, 4 equiv). The reaction was stirred at room temperature for 20 minutes. After completion of the reaction, DMF was removed in a separating funnel by washing with 8 M LiCl (x3) and extracting into ethyl acetate (x3). Mixture was washed with sodium bicarobonate, saturated brine, dried over anhydrous magnesium sulfate, and concentrated in vacuo. Purification was done by flash column chromatography (CombiFlash Rf system, hexanes/ethyl acetate, 0-100% ethyl acetate) to obtain compound **10** as a purple solid (56 mg, 0.12 mmol, 71%). ^{1}H NMR (400 MHz, $CDCl_3$) δ 7.97 (d, *J* = 8.4 Hz, 2H), 7.68 (d, *J* = 8.3 Hz, 2H), 7.63 (s, 1H), 7.03 (d, *J* = 7.9 Hz, 1H), 6.96 (d, *J* = 7.6 Hz, 2H), 6.41 (s, 1H), 3.81 (s, 2H), 3.74 (s, 3H), 3.65 (s, 2H), 2.92 (t, *J* = 5.9 Hz, 2H), 2.77 (t, *J* = 5.9 Hz, 2H). ^{13}C NMR (101 MHz, $CDCl_3$) δ 157.91, 147.57, 142.30, 136.15, 135.65, 131.98, 131.74, 129.91, 128.72, 128.14, 126.80, 124.35, 121.92, 109.76, 61.98, 55.76, 50.58, 40.37, 29.27. ^{19}F NMR (376 MHz, $CDCl_3$) δ 66.12. HRMS (ESI) calculated for $C_{21}H_{20}ClFN_4O_3S$ $[M+H]^+$: 463.1001, found 463.1011.

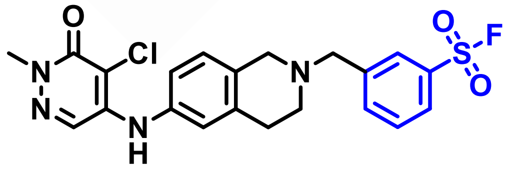

Synthesis of 3-((6-((5-chloro-1-methyl-6-oxo-1,6-dihydropyridazin-4-yl)amino)-3,4-dihydroisoquinolin-2(1H)-yl)methyl)benzenesulfonyl fluoride (compound 11): Procedure adapted from Zhao et al.[14] To a mixture of compound **vi** (25 mg, 0.09 mmol, 1.0 equiv) and 3-(bromomethyl)benzenesulfonyl fluoride (22 mg, 0.09 mmol, 1.0 equiv) in DMF (0.15 M) was added DIEA (0.06 mL, 0.34 mmol, 4 equiv). The reaction was stirred at room temperature for 20 minutes. After completion of the reaction, DMF was removed in a separating funnel by washing with 8 M LiCl (x3) and extracting into ethyl acetate (x3).

Mixture was washed with sodium bicarobonate, saturated brine, dried over anhydrous magnesium sulfate, and concentrated in vacuo. Purification was done by flash column chromatography (CombiFlash Rf system, hexanes/ethyl acetate, 0-100% ethyl acetate) to obtain compound **11** as a purple solid (34 mg, 0.07 mmol, 86%). ^{1}H NMR (400 MHz, $CDCl_3$) δ 8.07 (s, 1H), 7.94 (d, *J* = 9.4 Hz, 1H), 7.88 (s, 1H), 7.66 – 7.58 (m, 2H), 7.04 (d, *J* = 9.0 Hz, 1H), 7.00 – 6.95 (m, 2H), 6.40 (s, 1H), 3.84 (s, 2H), 3.75 (s, 3H), 3.69 (s, 2H), 2.94 (s, 2H), 2.82 (s, 2H). ^{13}C NMR (101 MHz, $CDCl_3$) δ 157.95, 142.26, 136.36, 135.92, 135.77, 133.60, 133.36, 130.09, 128.81, 128.22, 127.77, 126.84, 124.27, 122.03, 109.89, 61.22, 55.22, 50.25, 40.40, 28.72, 28.52. ^{19}F NMR (376 MHz, $CDCl_3$) δ 65.88. HRMS (ESI) calculated for $C_{21}H_{20}ClFN_4O_3S$ $[M+H]^+$: 463.1001, found 463.0953.

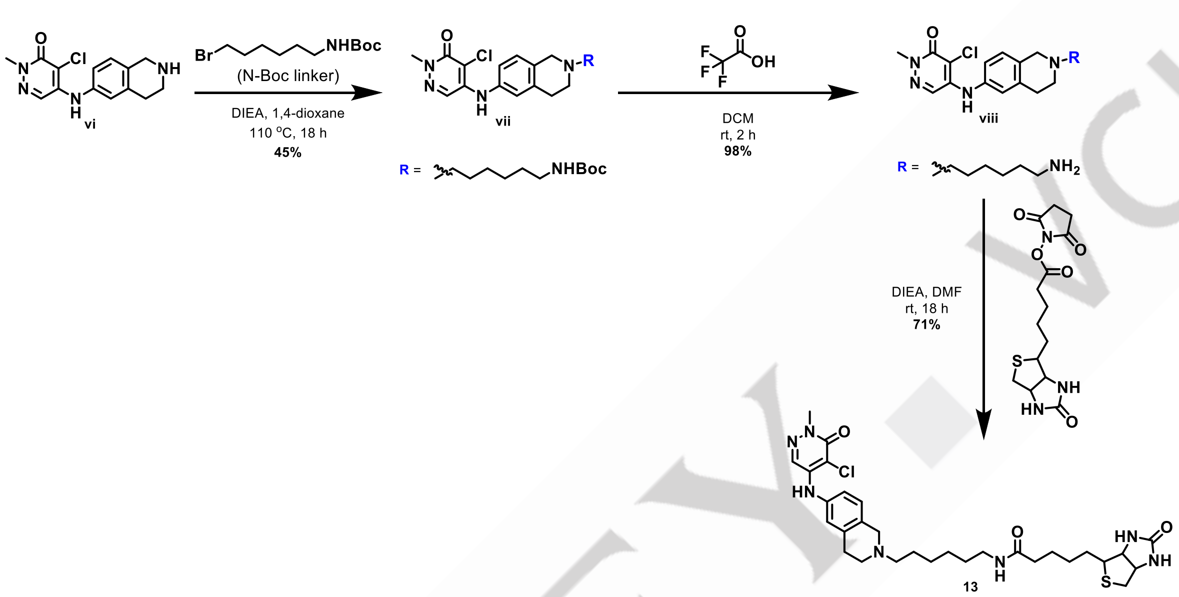

Scheme S4. Chemical synthesis of compound **13**

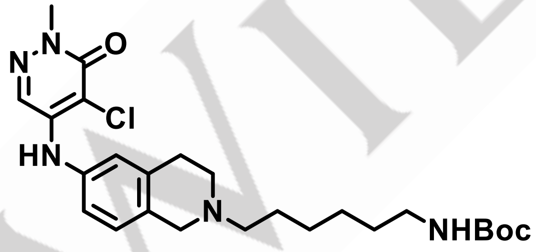

Synthesis of tert-butyl (6-(6-((5-chloro-1-methyl-6-oxo-1,6-dihydropyridazin-4-yl)amino)-3,4-dihydroisoquinolin-2(1H)-yl)hexyl)carbamate (compound vii): To solution of compound **vi** (155 mg, 0.53 mmol, 1.0 equiv) stirring in 1,4-dioxane (0.21 M) at room temperature was added *N*-Boc linker (0.163 mg, 0.58 mmol, 1.1 equiv) and *N*,*N*-diisopropylethylamine (0.139 mL, 0.8 mmol, 1.5 eq). The reaction mixture was then heated to 110 °C in a sealed tube for 18 h. Upon completion of the reaction confirmed by TLC, the 1,4-dioxane was removed using rotary evaporation. The crude mixture was then extracted into ethyl acetate and washed with a saturated solution of brine (10 mL) and sodium bicarbonate (10 mL). Afterwards, the organic layer was dried over magnesium sulfate, filtered, concentrated using rotary evaporation, and purified by flash column chromatography (CombiFlash Rf system, hexanes/ethyl acetate, 0-100% ethyl acetate) to furnish compound **vii** as a white solid (117 mg, 0.24 mmol, 45%). Spectra data was consistent with previous report.[15] ^{1}H NMR (400 MHz, $CDCl_3$) δ 7.60 (s, 1H), 7.05 (d, *J* = 8.8 Hz, 1H), 6.97 – 6.90 (m, 2H), 6.40 (s, 1H), 4.55 (s, 1H), 3.74 (s, 3H), 3.61 (s, 2H), 3.10 (q, *J* = 6.7 Hz, 2H), 2.89 (t, *J* = 5.9 Hz, 2H), 2.72 (t, *J* = 6.0 Hz, 2H), 2.50 (dd, *J* = 8.6, 6.7 Hz, 2H), 1.64 – 1.54 (m, 2H), 1.51 – 1.40 (m, 11H), 1.39 – 1.31 (m, 4H).

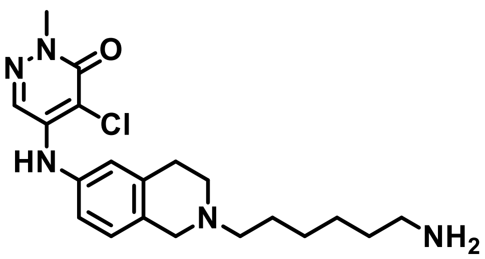

Synthesis of N-(6-(6-((5-chloro-1-methyl-6-oxo-1,6-dihydropyridazin-4-yl)amino)-3,4-dihydroisoquinolin-2(1H)-yl)hexyl)-5-(2-oxohexahydro-1H-thieno[3,4-d]imidazol-4-yl)pentanamide (compound viii): Step 1: The purified compound **vii** (117 mg, 0.24 mmol, 1 equiv) was then dissolved in DCM (1-3 mL) at room temperature. To the mixture was added trifluoroacetic acid (5.0 equiv), and the resulting mixture was stirred at room temperature overnight. Step 2: DCM was then removed under a stream of nitrogen. To obtain compound **vi** as a free base, the mixture was extracted into DCM and then adjusted to a pH > 10 using a solution of 1 M NaOH. The organic layer was then dried using anhydrous magnesium sulfate, filtered, and DCM removed in vacuo to furnish the product. The crude product was used for the next step without further purification.

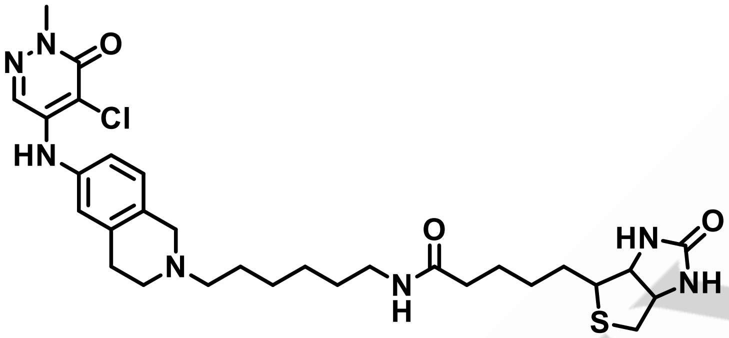

Synthesis of N-(6-(6-((5-chloro-1-methyl-6-oxo-1,6-dihydropyridazin-4-yl)amino)-3,4-dihydroisoquinolin-2(1H)-yl)hexyl)-5-(2-oxohexahydro-1H-thieno[3,4-d]imidazol-4-yl)pentanamide (compound 13): To a round bottom flask containing compound **vii** (73 mg, 0.19 mmol, 1 equiv) in DCM (0.15 M) was added 2,5-dioxopyrrolidin-1-yl 5-(2-oxohexahydro-1H-thieno[3,4-d]imidazol-4-yl)pentanoate (72 mg, 0.21 mmol, 1.1 equiv), and triethyl amine (0.05 mL, 0.38 mmol, 2 equiv). The mixture was stirred at room temperature for 16 hours. Following completion of the reaction, purification was done by flash column chromatography (CombiFlash Rf system, DCM/methanol, 0-40% methanol) to obtain compound **13** as a yellow solid (84 mg, 0.14 mmol, 71%). ^{1}H NMR (400 MHz, $CDCl_3$) δ 7.60 (s, 1H), 7.06 (d, *J* = 8.8 Hz, 1H), 6.98 – 6.91 (m, 2H), 6.53 (s, 1H), 6.37 (s, 1H), 6.16 (t, *J* = 5.7 Hz, 1H), 5.63 (s, 1H), 4.47 (dd, *J* = 7.8, 4.9 Hz, 1H), 4.32 – 4.24 (m, 1H), 3.74 (s, 3H), 3.62 (s, 2H), 3.21 (q, *J* = 6.5 Hz, 2H), 3.15 – 3.08 (m, 1H), 2.93 – 2.83 (m, 3H), 2.80 – 2.66 (m, 3H), 2.51 (t, *J* = 7.6 Hz, 3H), 2.18 (t, *J* = 7.5 Hz, 2H), 1.79 – 1.53 (m, 6H), 1.50 (t, *J* = 7.2 Hz, 2H), 1.42 (q, *J* = 7.5 Hz, 2H), 1.35 (t, *J* = 3.7 Hz, 3H). HRMS (ESI) calculated for $C_{30}H_{42}ClN_7O_3S$ $[M+H]^+$: 616.2831, found 616.2804. HPLC-QC; 98.8% (220 nm), 99.2% (272 nm).

References

[1] K. E. Peterson, N. M. Olson, D. J. Dahlseid, J. M. Artymiuk, L. Erber, F. N. L. Vitorino, R. Dean, J. W. Landry, N. Y. Tretyakova, B. A. Garcia, W. C. K. Pomerantz, "BPTF Target Engagement by Acetylated H2A.Z Photoaffinity Probes" *Biochemistry* **2025**, *64*, 3872–3885.
[2] L. Huang, H. Li, L. Li, L. Niu, R. Seupel, C. Wu, W. Cheng, C. Chen, B. Ding, P. E. Brennan, S. Yang, "Discovery of Pyrrolo[3,2- *d*]pyrimidin-4-one Derivatives as a New Class of Potent and Cell-Active Inhibitors of P300/CBP-Associated Factor Bromodomain" *J. Med. Chem.* **2019**, *62*, 4526–4542.
[3] X. Wan, T. Yang, A. Cuesta, X. Pang, T. E. Balius, J. J. Irwin, B. K. Shoichet, J. Taunton, "Discovery of Lysine-Targeted eIF4E Inhibitors through Covalent Docking" *J. Am. Chem. Soc.* **2020**, *142*, 4960–4964.
[4] P. G. Humphreys, P. Bamborough, C. Chung, P. D. Craggs, L. Gordon, P. Grandi, T. G. Hayhow, J. Hussain, K. L. Jones, M. Lindon, A.-M. Michon, J. F. Renaux, C. J. Suckling, D. F. Tough, R. K. Prinjha, "Discovery of a Potent, Cell Penetrant, and Selective p300/CBP-Associated Factor (PCAF)/General Control Nonderepressible 5 (GCN5) Bromodomain Chemical Probe" *J. Med. Chem.* **2017**, *60*, 695–709.
[5] T. A. Halgren, R. B. Murphy, R. A. Friesner, H. S. Beard, L. L. Frye, W. T. Pollard, J. L. Banks, "Glide: A New Approach for Rapid, Accurate Docking and Scoring. 2. Enrichment Factors in Database Screening" *J. Med. Chem.* **2004**, *47*, 1750–1759.
[6] K. Zhu, K. W. Borrelli, J. R. Greenwood, T. Day, R. Abel, R. S. Farid, E. Harder, "Docking covalent inhibitors: a parameter free approach to pose prediction and scoring" *J Chem Inf Model* **2014**, *54*, 1932–1940.
[7] J. P. Wolfe, S. Wagaw, S. L. Buchwald, "An Improved Catalyst System for Aromatic Carbon−Nitrogen Bond Formation: The Possible Involvement of Bis(Phosphine) Palladium Complexes as Key Intermediates" *J. Am. Chem. Soc.* **1996**, *118*, 7215–7216.
[8] J. P. Wolfe, S. L. Buchwald, "Scope and Limitations of the Pd/BINAP-Catalyzed Amination of Aryl Bromides" *J. Org. Chem.* **2000**, *65*, 1144–1157.
[9] P. Dunkel, G. Túrós, A. Bényei, K. Ludányi, P. Mátyus, "Synthesis of novel fused azecine ring systems through application of the tert-amino effect" *Tetrahedron* **2010**, *66*, 2331–2339.
[10] H. Zahid, C. R. Buchholz, M. Singh, M. F. Ciccone, A. Chan, S. Nithianantham, K. Shi, H. Aihara, M. Fischer, E. Schönbrunn, C. O. dos Santos, J. W. Landry, W. C. K. Pomerantz, "New Design Rules for Developing Potent Cell-Active Inhibitors of the Nucleosome Remodeling Factor (NURF) via BPTF Bromodomain Inhibition" *J. Med. Chem.* **2021**, *64*, 13902–13917.
[11] D. A. Shannon, R. Banerjee, E. R. Webster, D. W. Bak, C. Wang, E. Weerapana, "Investigating the Proteome Reactivity and Selectivity of Aryl Halides" *J. Am. Chem. Soc.* **2014**, *136*, 3330–3333.
[12] K. I. Taylor, J. S. Ho, H. O. Trial, A. W. Carter, L. L. Kiessling, "Assessing Squarates as Amine-Reactive Probes" *J. Am. Chem. Soc.* **2023**, DOI 10.1021/jacs.2c05691.
[13] B. Neises, W. Steglich, "Simple Method for the Esterification of Carboxylic Acids" *Angewandte Chemie International Edition in English* **1978**, *17*, 522–524.
[14] S. E. Dalton, L. Dittus, D. A. Thomas, M. A. Convery, J. Nunes, J. T. Bush, J. P. Evans, T. Werner, M. Bantscheff, J. A. Murphy, S. Campos, "Selectively Targeting the Kinome-Conserved Lysine of PI3Kδ as a General Approach to Covalent Kinase Inhibition" *J. Am. Chem. Soc.* **2018**, *140*, 932–939.
[15] Q. Zhao, X. Ouyang, X. Wan, K. S. Gajiwala, J. C. Kath, L. H. Jones, A. L. Burlingame, J. Taunton, "Broad-Spectrum Kinase Profiling in Live Cells with Lysine-Targeted Sulfonyl Fluoride Probes" *J. Am. Chem. Soc.* **2017**, *139*, 680–685.
[16] H. Zahid, J. Costello, J. Kimbrough, M. Actis, Z. Rankovic, W. Pomerantz, *Design of Class I/IV Bromodomain-Targeting Degraders for Chromatin Remodeling Complexes*, Chemistry, **2022**.

NMR Spectra

Cpd ii, ^{1}H-NMR, 400 MHz, $CDCl_{3s}$

wporre-240329-7.10.fid — RRE-3-44 BB S1 — PROTON CDCl3 /opt/data wporre 7

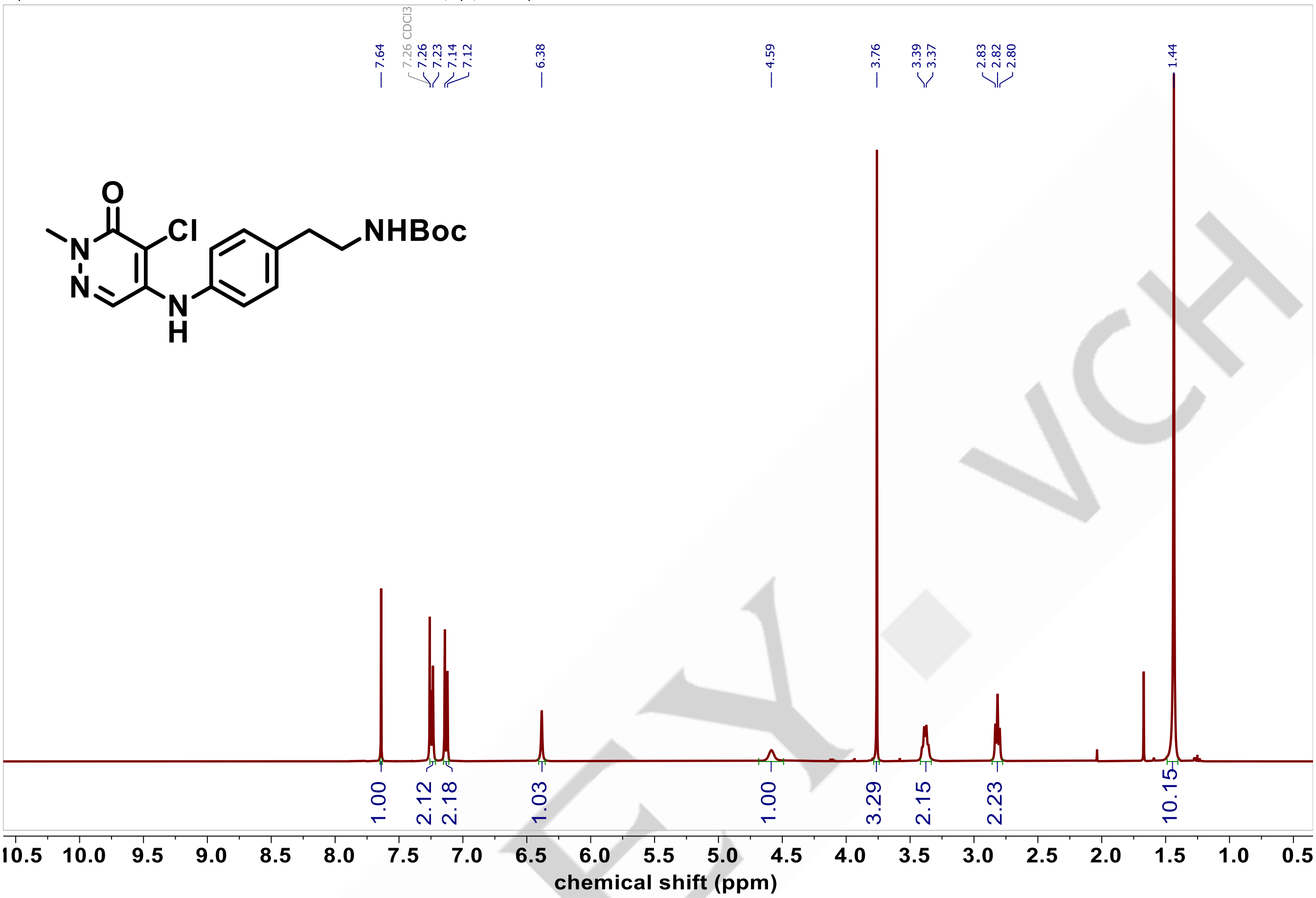

WILEY-VCH

1, ^{1}H-NMR, 400 MHz, $CDCl_3$

wporre-250723-10.10.fid — RRE-5-2 Post dessicator — PROTON DMSO /opt/data wporre 10

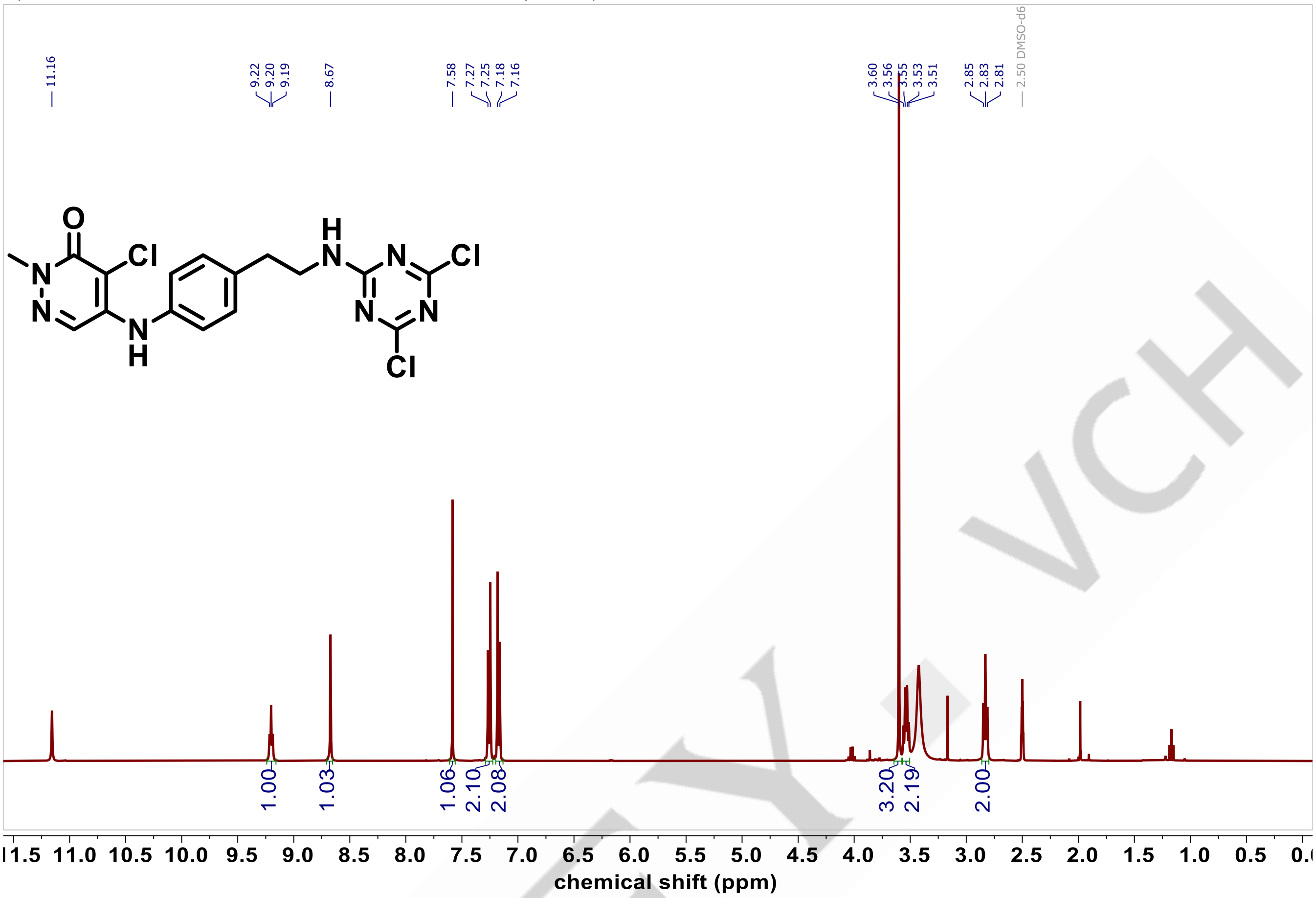

WILEY-VCH

1, ^{13}C-NMR, 101 MHz, $CDCl_3$

wporre-250326-4.11.fid — RRE-ZV140 (triazine) carbon — C13udeft DMSO /opt/data wporre 4

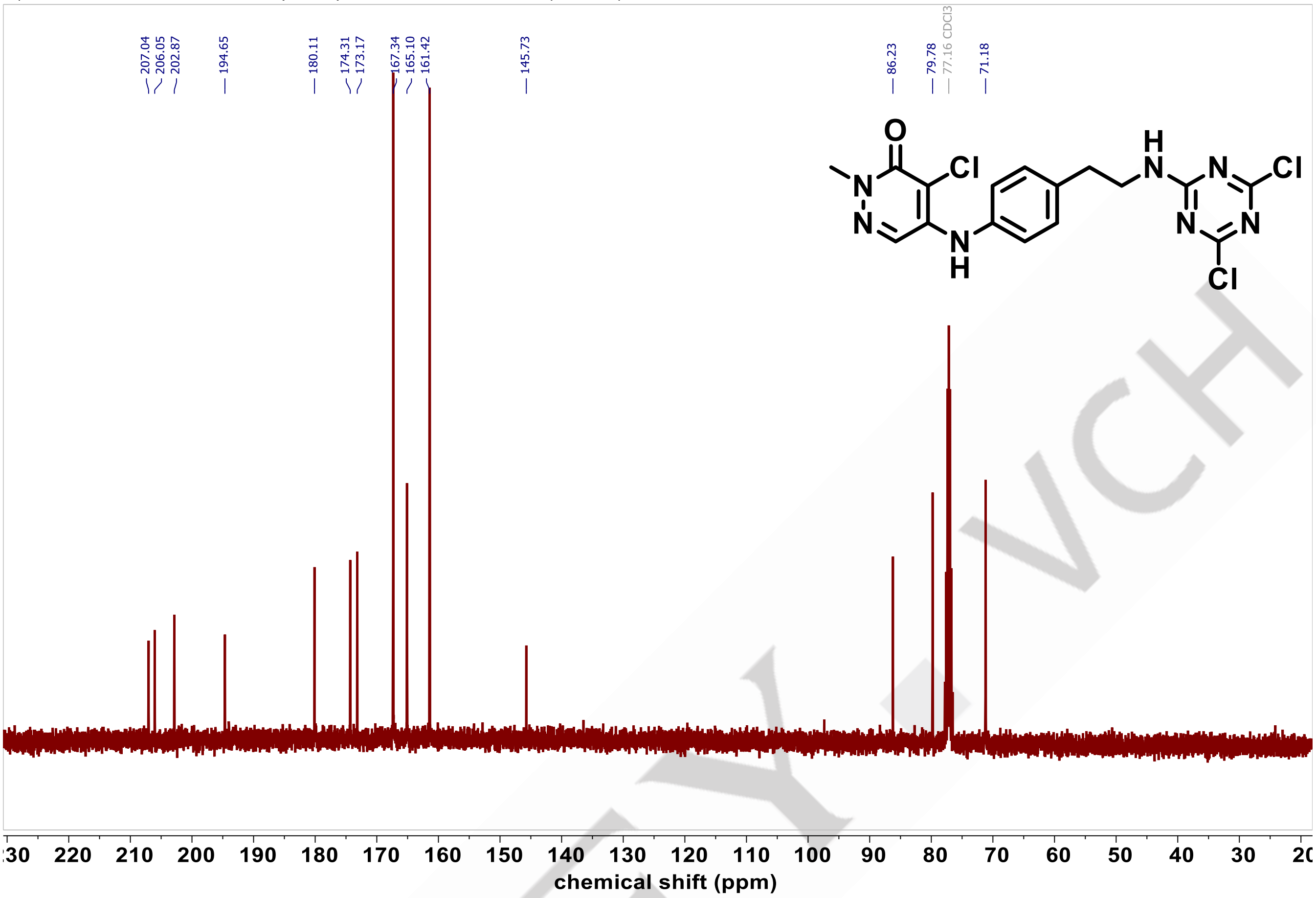

WILEY-VCH

Cpd iii, ^{1}H-NMR, 400 MHz, $CDCl_3$

wporre-230228-60.10.fid — RRE-1-104 PRO — PROTON CDCl3 /opt/data wporre 60

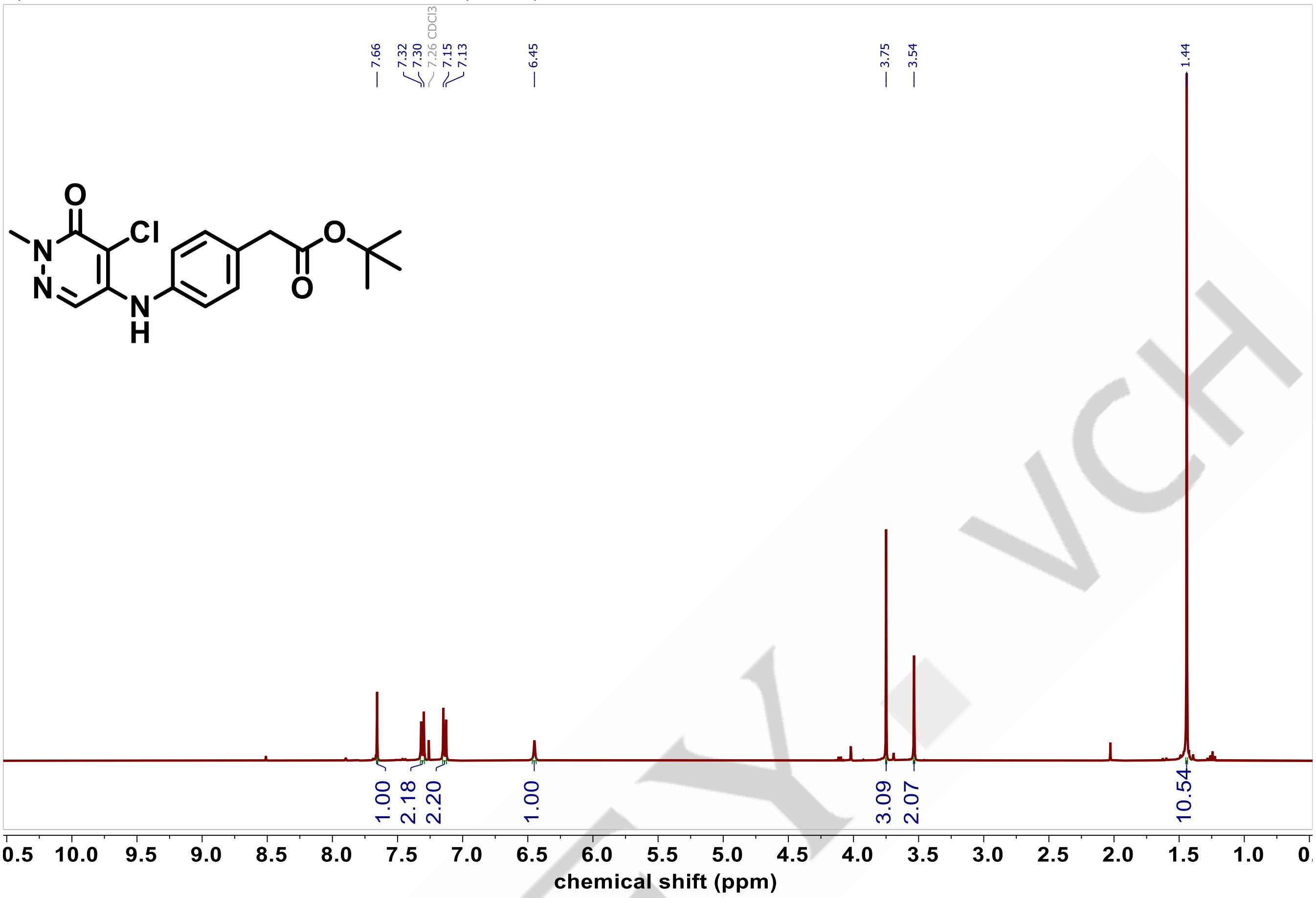

Cpd iii, ^{13}C-NMR, 101 MHz, $CDCl_3$

wporre-251217-5.11.fid — RRE-5-70 13C regular — C13udeft CDCl3 /opt/data wporre 5

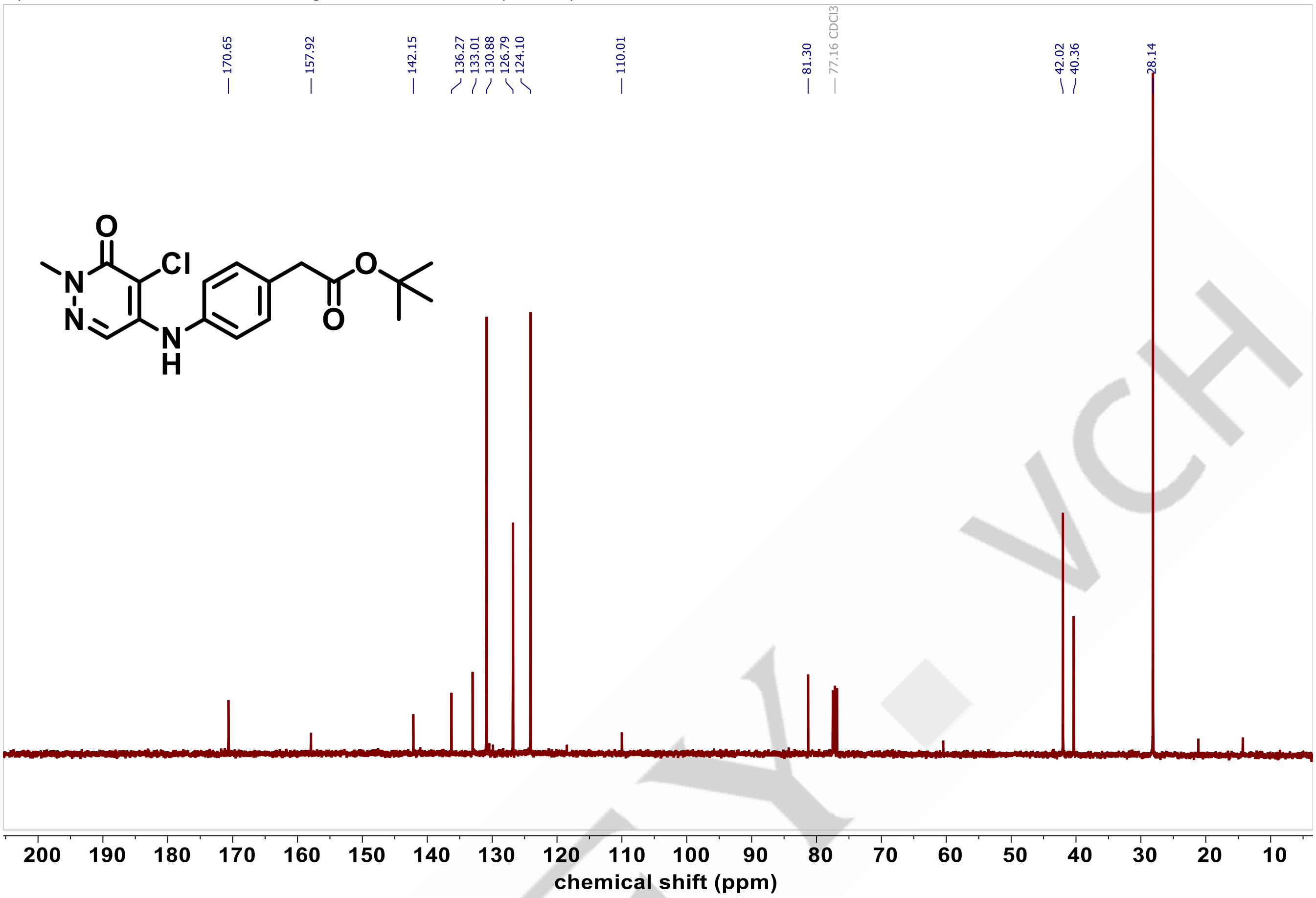

Cpd v, ^{1}H-NMR, 400 MHz, $CDCl_3$

wporre-240313-32.10.fid — RRE-3-32 PRO — PROTON CDCl3 /opt/data wporre 32

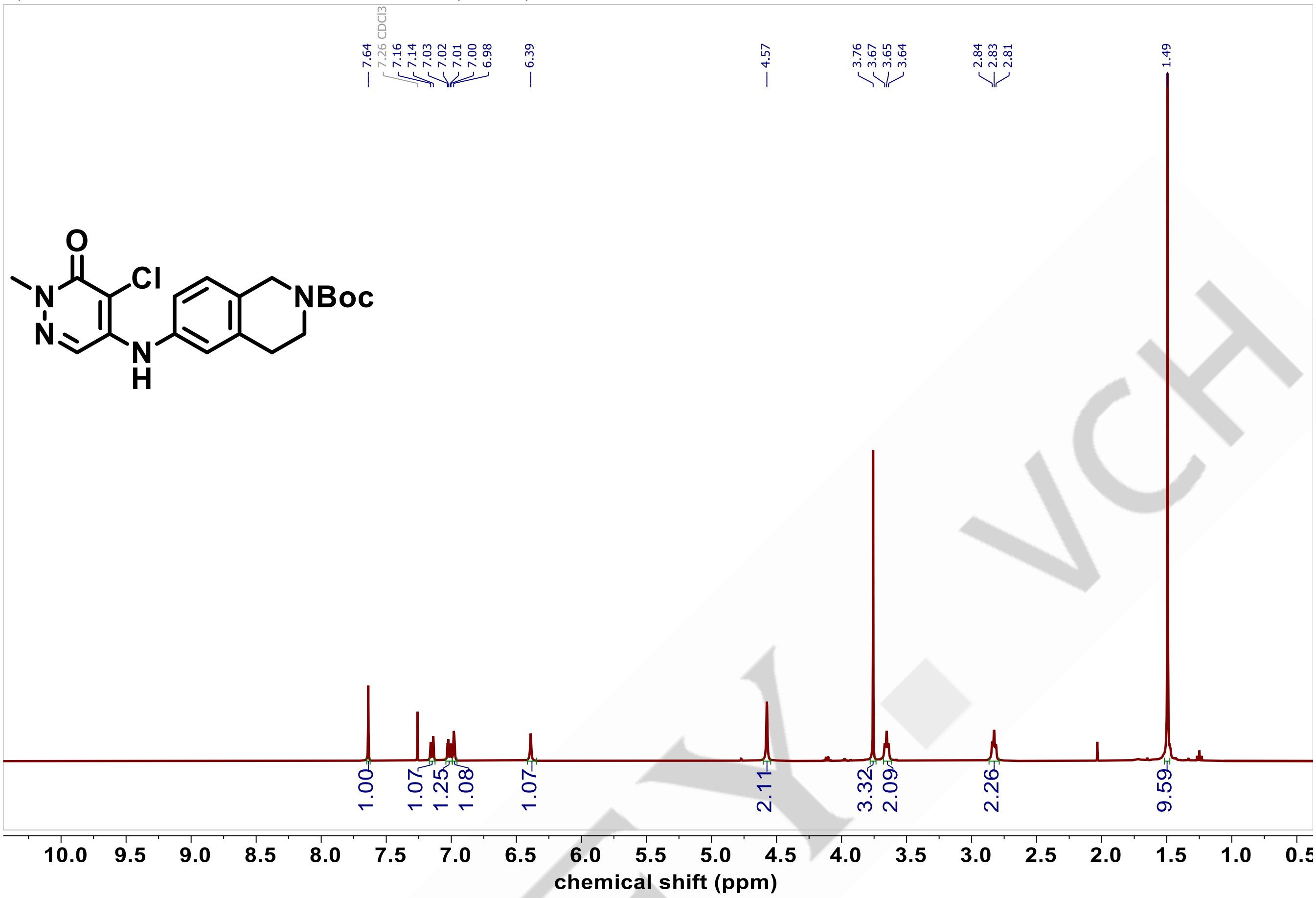

2, ^{1}H-NMR, 400 MHz, $CDCl_3$

wporre-230116-16.10.fid — RRE-1-96 — PROTON CDCl3 /opt/data wporre 16

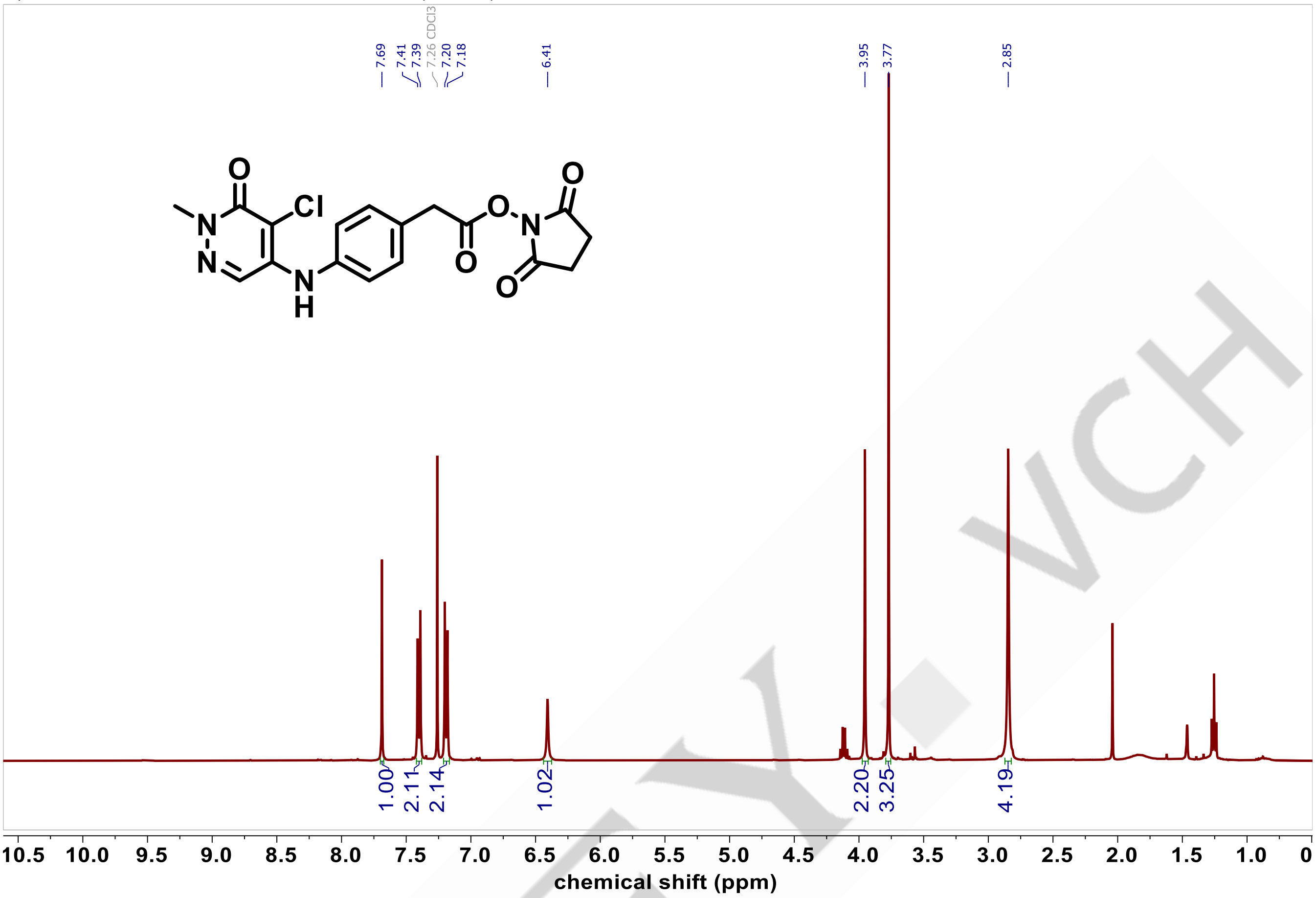

WILEY-VCH

2, ^{13}C-NMR, 101 MHz, $CDCl_3$

wporre-250414-60.11.fid — RRE-4-106 carbon — C13udeft CDCl3 /opt/data wporre 60

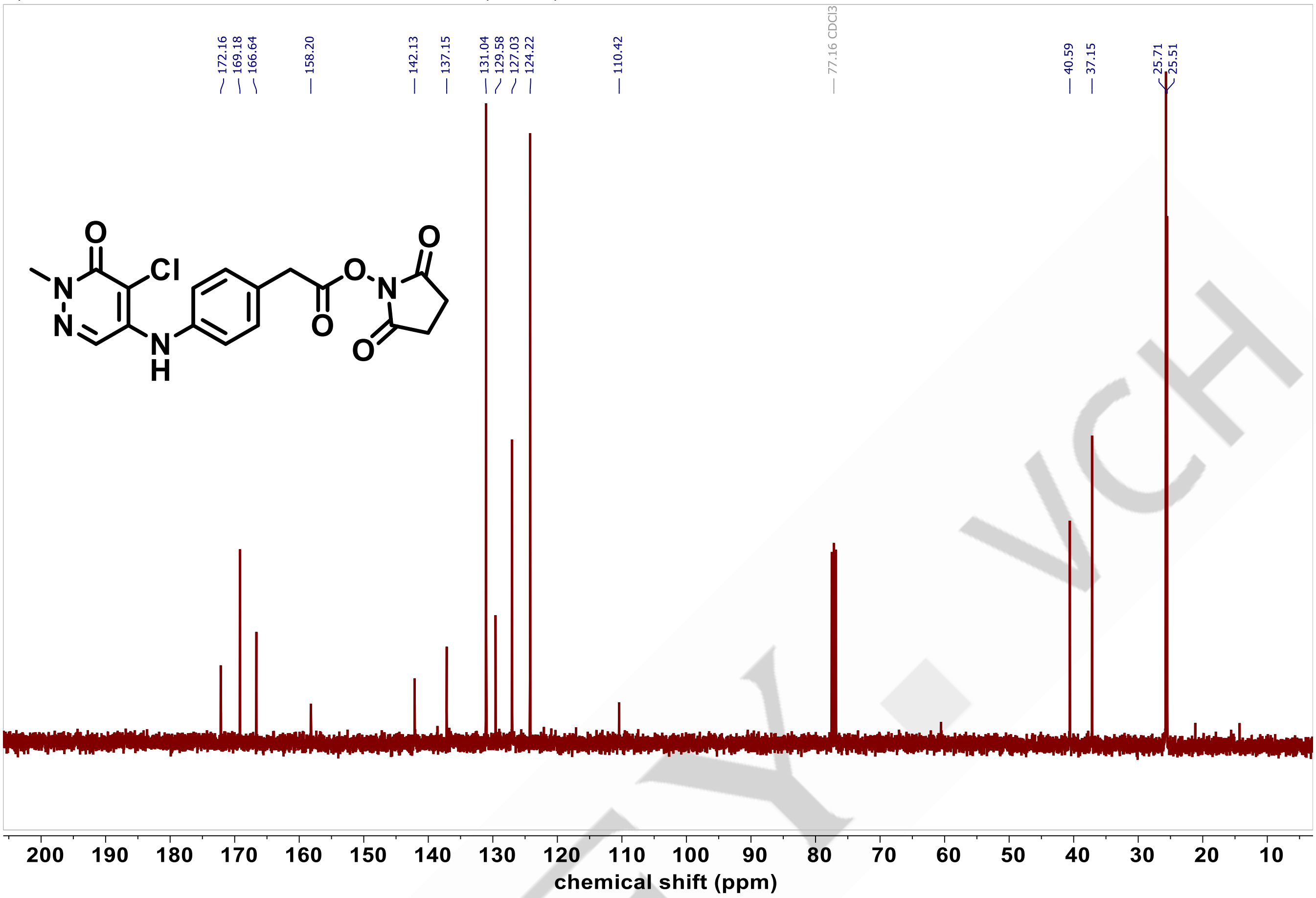

WILEY-VCH

WILEY-VCH

3, ^{1}H-NMR, 400 MHz, $CDCl_3$

wporre-250326-2.10.fid — RRE-1-72 proton new — PROTON CDCl3 /opt/data wporre 2

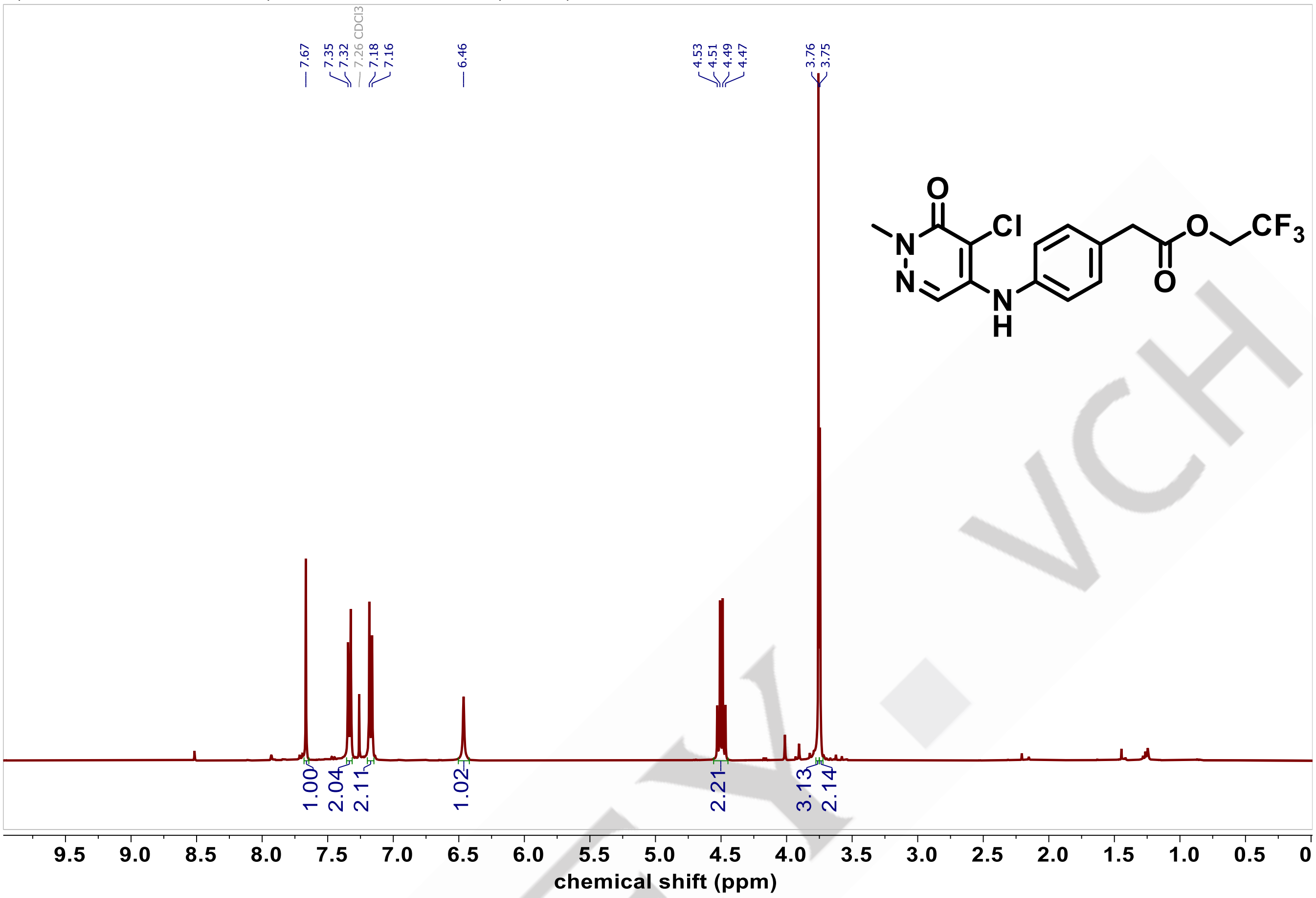

WILEY-VCH

3, ^{13}C-NMR, 101 MHz, $CDCl_3$

wporre-250326-2.11.fid — RRE-1-72 carbon new — C13udeft CDCl3 /opt/data wporre 2

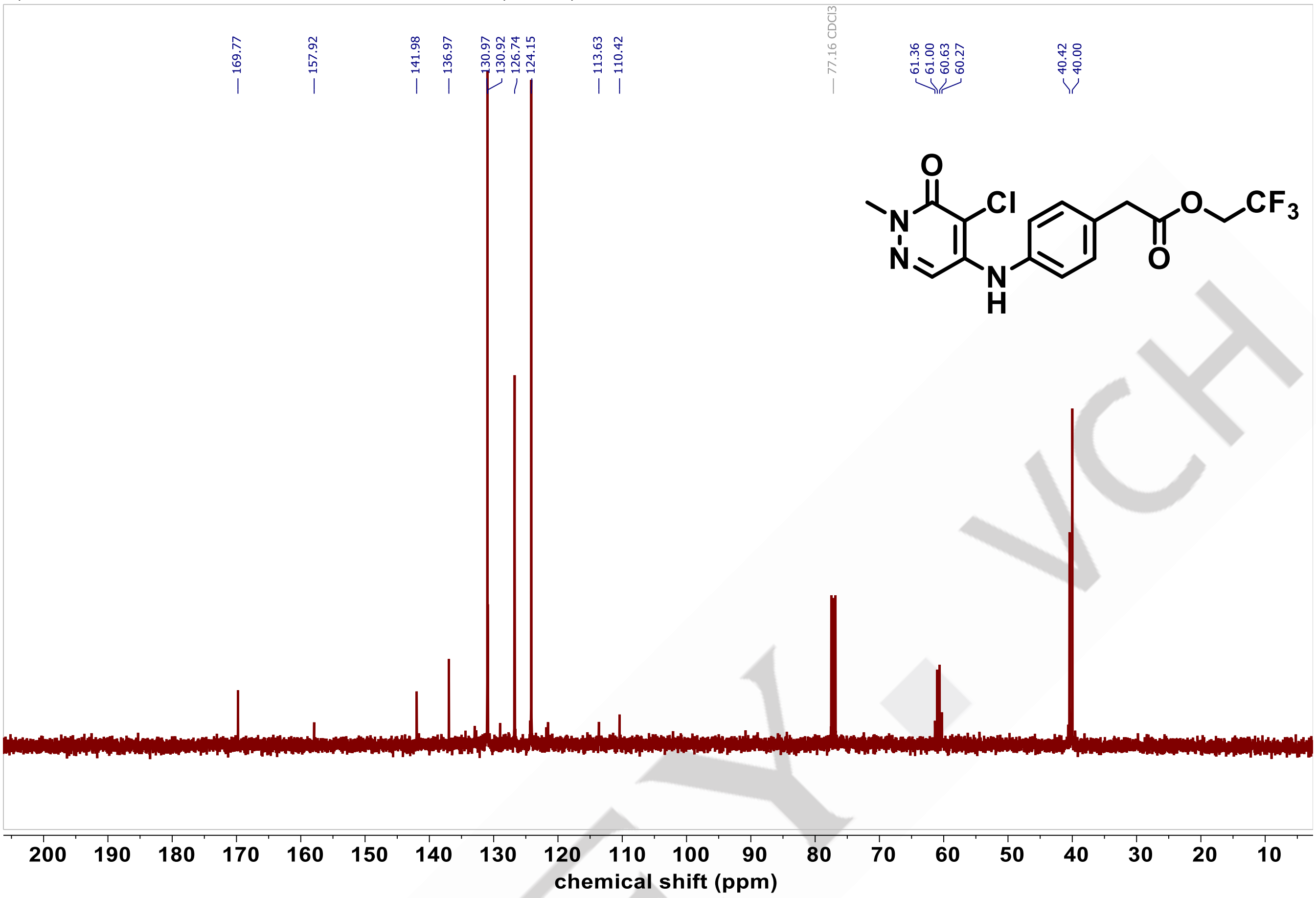

WILEY-VCH

3, ^{19}F-NMR, 376 MHz, CDCl$_3$

wporre-250326-2.13.fid — RRE-1-72 19F with decpln — F19CPD CDCl3 /opt/data wporre 2

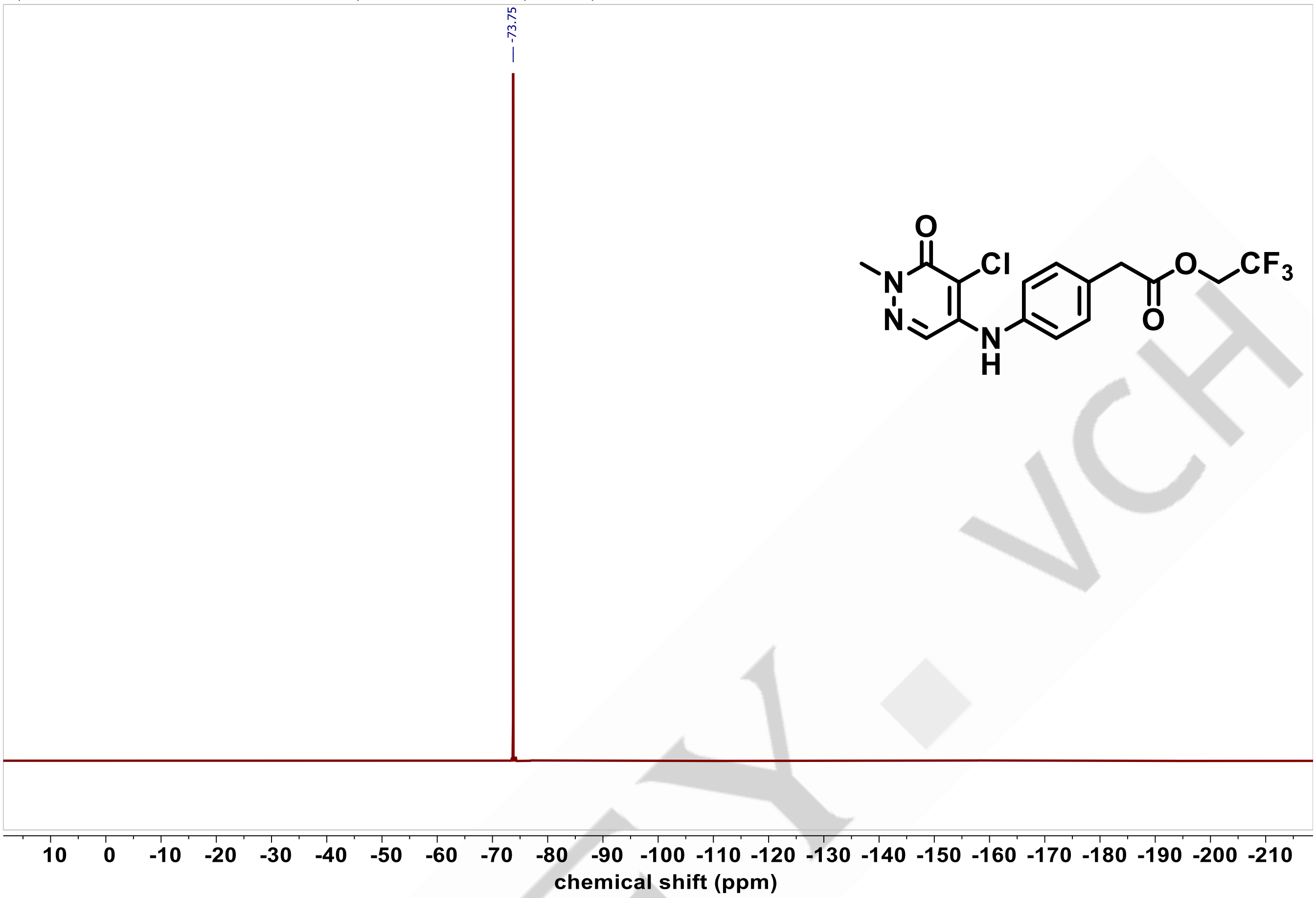

WILEY-VCH

4, ¹H-NMR, 400 MHz, $CDCl_3$

wporre-250417-8.10.fid — RRE-4-108 proton — PROTON CDCl3 /opt/data wporre 8

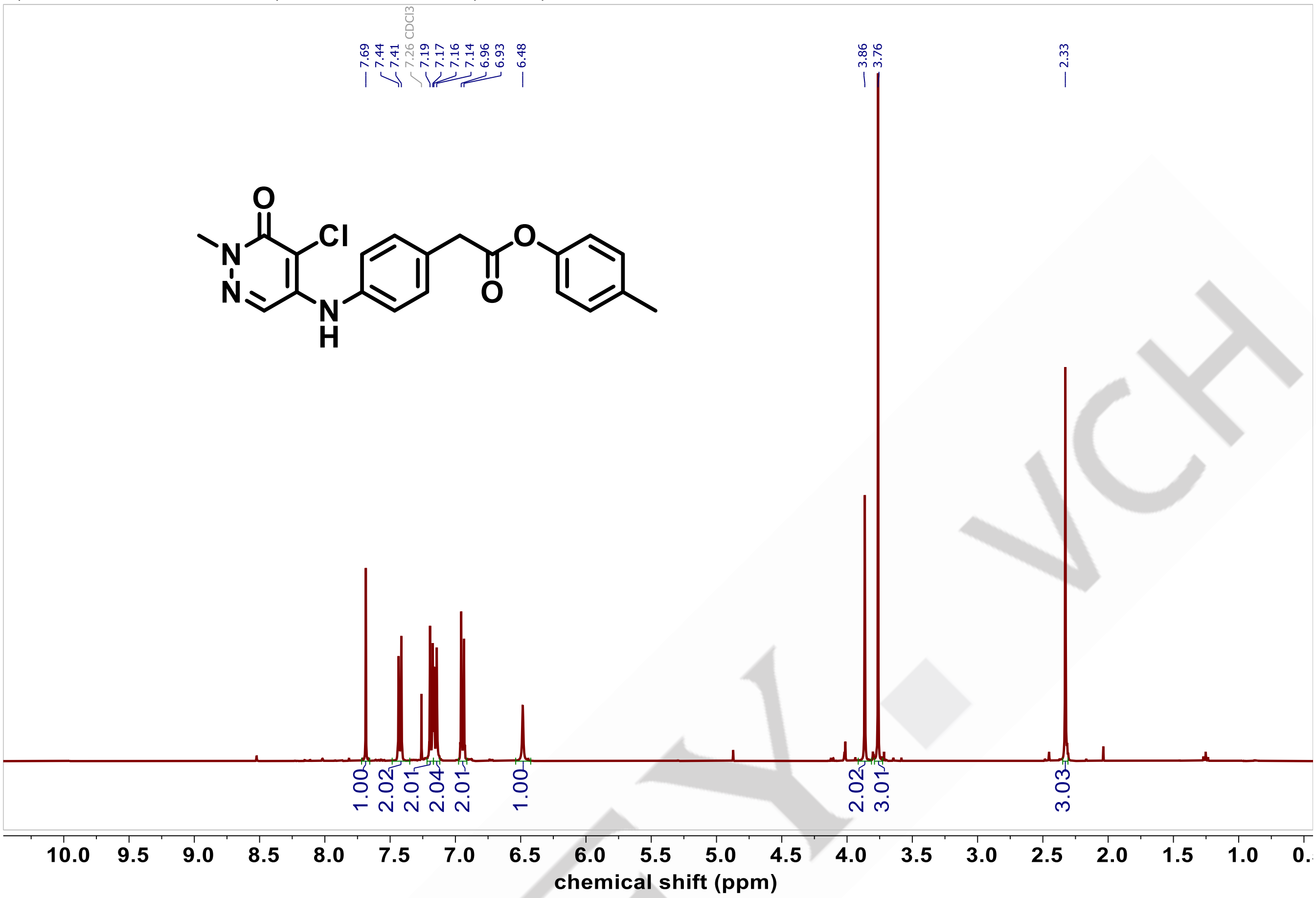

WILEY-VCH

4, ^{13}C-NMR, 101 MHz, $CDCl_3$

wporre-250417-8.11.fid — RRE-4-108 carbon — C13udeft CDCl3 /opt/data wporre 8

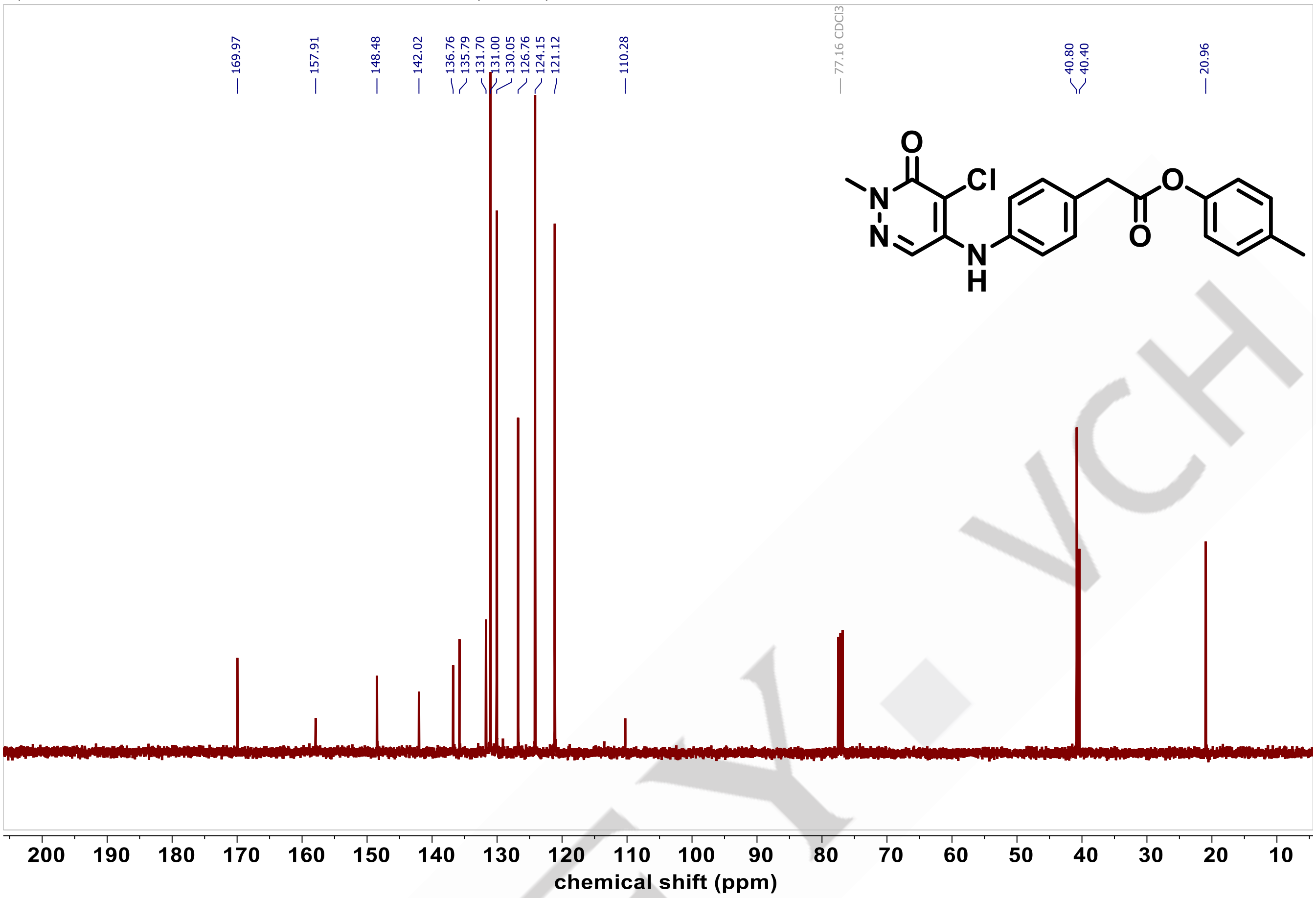

WILEY-VCH

5, ^{1}H-NMR, 400 MHz, $CDCl_3$

wporre-240909-18.10.fid — RRE-1-108 — PROTON CDCl3 /opt/data wporre 18

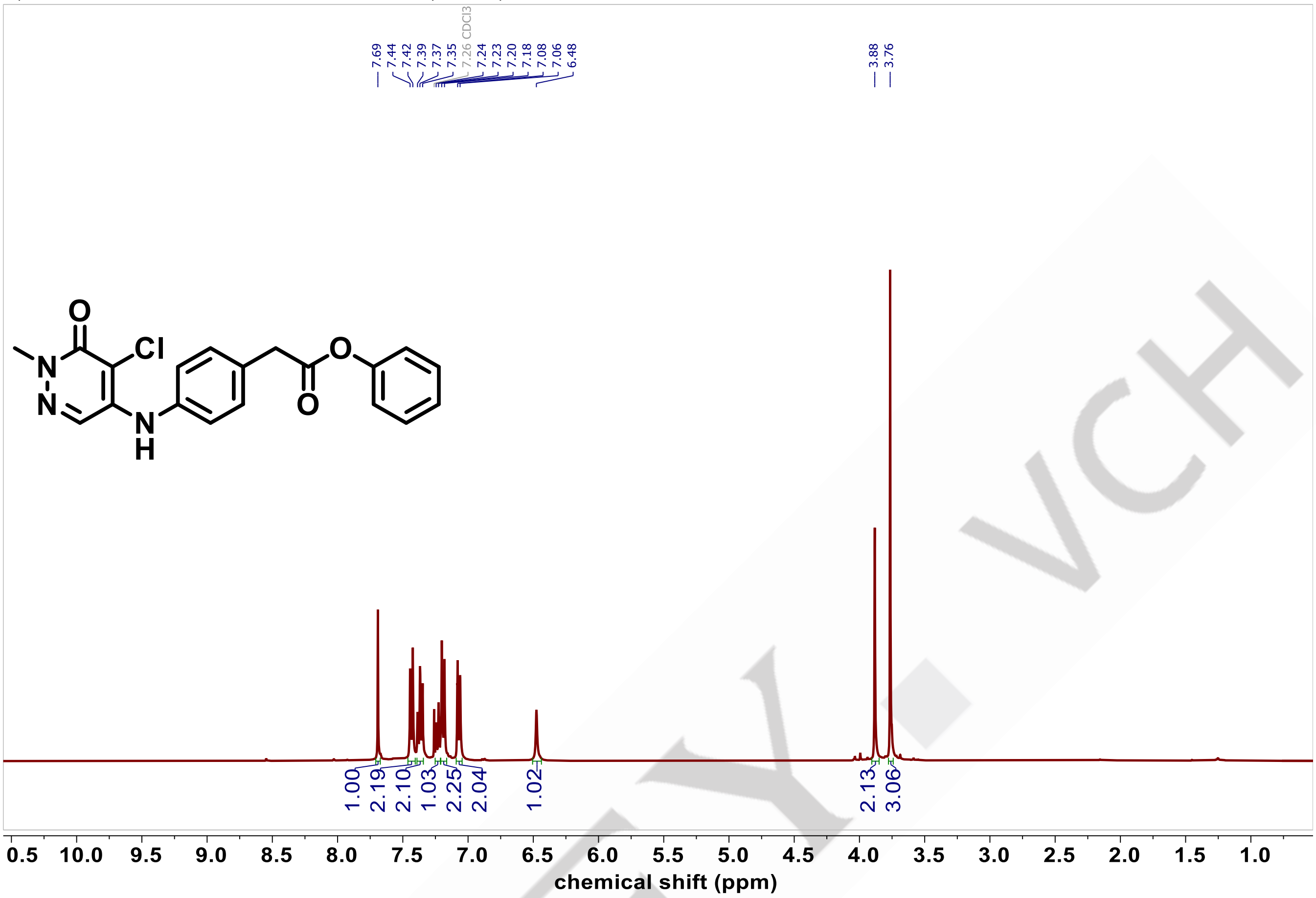

WILEY-VCH

5, ^{13}C-NMR, 101 MHz, $CDCl_3$

wporre-240909-31.10.fid — RRE.1.108 Dry carbon — C13CPD_128 CDCl3 /opt/data wporre 31

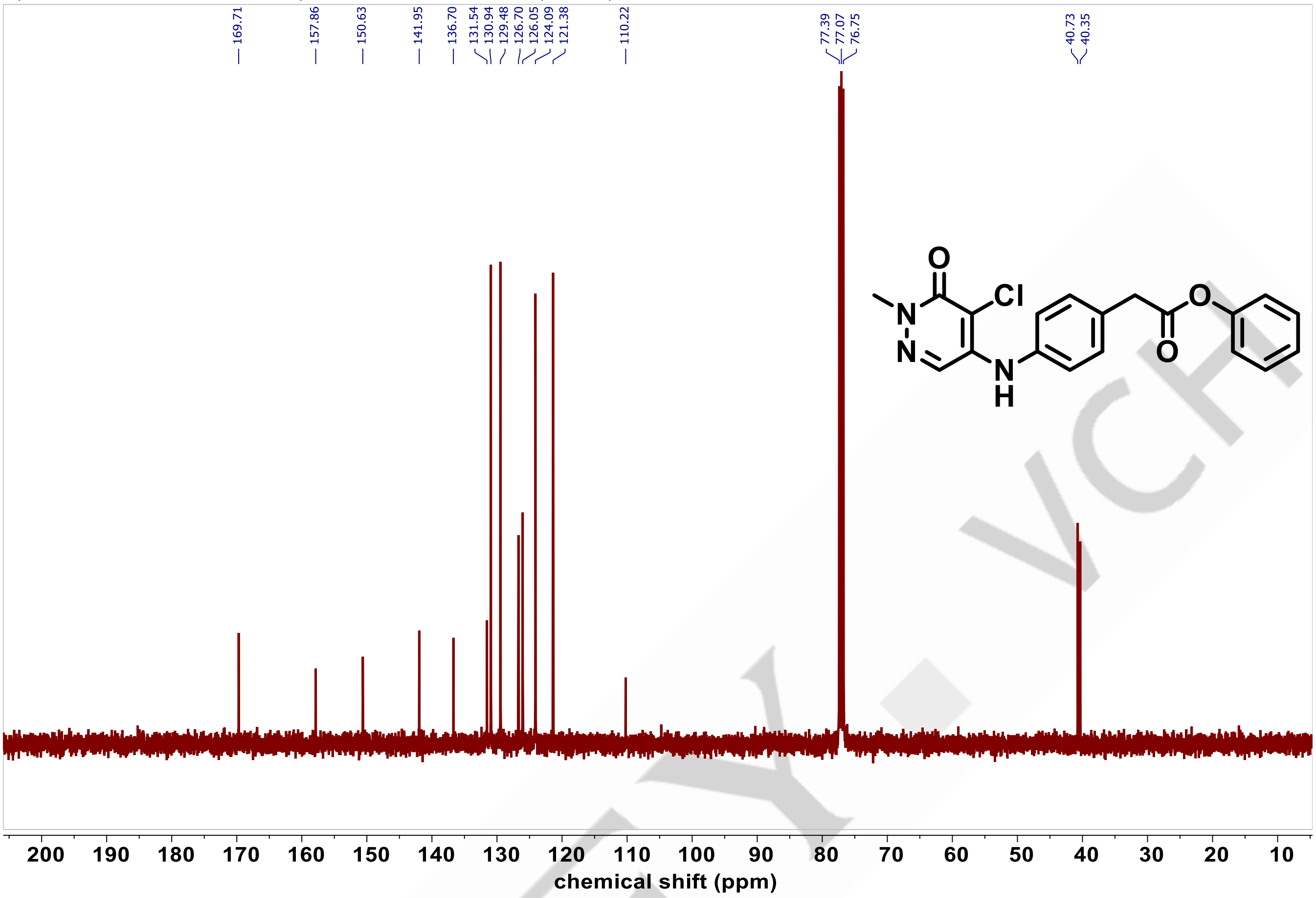

6, ^{1}H-NMR, 400 MHz, $CDCl_3$

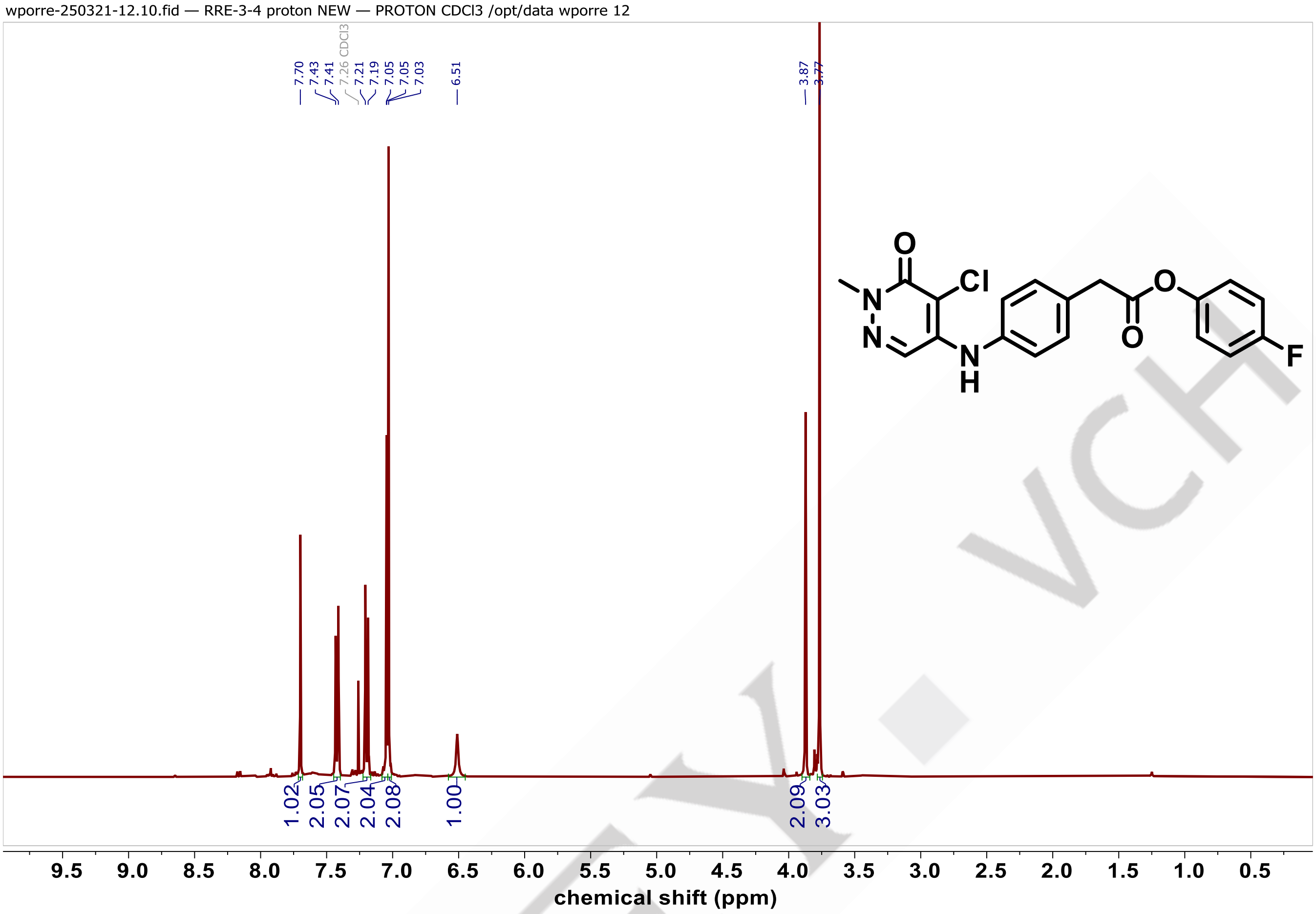

6, ^{13}C-NMR, 101 MHz, $CDCl_3$

wporre-251016-34.11.fid — RRE-3-4 repur. 13C — C13udeft CDCl3 /opt/data wporre 34

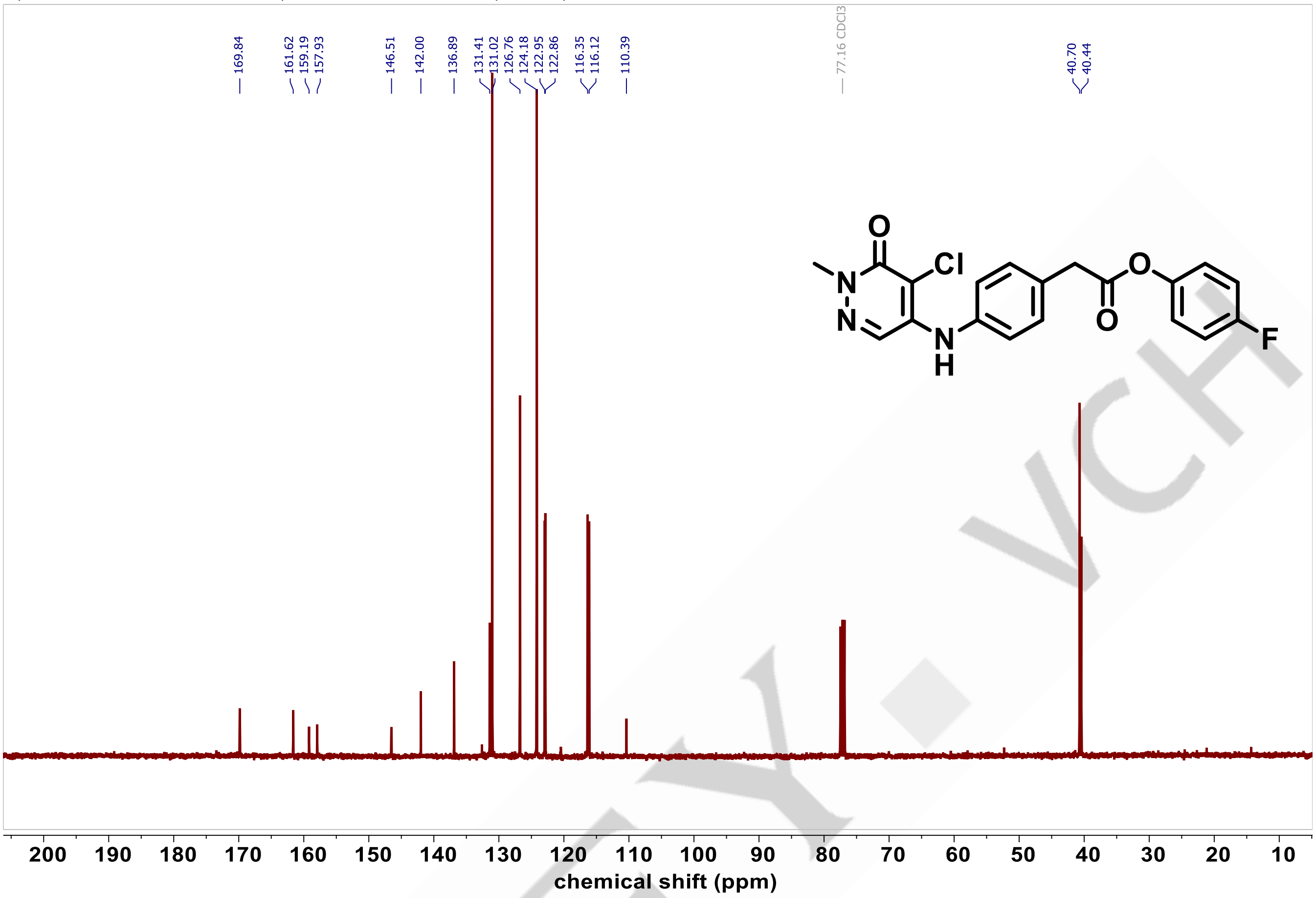

WILEY-VCH

6, ^{19}F-NMR, 376 MHz, $CDCl_3$

wporre-251016-34.12.fid — RRE-3-4 repur. 19F with dcpln — F19CPD CDCl3 /opt/data wporre 34

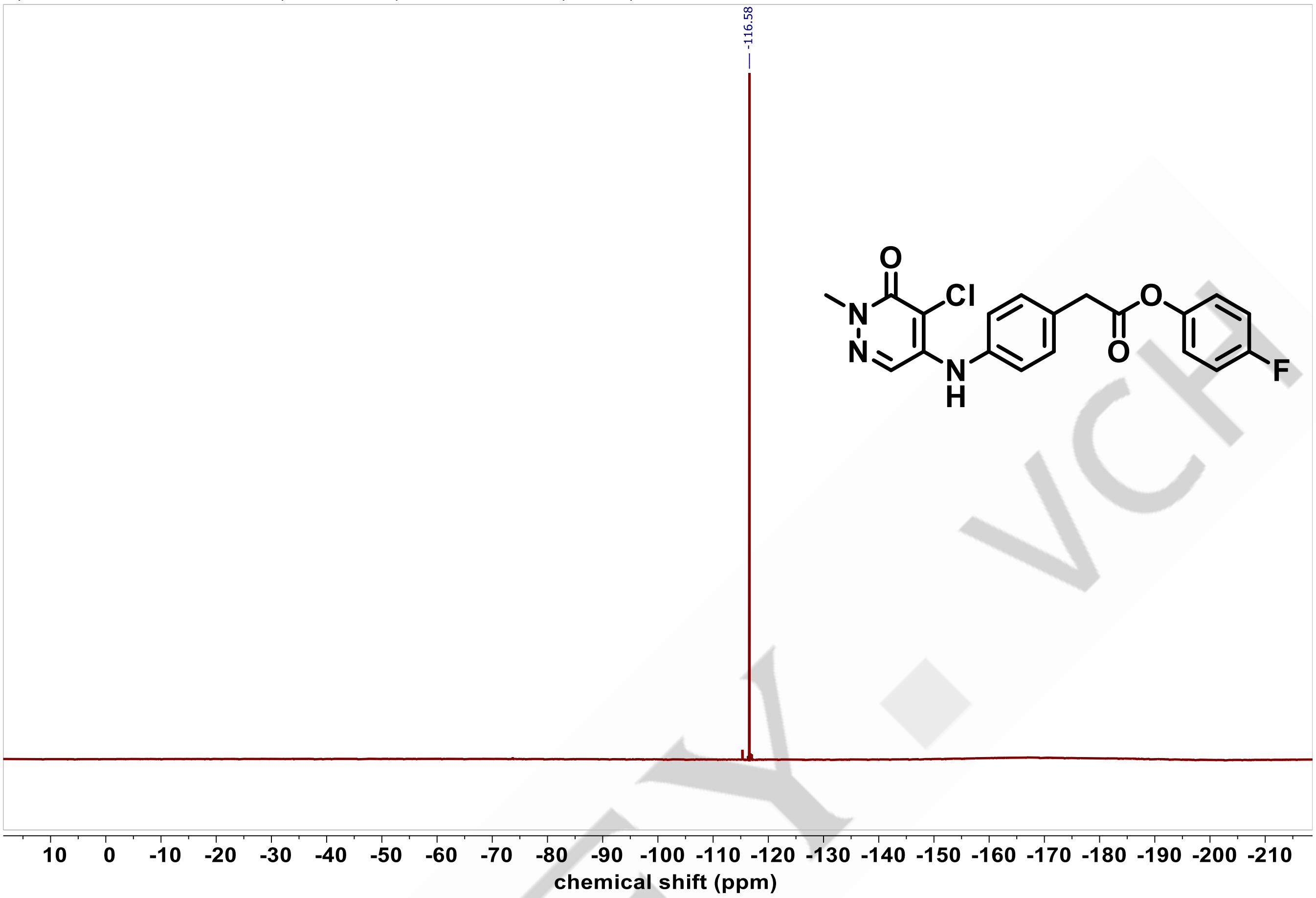

WILEY-VCH

7, ^{1}H-NMR, 400 MHz, $CDCl_3$

wporre-251030-53.10.fid — RRE-4-134 AGAIN — PROTON DMSO /opt/data wporre 53

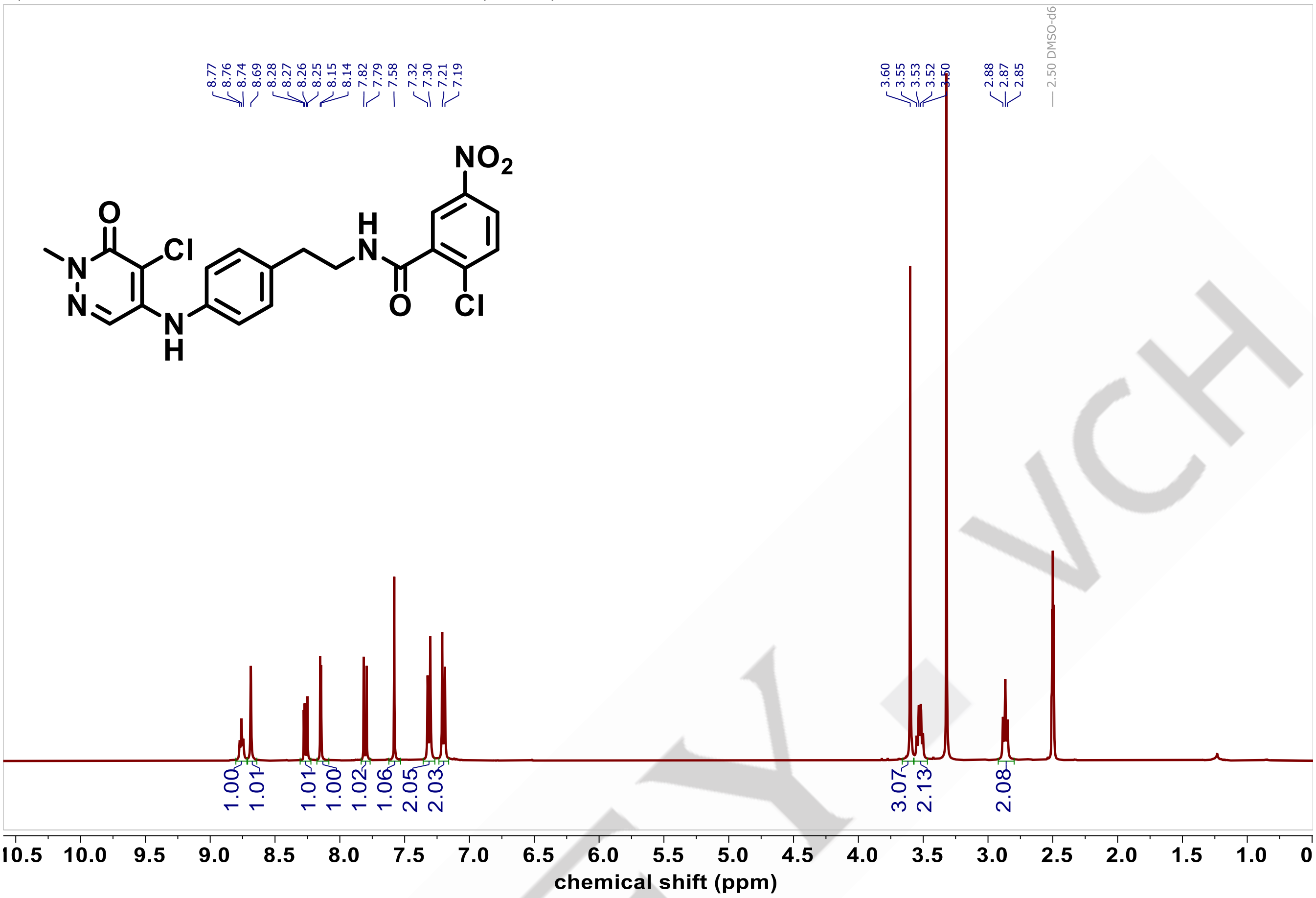

WILEY VCH

7, ^{13}C-NMR, 101 MHz, $CDCl_3$

wporre-251030-53.11.fid — RRE-4-134 AGAIN — C13udeft DMSO /opt/data wporre 53

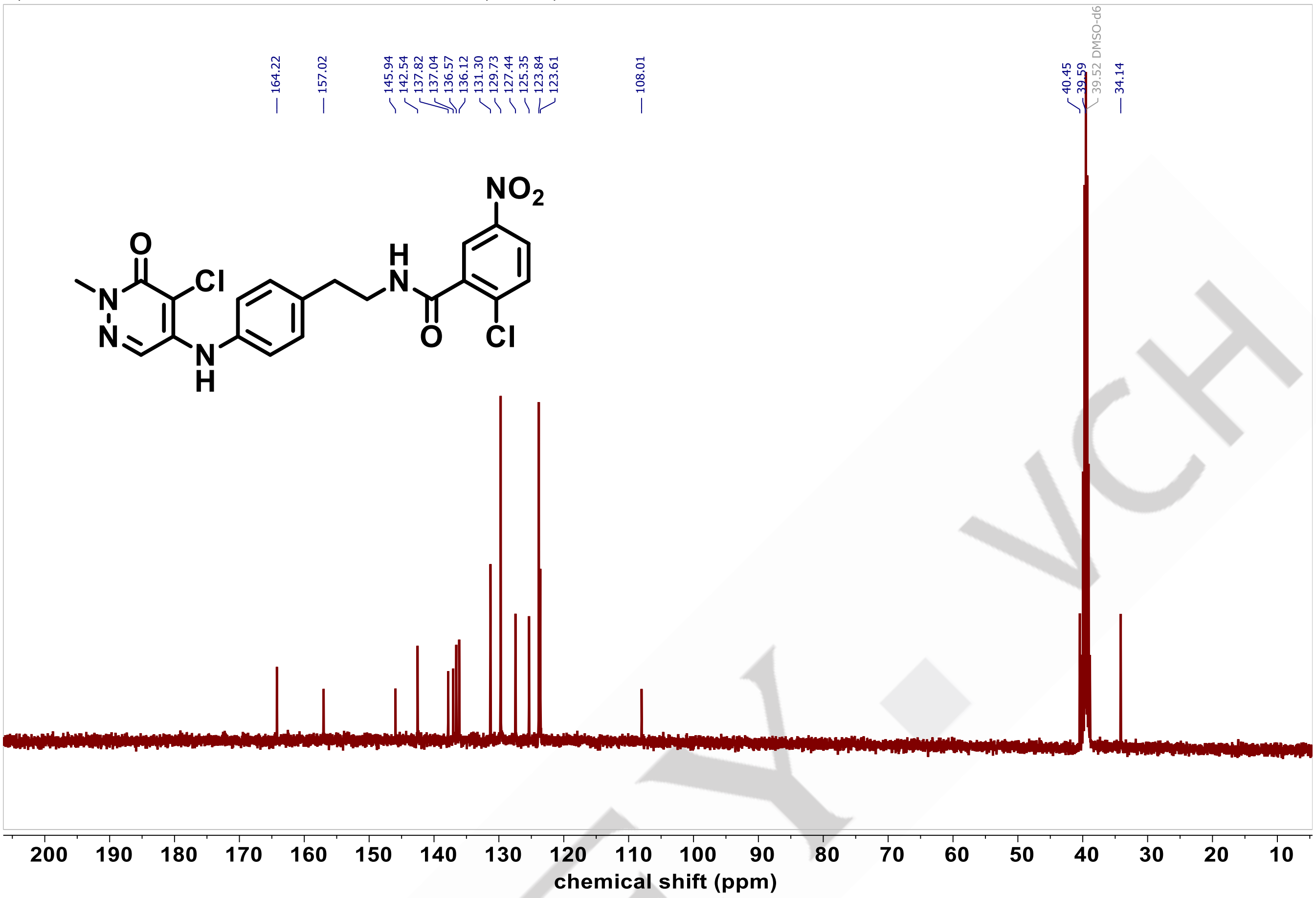

WILEY-VCH

8, ^{1}H-NMR, 400 MHz, $CDCl_3$

wporre-250326-5.10.fid — RRE-3-114 proton new2 — PROTON CDCl3 /opt/data wporre 5

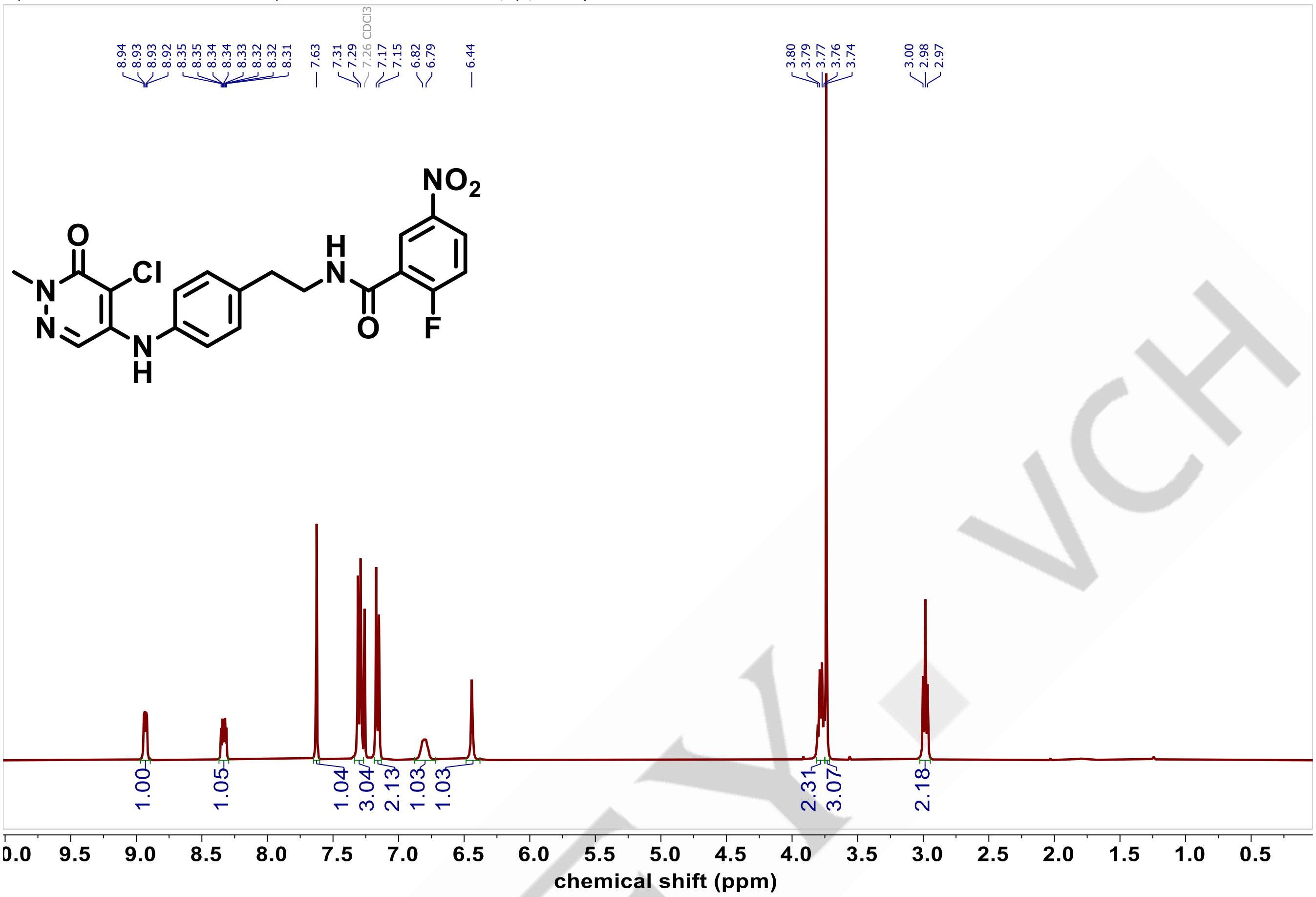

WILEY-VCH

8, ^{13}C-NMR, 101 MHz, $CDCl_3$

wporre-251009-15.11.fid — RRE-3-114 FALL25 — C13udeft CDCl3 /opt/data wporre 15

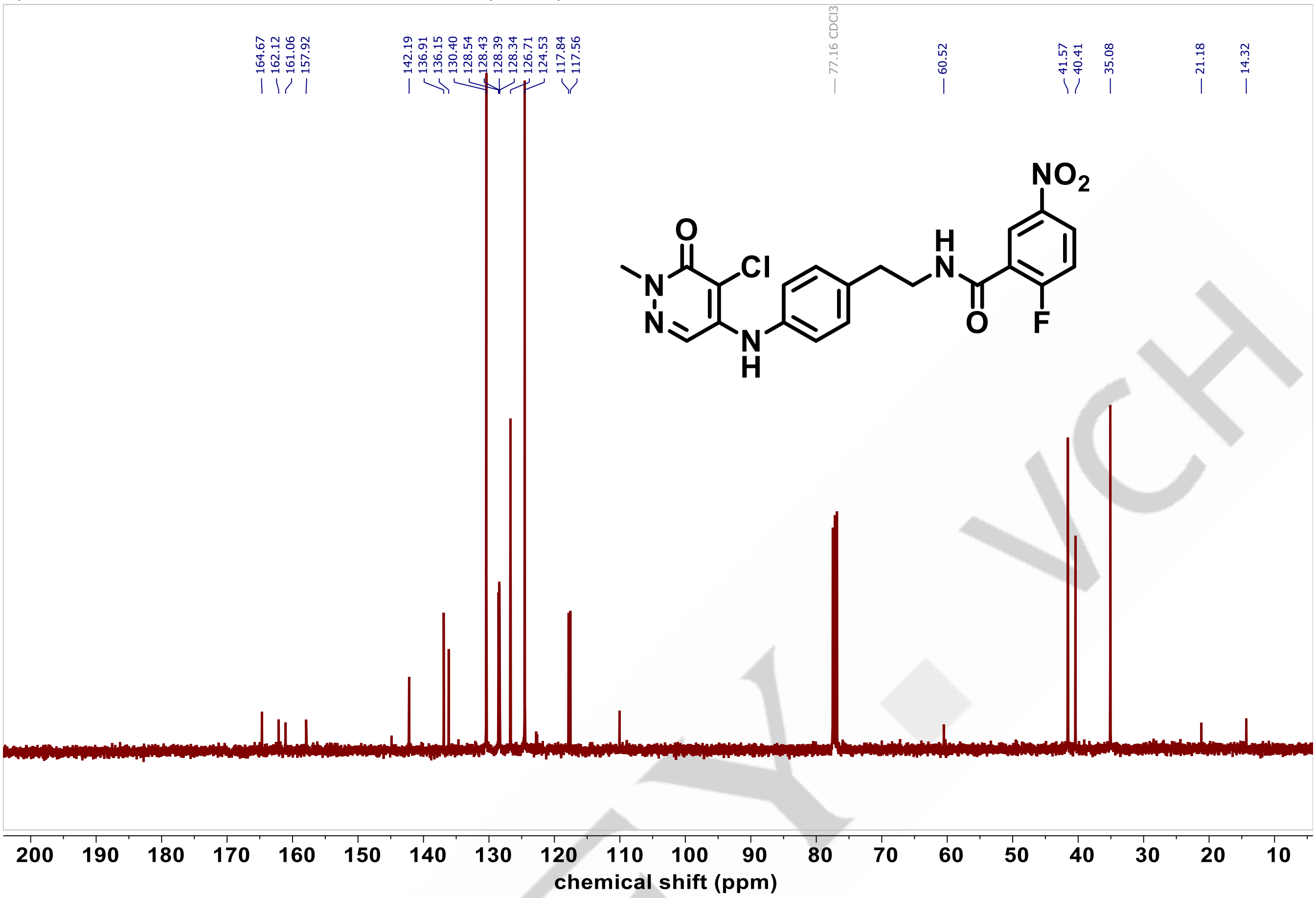

WILEY-VCH

8, ^{19}F-NMR, 376 MHz, $CDCl_3$

wporre-251009-15.10.fid — RRE-3-114 FALL25 19F with decpln — F19CPD CDCl3 /opt/data wporre 15

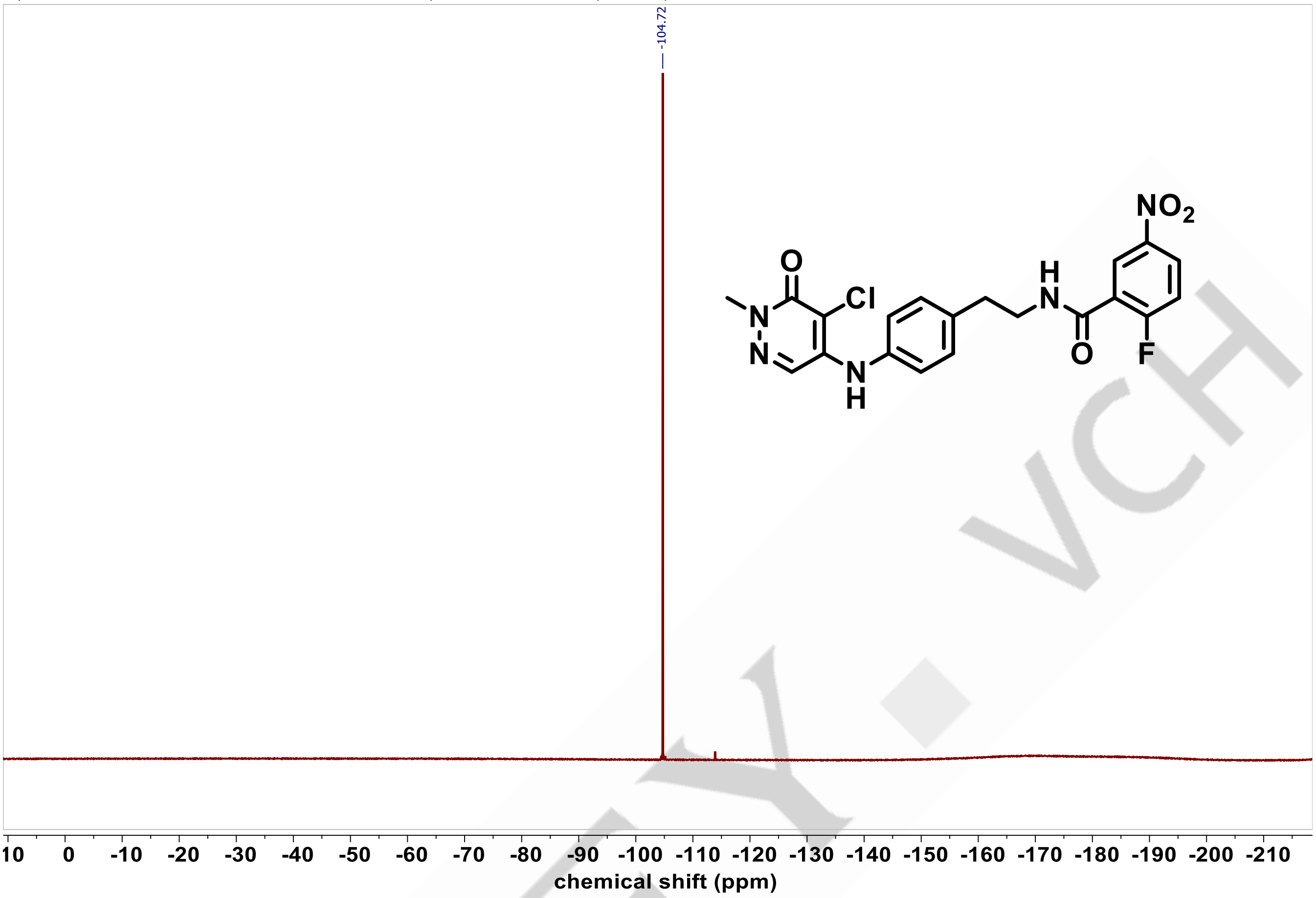

9, ^{1}H-NMR, 400 MHz, $CDCl_3$

wporre-250326-6.10.fid — RRE-3-146 proton new2 — PROTON CDCl3 /opt/data wporre 6

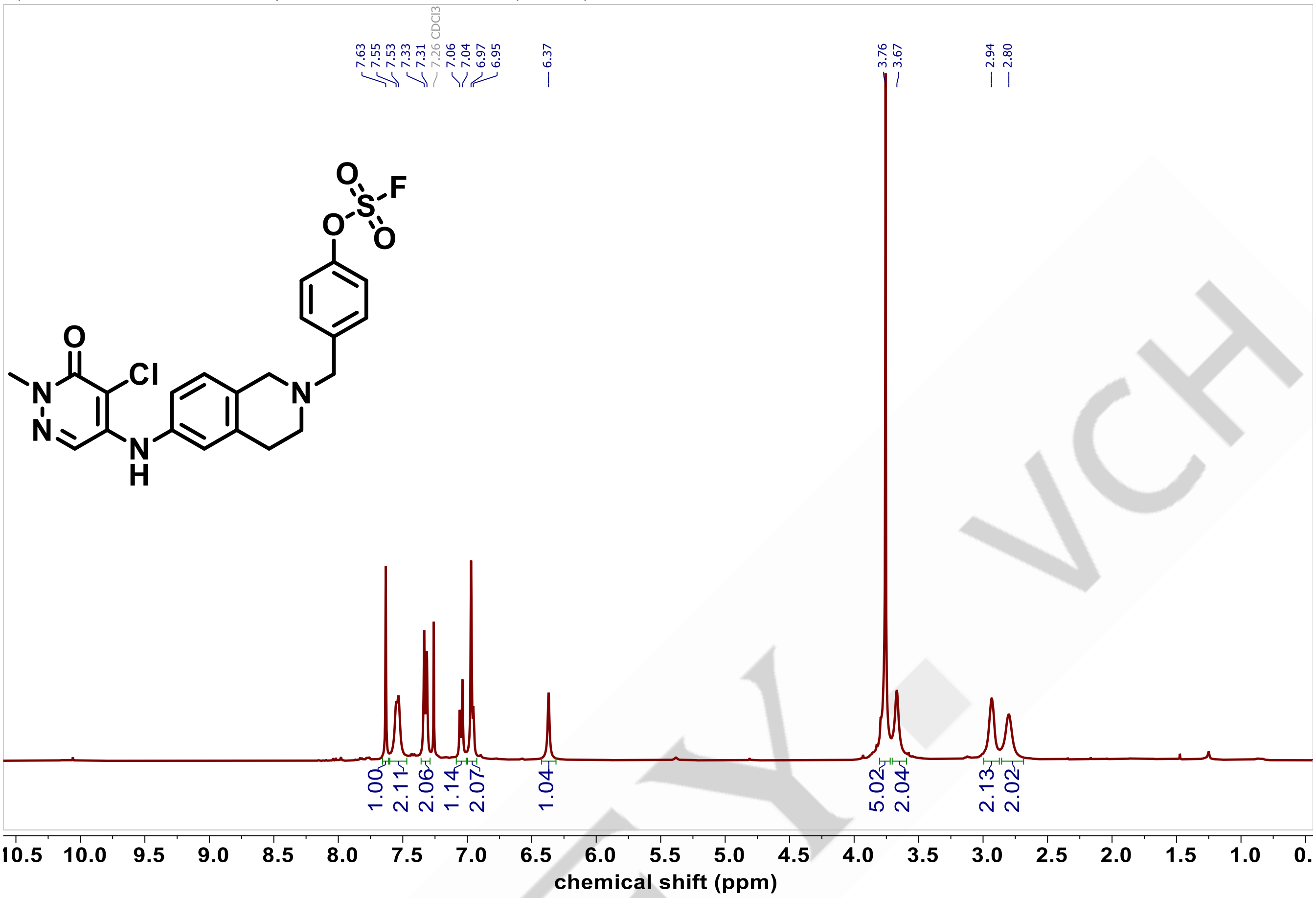

9, ^{13}C-NMR, 101 MHz, $CDCl_3$

wporre-251201-58.13.fid — RRE-5-58 13C 1024 scans — C13udeft CDCl3 /opt/data wporre 58

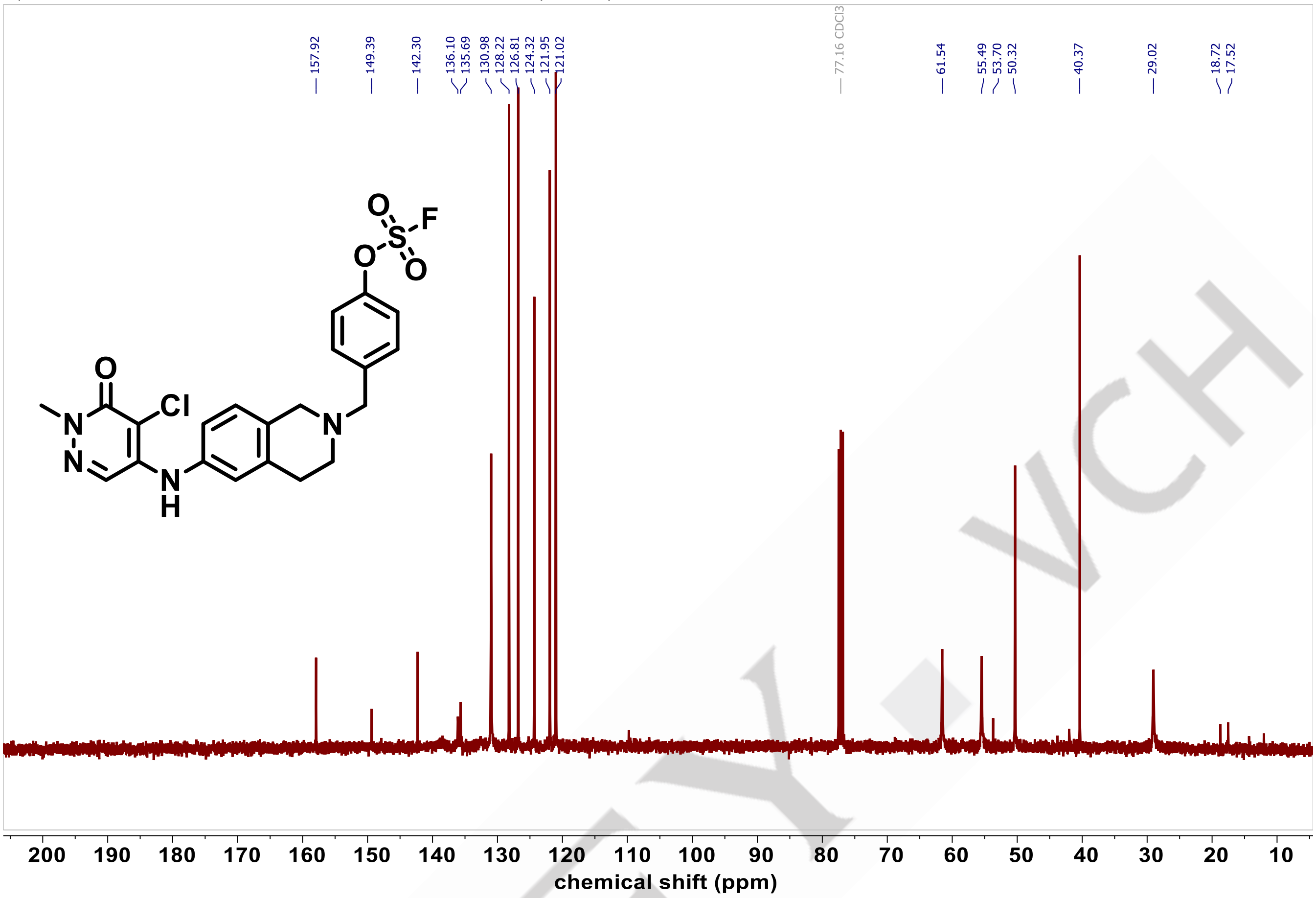

9, ^{19}F-NMR, 376 MHz, $CDCl_3$

wporre-251201-58.11.fid — RRE-5-58 19F with decpln — F19CPD CDCl3 /opt/data wporre 58

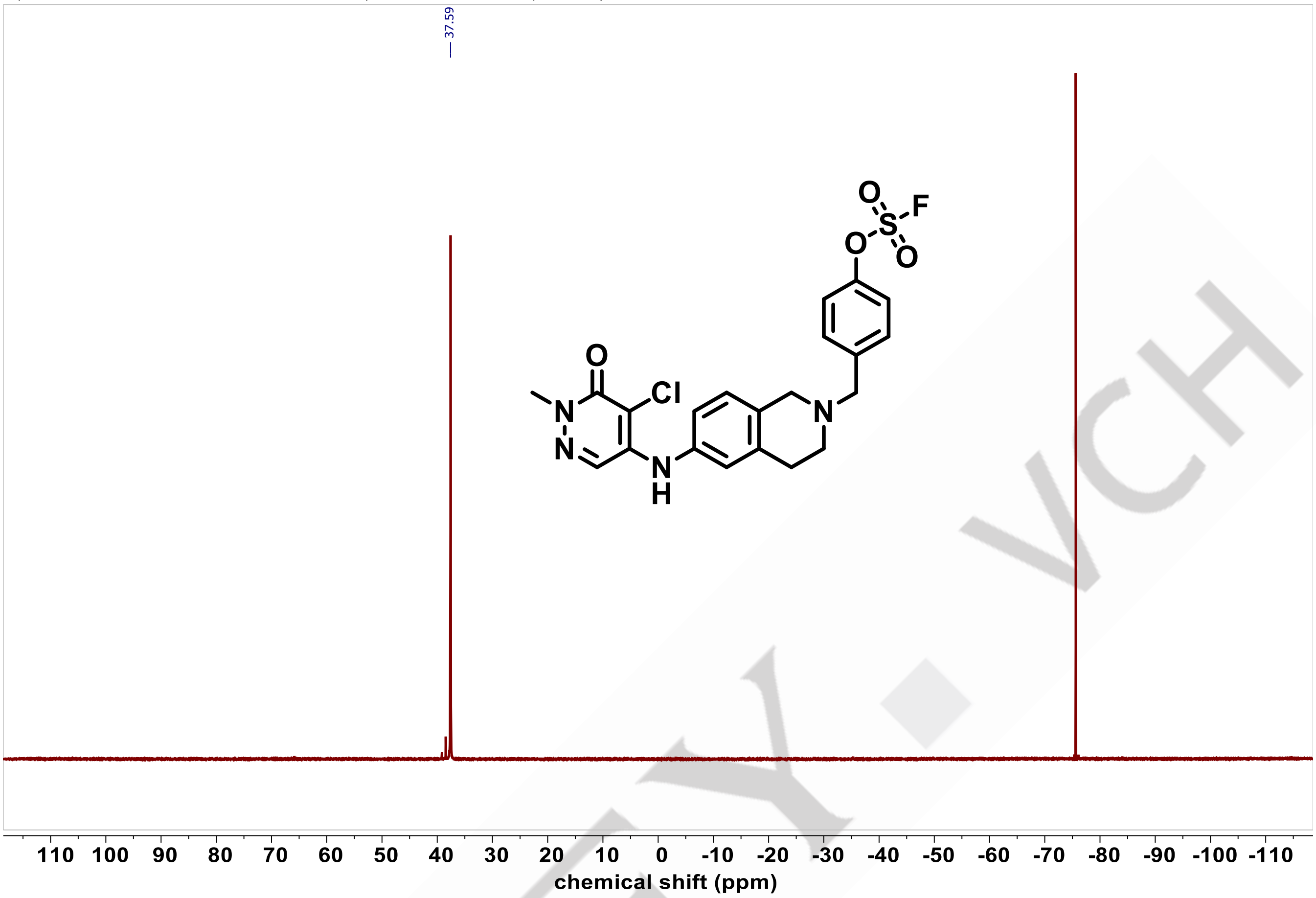

WILEY-VCH

10, ¹H-NMR, 400 MHz, CDCl₃

wporre-250115-29.10.fid — RRE.3.100 proton "DRY" — PROTON CDCl3 /opt/data wporre 29

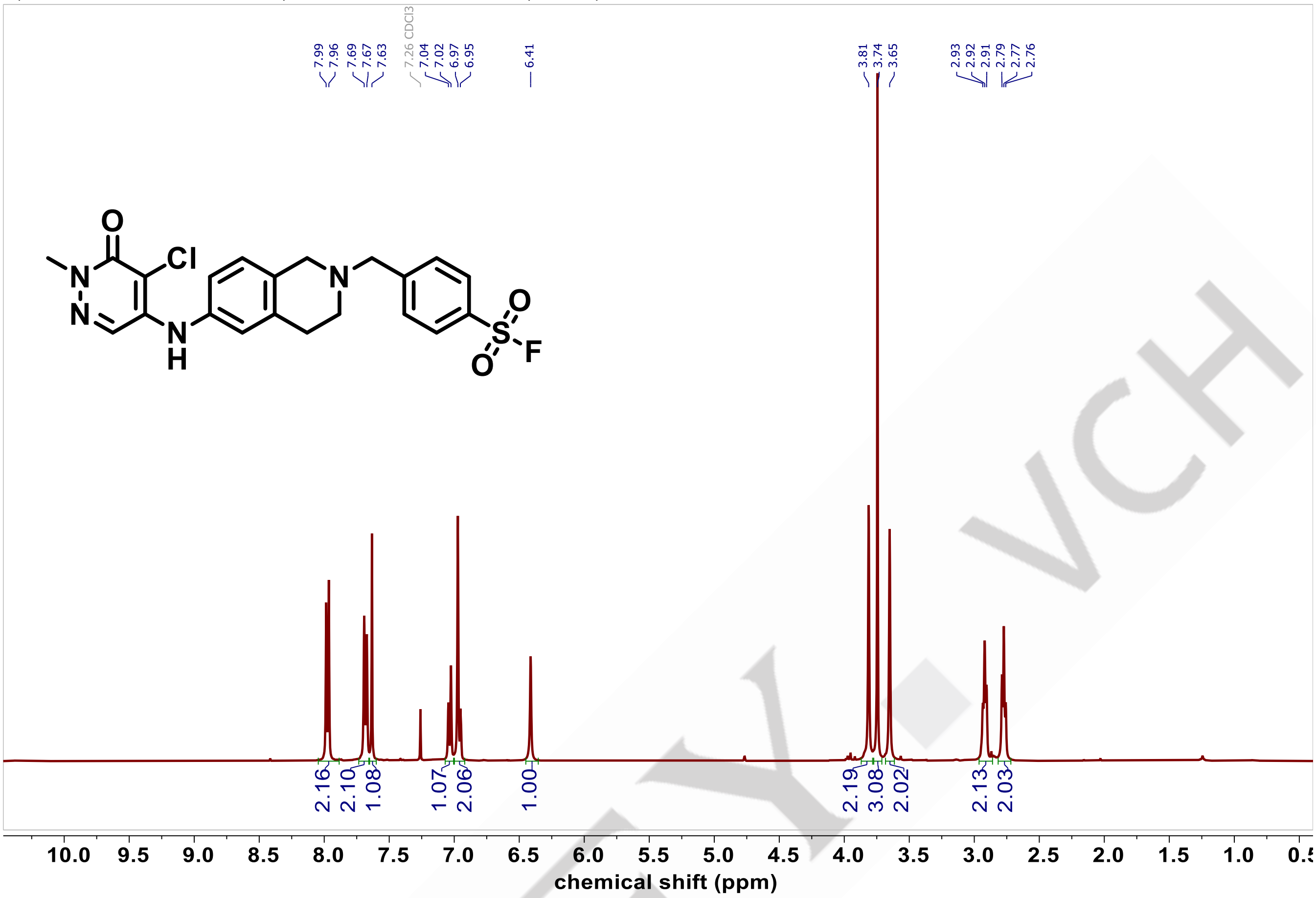

WILEY-VCH

10, ^{13}C-NMR, 101 MHz, $CDCl_3$

wporre-250115-29.11.fid — RRE.3.100 carbon "DRY" — C13udeft CDCl3 /opt/data wporre 29

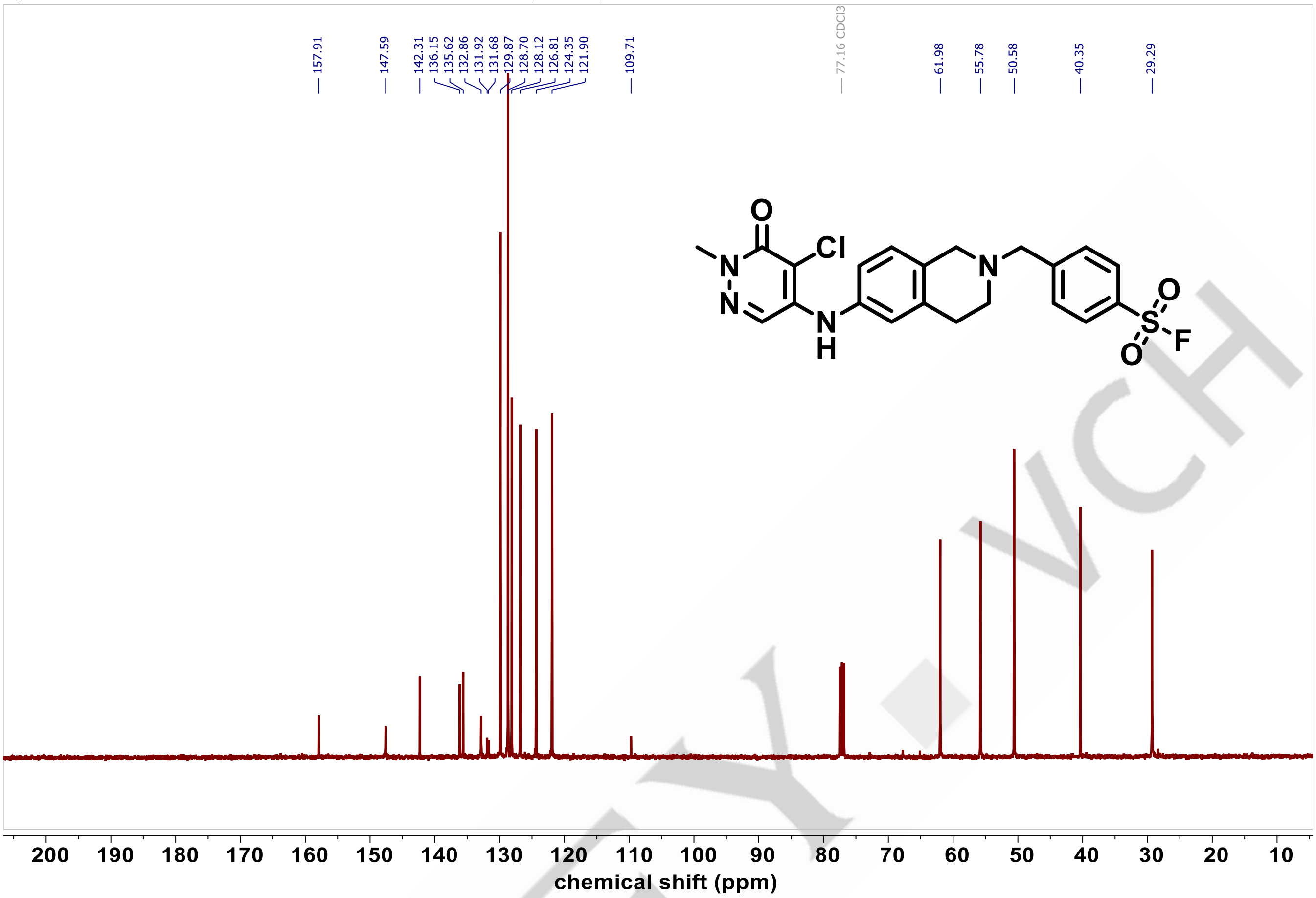

WILEY-VCH

10, ^{19}F-NMR, 376 MHz, $CDCl_3$

wporre-250115-29.13.fid — RRE.3.100 19F with decpln — F19CPD CDCl3 /opt/data wporre 29

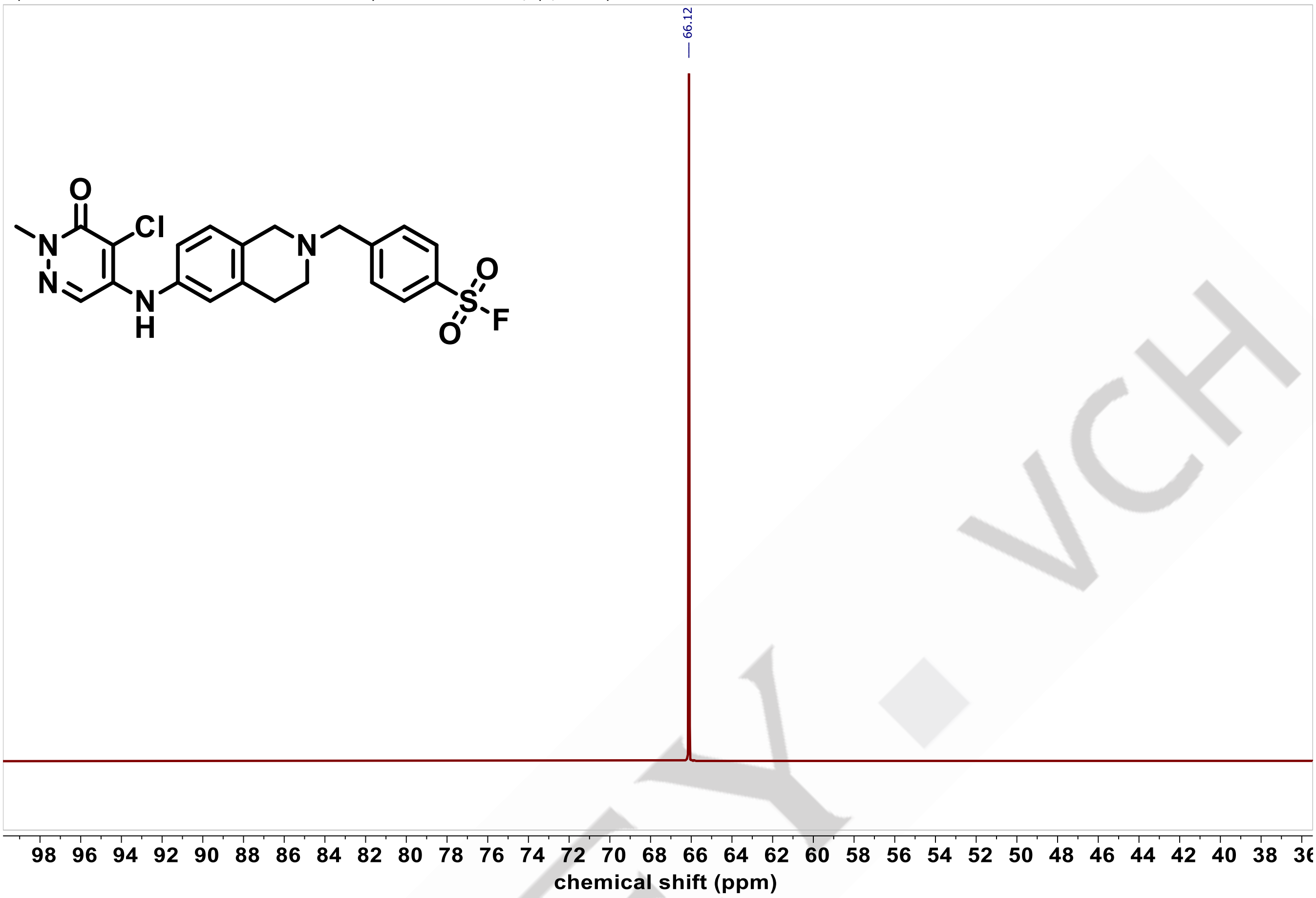

11, ^{1}H-NMR, 400 MHz, $CDCl_3$

wporre-250321-40.10.fid — RRE-3-136 proton new — PROTON CDCl3 /opt/data wporre 40

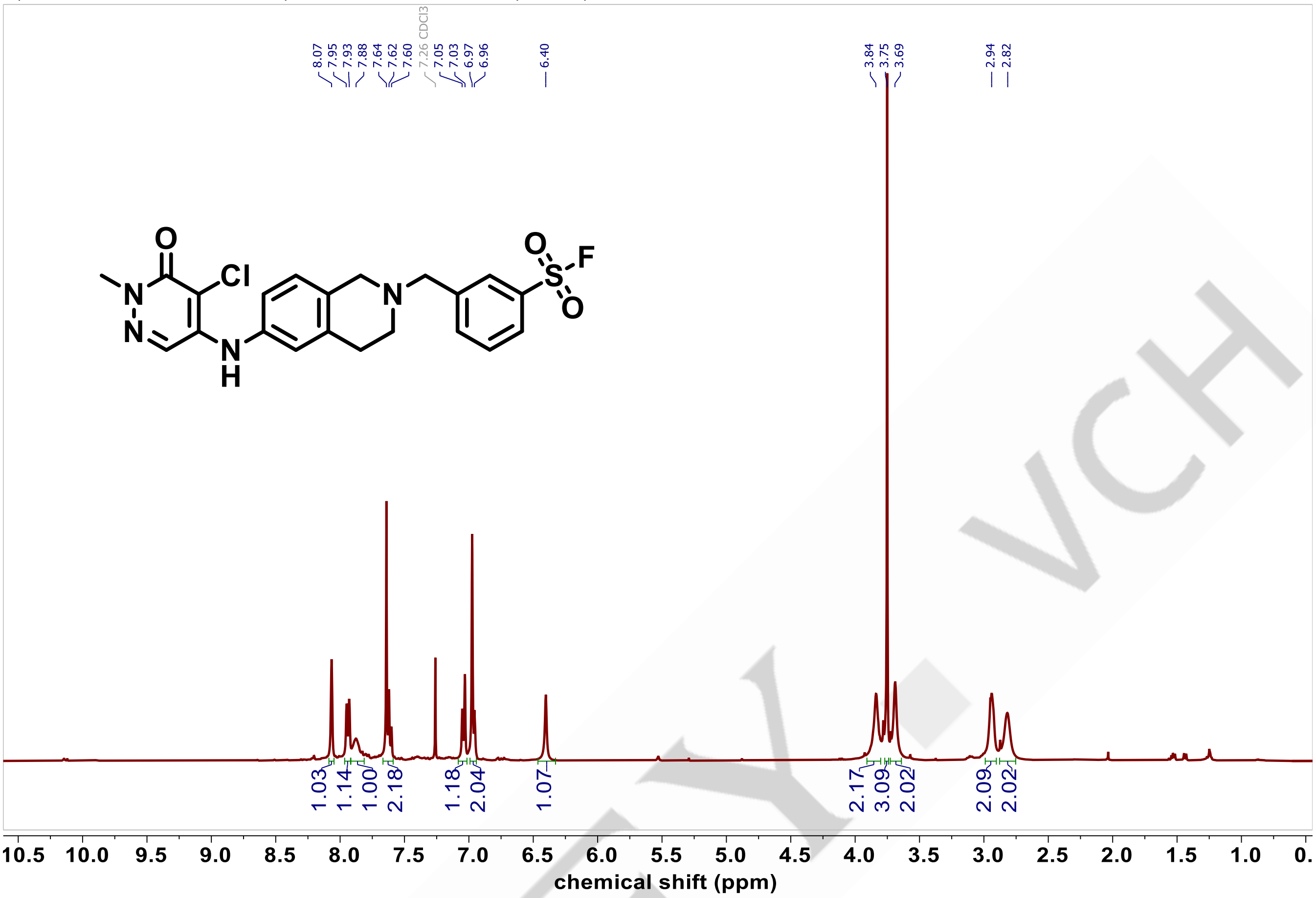

WILEY-VCH

11, ^{13}C-NMR, 101 MHz, $CDCl_3$

wporre-251201-56.11.fid — RRE-5-56 "DRY" — C13udeft CDCl3 /opt/data wporre 56

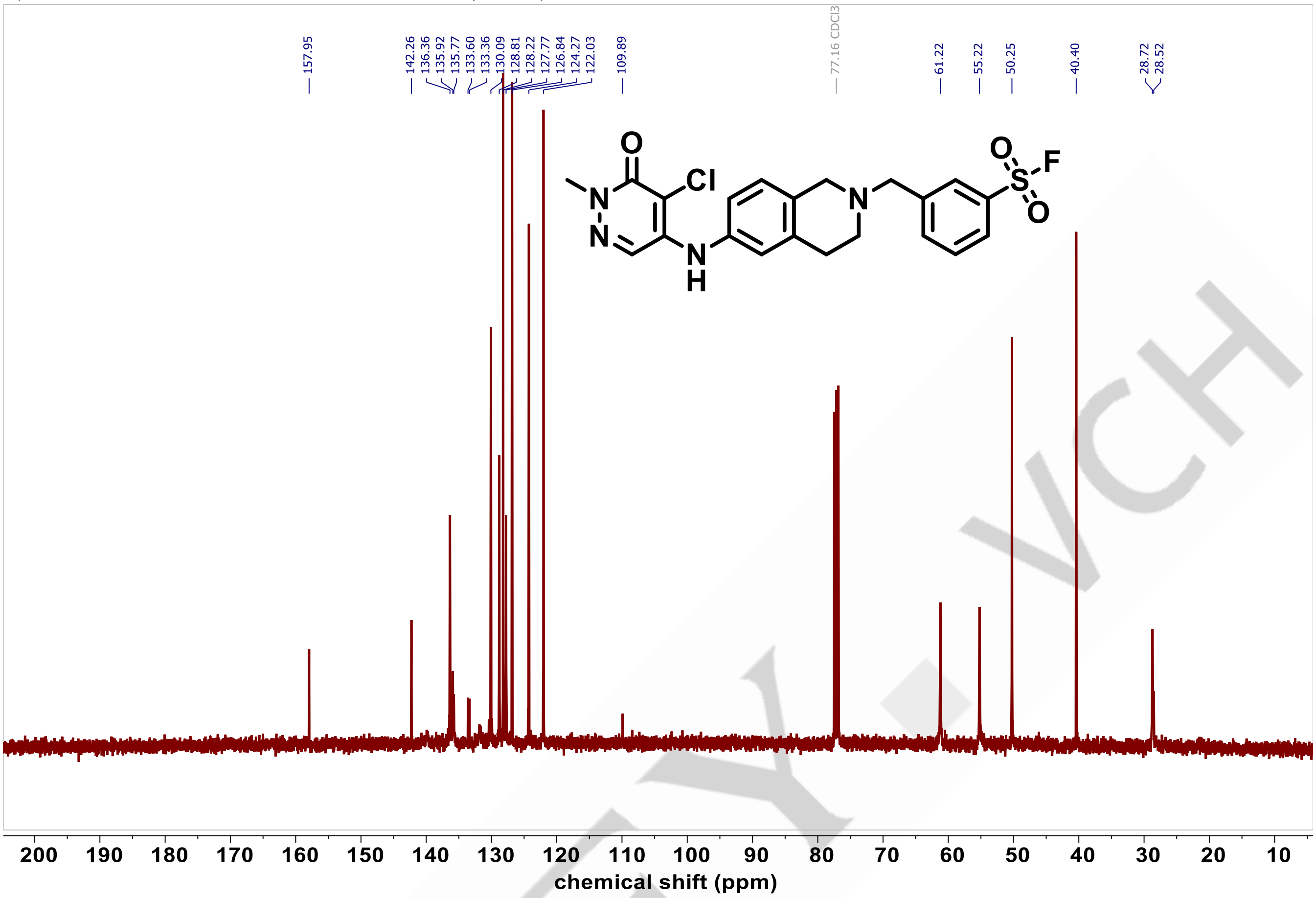

WILEY-VCH

11, ^{19}F-NMR, 376 MHz, $CDCl_3$

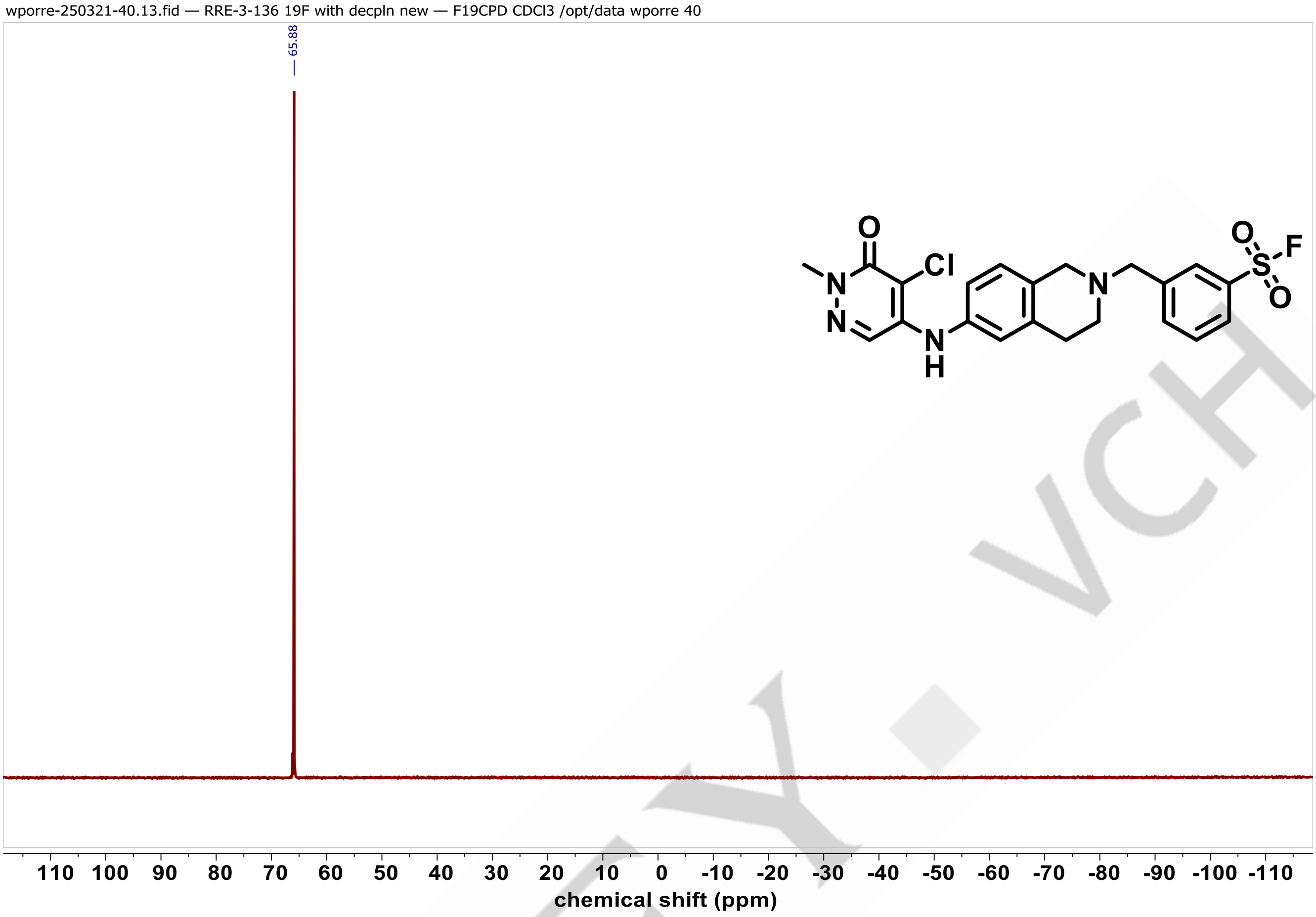

WILEY-VCH

12, ^{1}H-NMR, 400 MHz, $CDCl_3$

Raw.20.fid — akt 1 24 t2 — PROTON CDCl3 /opt/data wpoakt 38

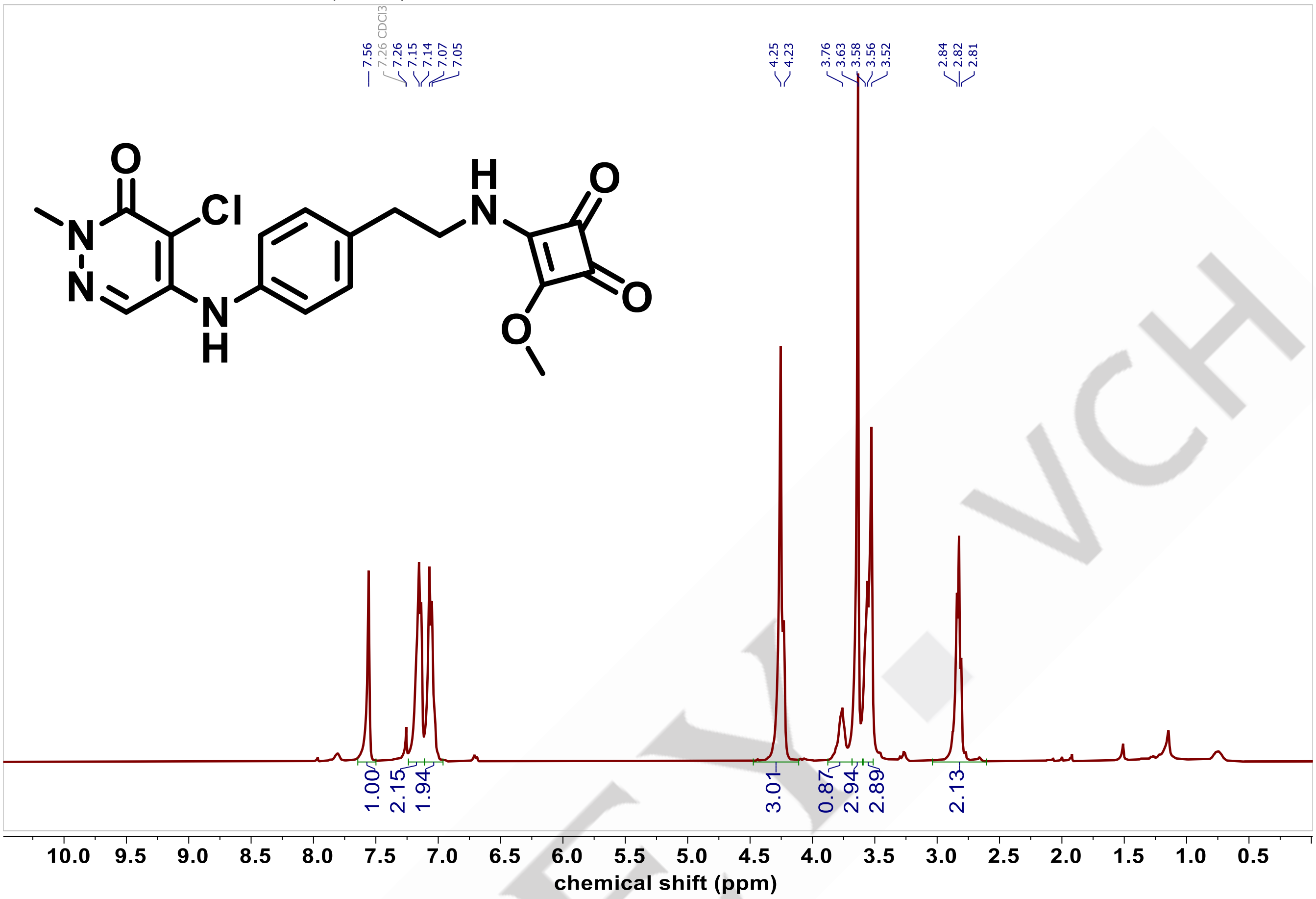

WILEY-VCH

12, ^{13}C-NMR, 101 MHz, $CDCl_3$

Raw/wpoakt-260406-38 AKT 1 24 13 C udeft — akt 1 24 t2 — C13udeft CDCl3 /opt/data wpoakt 38

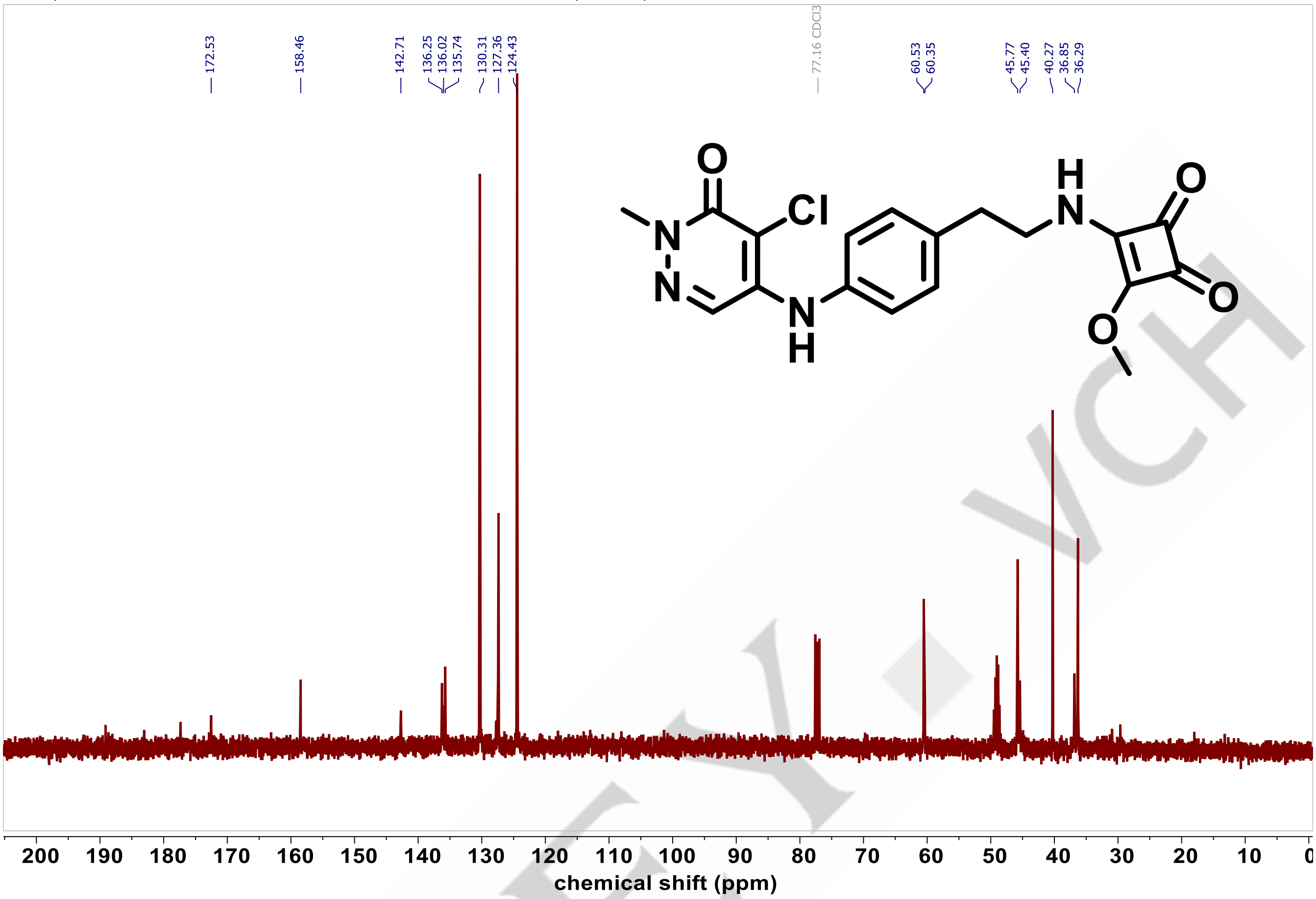

vii, ^{1}H-NMR, 400 MHz, $CDCl_3$

wporre-250604-50.10.fid — RRE-4-146 F4-F6 proton — PROTON CDCl3 /opt/data wporre 50

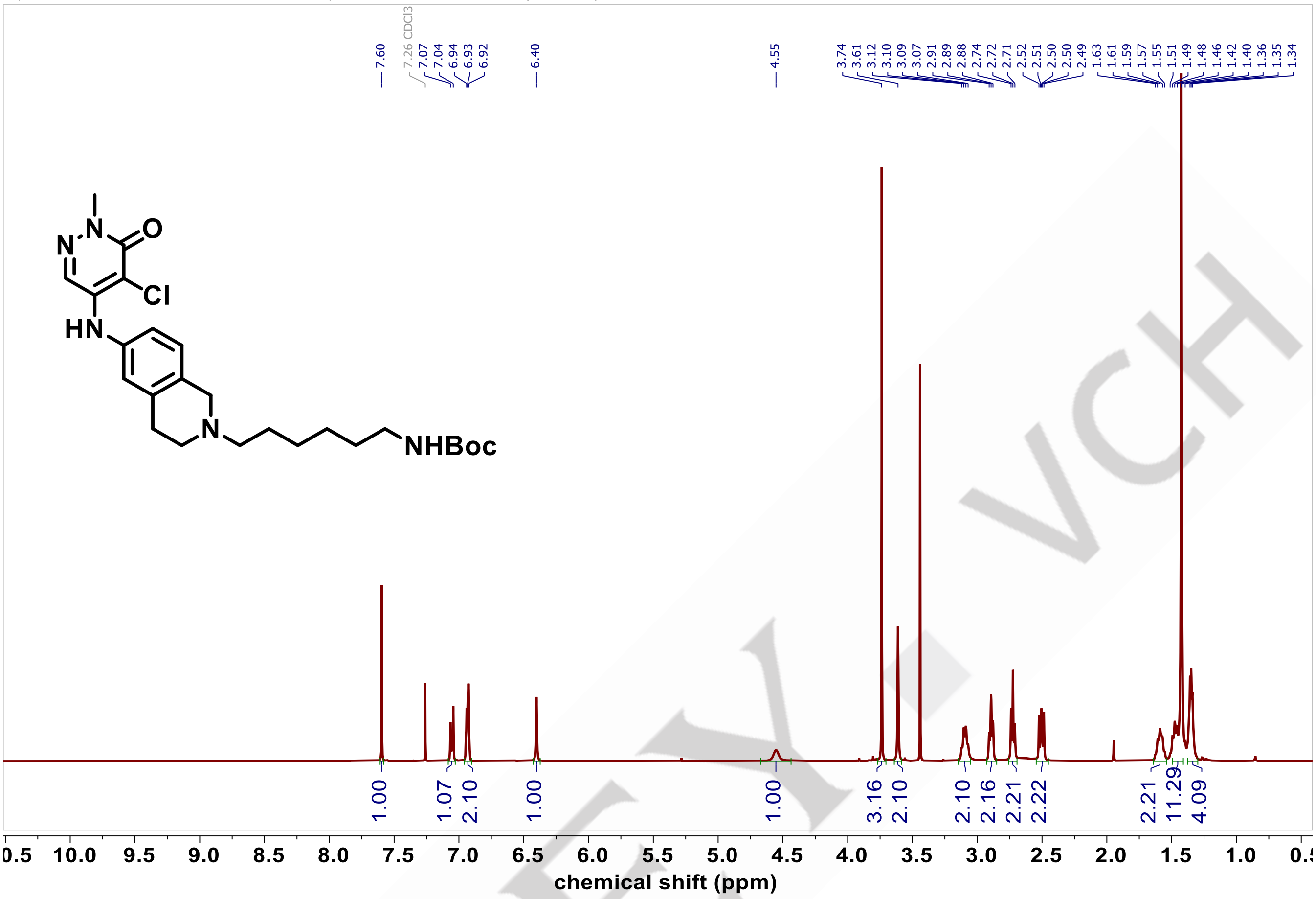

WILEY VCH

13, ^{1}H-NMR, 400 MHz, $CDCl_3$

wporre-250731-48.10.fid — RRE.4.148 — PROTON CDCl3 /opt/data wporre 48

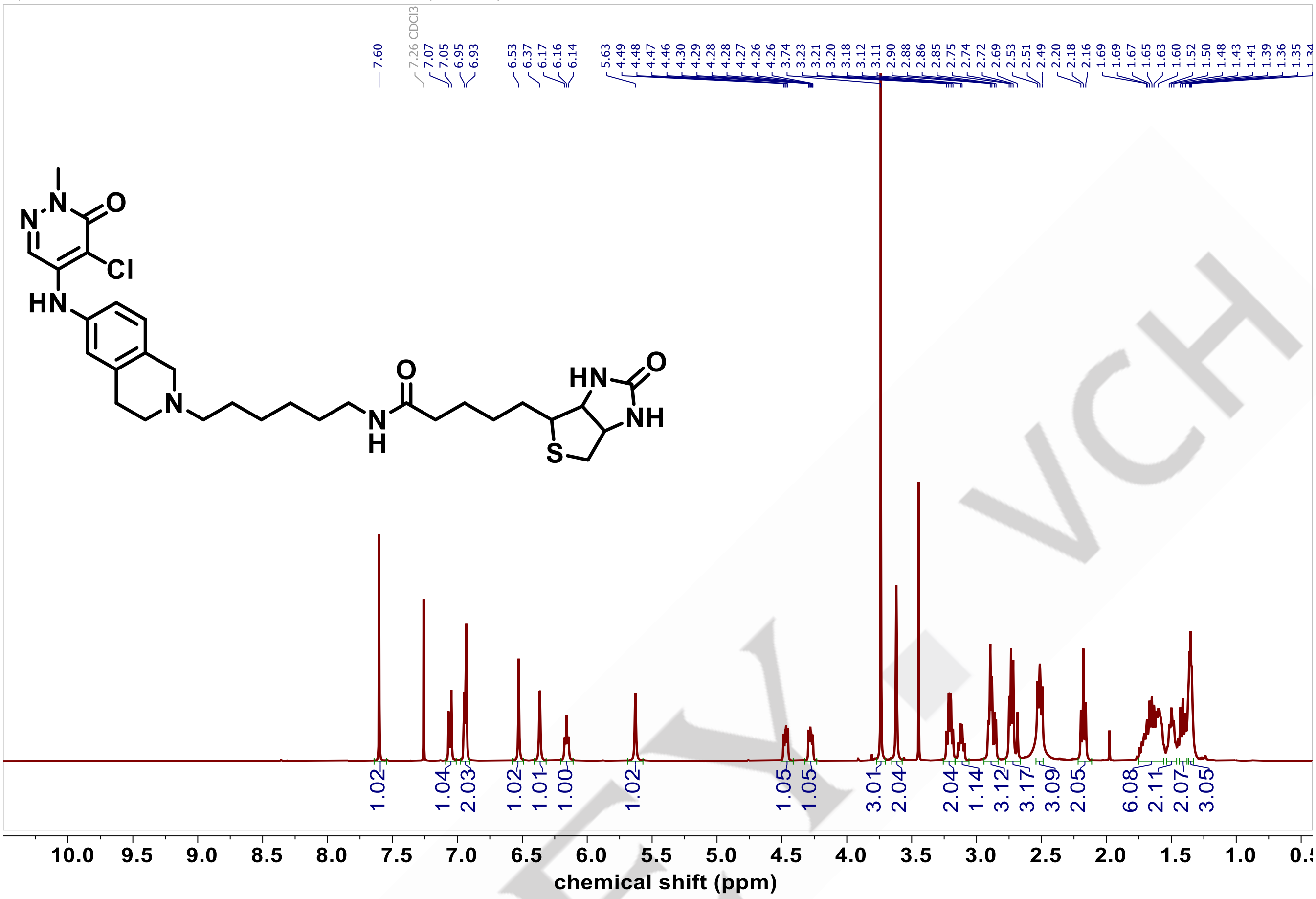

WILEY VCH
